# Predicting resistance to fluoroquinolones among patients with rifampicin-resistant tuberculosis: A cross-country validation study

**DOI:** 10.1371/journal.pmed.1004965

**Published:** 2026-09-15

**Authors:** Tianfang Shao, Mariana R. Neves, Molly Franke, Carole Mitnick, Jennifer Furin, Ted Cohen, Reza Yaesoubi

**Affiliations:** 1 Department of Epidemiology of Microbial Diseases, Yale School of Public Health, New Haven, Connecticut, United States of America; 2 Philip R. Lee Institute for Health Policy Studies, University of California San Francisco, San Francisco, California, United States of America; 3 Department of Global Health and Social Medicine, Harvard Medical School, Boston, Massachusetts, United States of America; 4 Department of Epidemiology and Biostatistics, University of California San Francisco, San Francisco, California, United States of America; PLOS Medicine Editorial Board, UNITED STATES OF AMERICA

## Abstract

**Background:**

Fluoroquinolones (FQs) are a cornerstone of most all-oral, shorter regimens endorsed by the World Health Organization for the treatment of rifampicin-resistant or multidrug-resistant tuberculosis (RR/MDR-TB). Knowledge of resistance to FQs can help guide regimen selection at the point of care. In settings where rapid testing for FQ resistance is unavailable, prediction models could support treatment decisions by identifying FQ resistance based on patient characteristics observable at the point of care. These prediction models have been typically developed and evaluated within a single country, and their generalizability across different geographic settings is unclear.

**Methods and findings:**

We used data from 5,175 patients with RR-TB and available FQ drug susceptibility testing (DST) results submitted to the TB Portals, an open-access data-sharing platform curated by the National Institute of Allergy and Infectious Diseases, from eight countries (Azerbaijan, Belarus, Georgia, Kazakhstan, Kyrgyzstan, Moldova, Romania and Ukraine) between 2012 and 2024. Among these patients, 1,772 (34.2%) had FQ-resistant TB. We developed prediction models for FQ resistance using logistic regression, neural networks, and XGBoost. Models were evaluated under three strategies: (1) *pooled models* trained on multi-country data; (2) *within-country models* trained and evaluated using internal validation; and (3) *cross-country models* trained on subsets of countries and externally validated on held-out countries. Model discrimination was assessed using the area under the receiver operating characteristic curve (AUROC) and the area under the precision-recall curve (AUPRC). Across the three algorithms, pooled models showed moderate optimism-corrected discrimination, with AUROC ranging from 0.70 to 0.72 and AUPRC ranging from 0.57 to 0.59. Within-country models demonstrated slightly better discrimination, with AUROC and AUPRC reaching 0.8 for some countries. Cross-country external validation showed that performance loss from using a model trained on external data could be negligible to >0.1 AUPROC or AUROC, depending on the country and algorithm. A limited set of predictors, including case definition and treatment-history-related variables, were among the most consistently informative predictors, whereas demographic, comorbidity, social-risk, education, and employment variables showed more variable contributions across countries and algorithms. A limitation of our study is that the incidence of RR-TB and FQ resistance was relatively stable in our analysis dataset. Hence, the results may not generalize to scenarios with marked changes in MDR-TB dynamics.

**Conclusions:**

Predicting FQ resistance using demographic and clinical characteristics showed moderate ability to identify FQ resistance among patients with RR-TB, but their performance and predictor patterns varied across countries. Models developed for one or several countries cannot be assumed to generalize to other settings without rigorous external validation. These findings highlight the limitations of globally trained prediction models for RR/MDR-TB and underscore the need for locally informed prediction models to support clinical decision-making.

## Author summary

**Why was this study done?:**

- Fluoroquinolones are key antibiotics used to treat rifampicin-resistant and multidrug-resistant tuberculosis (RR/MDR-TB), but they are not effective when the infecting strain is resistant to these drugs.
- Rapid laboratory tests for fluoroquinolone resistance are not available in many settings, making it difficult for clinicians to choose the most appropriate treatment regimen at the time of diagnosis.
- Prediction models based on patient characteristics have been proposed as an alternative, but most have been developed and evaluated in a single country, leaving it unclear whether they can be used reliably in other geographic settings.

**What Did the Researchers Do and Find?:**

- We analyzed data from more than 5,000 patients with RR-TB from eight countries in Eastern Europe and Central Asia, about one-third of whom had fluoroquinolone-resistant tuberculosis.
- We developed prediction models and compared models trained on data from multiple countries, models trained using data from individual countries, and models trained on data from multiple countries and tested in countries that were not used for model development.
- Models achieved only moderate overall accuracy. Models developed within a country generally performed better than those applied to new countries, although the magnitude of this difference varied substantially across settings.
- Previous tuberculosis treatment and disease history were among the most consistently useful predictors, whereas demographic and socioeconomic characteristics contributed less consistently across countries.
- The incidence of RR-TB and FQ resistance was relatively stable in our analysis dataset, and our findings may not generalize to settings with changing MDR-TB dynamics.

**What do these findings mean?:**

- Readily available clinical and demographic information can provide some indication of fluoroquinolone resistance when laboratory testing is unavailable, but prediction models alone are not sufficiently accurate to replace drug susceptibility testing.
- Prediction models developed in one country or region should not be assumed to work equally well elsewhere without external validation.
- These findings support the development and validation of locally adapted prediction models and reinforce the importance of expanding access to rapid drug susceptibility testing for patients with RR/MDR-TB.

## Abstract 3

In this cross-country study of more than 5,000 patients with rifampicin-resistant tuberculosis, Tianfang Shao and colleagues evaluate whether routinely available patient characteristics can help predict fluoroquinolone resistance when rapid testing is unavailable and find that model performance varies across countries, highlighting the importance of local validation before clinical use.

## Introduction

Tuberculosis (TB) remains one of the most pressing global health threats. In 2024, an estimated 10.7 million people developed TB, and 1.23 million died from the disease [1,2]. Despite progress in diagnosis and treatment, the burden of TB has been exacerbated by the rise of drug-resistant TB, particularly multidrug- or rifampicin-resistant TB (MDR/RR-TB) [3–5]. MDR/RR-TB is difficult to treat (with a success rate of 71% in 2022) and should be treated with second-line regimens, which include multiple antibiotics [1,6]. The selection of antibiotics for treating MDR/RR-TB should ideally be guided by DST. However, access to rapid and comprehensive DST remains limited in many high-burden settings, resulting in substantial delays in identifying resistance to second-line agents such as fluoroquinolones (FQs) [7]. FQs are a cornerstone of most all-oral, shorter regimens endorsed by the World Health Organization for the treatment of RR/MDR-TB [6]. However, FQ resistance continues to challenge efforts to treat MDR/RR TB; in 2024, an estimated 18% of MDR/RR TB cases globally were resistant to any FQs tested [1,8].

Recent studies have shown the potential of machine-learning approaches for predicting resistance to specific antibiotics using patient-level data. In Moldova, a clinical risk-based model for predicting resistance to FQs demonstrated reasonable discrimination (area under the receiver operating characteristic curve >0.8) using basic patient characteristics, including age, urban residence, and TB type [9]. Similarly, a study in Brazil developed prediction models for tuberculosis drug resistance using clinical and social characteristics from patients in São Paulo [10]. Together, these studies suggest that clinical and demographic information may contain usable signals for predicting resistance and that prediction models could enhance antibiotic selection in settings with limited or no access to DST.

A key limitation of existing studies is that they develop and evaluate resistance prediction models within a single country. Hence, little is known about whether these models generalize to other epidemiological contexts [9–11]. Geographic differences in social determinants, comorbidities, diagnostic pathways, and circulating resistance patterns may lead to substantial variation in predictor–outcome relationships, potentially limiting the transportability of models trained in one setting [12,13]. Understanding the extent to which prediction models can be effectively transferred across countries is essential before these tools can be meaningfully integrated into global TB programs.

To address this gap, we used records of patients with RR-TB from eight countries in the broader Eastern Europe and Central Asia region, provided by the TB Portals database [14], to systematically evaluate the within-country performance and cross-country transportability of three machine-learning models for predicting FQ resistance. Our analysis quantifies the impact of geographic heterogeneity on prediction performance and evaluates the transportability of these risk prediction models across geographic regions. Our findings offer insight into the feasibility and limitations of using clinical risk-based models to guide second-line therapy globally.

## Methods

### Overview

We first used data from each country to develop *country-specific* prediction models and evaluated their performance on the data from the same country. We then developed *cross-country* prediction models using data from N−1 countries and then evaluated their performance on the excluded country. Lastly, we used data from all countries to develop a *pooled model* to evaluate overall predictive performance and to describe how the final pooled model performed within each country. This design allows us to investigate three hypotheses: (1) models trained within individual countries would outperform pooled multi-country models when evaluated in the same country; (2) prediction performance would decline under cross-country external validation; and (3) the importance of individual predictors would vary across countries, reflecting geographic heterogeneity in the determinants of FQ resistance.

By examining the performance of within-country, cross-country, and pooled models, we aim to assess their robustness and transportability across diverse epidemiological contexts.

### Data sources and study populations

We conducted a secondary analysis of de-identified clinical data from TB Portals, an open-access multinational repository of tuberculosis case data curated by the National Institute of Allergy and Infectious Diseases (NIAID) [14]. TB Portals aggregates standardized, multi-domain patient records—including demographic, clinical, and DST data—from 13 countries, with an emphasis on drug-resistant TB cases. Data are collected as part of routine practice in TB clinics, research studies, and clinical trials. Some of the data originate from historical records, while others are collected prospectively. All data adhere to the HL7 Fast Healthcare Interoperability Resources standard and are validated by clinicians at entry, though variability in original collection protocols persists.

Our initial dataset included 17,297 TB cases from TB Portals. For our analysis, we focused on patients with confirmed RR-TB who had definitive DST results for FQs. Detailed resistance classification rules and preprocessing steps are provided in the S1 Appendix. The number of patients with RR-TB and FQ DST method and coverage among patients with RR-TB varied substantially across countries and over time (Tables A–B and Fig A in S1 Appendix). For this analysis, we included only country-level annual data with a relatively stable prevalence of RR-TB and FQ-DST coverage above 80%. The final sample included 5,175 RR-TB patients from eight countries: Kazakhstan, Belarus, Georgia, Moldova, Kyrgyzstan, Azerbaijan, Romania, and Ukraine, with registration dates between 2012 and 2024 (Table 1). Time series for the percentage of patients with RR-TB and FQ DST coverage among patients with RR-TB included in our analysis are displayed in Fig B in S1 Appendix.

**Table 1:** Country-specific inclusion of RR-TB patients with available fluoroquinolone DST results.

Country	Number of patients with RR-TB and DST results for FQ resistance	Proportion with FQ DST Results among patients with RR-TB (%)	Proportion of patients with FQ- resistant among RR-TB patients with FQ DST results (%)
**Kazakhstan**	1,193	96.1	20.6
**Belarus**	1,028	98.8	52.0
**Georgia**	966	98.3	30.6
**Moldova**	777	98	35.8
**Kyrgyzstan**	536	96.6	13.1
**Azerbaijan**	263	92.9	54.4
**Romania**	251	84.8	44.2
**Ukraine**	161	97	57.8

### Data preprocessing

To ensure consistency across countries and allow for cross-country validation, we used a prespecified common predictor set across the included countries and harmonized categorical and multi-response predictor values before modeling (S1 Appendix). We recorded missing or unreported categories as Unknown to keep them in the analysis. Missing continuous variables were imputed using median values within the modeling pipeline before model fitting [15]. Multi-response fields, such as social risk factors and comorbidities, were converted to binary indicators using one-hot encoding. A full description of resistance classification rules, recategorization rules, handling of multi-response variables, and imputation procedures is provided in the S1 Appendix.

### Prediction modeling frameworks

We developed and evaluated prediction models in accordance with the TRIPOD+AI statement for reporting clinical prediction models that use regression or machine learning methods (S1 Checklist) [16]. We considered three machine learning models: Logistic Regression, Neural Networks, and XGBoost. Logistic regression used L2-regularization and class-weighted loss functions. Neural networks were implemented as feed-forward architectures with multilayer perceptron functions, early stopping, and tuned hidden-layer size, regularization strength, and learning rate. XGBoost models were trained using gradient-boosted decision trees, with hyperparameters such as learning rate and tree depth tuned [17]. Full hyperparameter grids and model configurations are provided in the S1 Appendix.

All models were implemented as unified machine-learning pipelines to minimize information leakage. Numeric predictors were median-imputed and standardized, while categorical predictors were imputed and one-hot encoded. Feature selection was performed within the training pipeline using univariate SelectKBest with the ANOVA F statistic, with the number of selected features tuned during model selection. Country identifiers and outcome-related variables were excluded as predictors.

The primary model-selection criterion was the area under the precision-recall curve (AUPRC), given the prevalence-sensitive nature of fluoroquinolone resistance prediction, and the area under the ROC curve (AUROC) was also reported. To account for overfitting in the pooled and within-country models, we conducted bootstrap-based internal validation in line with TRIPOD recommendations, and the final configuration was selected based on the highest optimism-corrected AUPRC [16,18,19]. The details of this approach for calculating optimism-corrected measures are described in S1 Appendix.

We evaluated models under four complementary designs. (1) Pooled models were trained using data from all countries and internally validated using the bootstrap optimism correction approach (S1 Appendix). As a sensitivity analysis, we also evaluated pooled models using the 80/20 split design in which a model is trained on 80% of the data and evaluated on the remaining 20%. (2) We applied the pooled model to data from each country to obtain pooled-by-country estimates, which we interpret as descriptive summaries of country-specific performance rather than held-out assessments of geographic transportability. (3) We trained within-country models and internally validated them in each country using the bootstrap optimism correction approach. 4) We trained cross-country models using data from all countries except the held-out target country and externally evaluated the model in that country, with 95% confidence intervals estimated using 1,000 bootstrap resamples of the target-country data. The cross-country resampling design is described in S1 Appendix.

### Interpreting the contribution of predictors

We used three complementary approaches to interpret the contribution of predictors to model predictions. First, to enable comparison across logistic regression, neural network, and XGBoost models, we calculated permutation-based feature importance for all final models by randomly permuting each predictor and quantifying the resulting decrease in predictive performance measured by AUPRC. Predictors associated with larger decreases in performance after permutation were interpreted as contributing more strongly to model predictions.

For logistic regression models, we also present odds ratios by exponentiating the estimated coefficients. These odds ratios were used to describe the direction and magnitude of the association between each predictor and the predicted odds of FQ resistance. For neural network and XGBoost models, we used Shapley Additive exPlanations (SHAP) values to evaluate the contribution of each predictor to model predictions [20]. SHAP values estimate the contribution of each predictor to an individual-level prediction relative to the model’s average prediction. Positive SHAP values indicate predictors that increase the predicted probability of FQ resistance, whereas negative SHAP values indicate predictors that decrease the predicted probability. Because SHAP values are model-specific, we interpreted them primarily within each algorithm and modeling setting rather than as directly comparable effect sizes across algorithms or countries.

All interpretation analyses were conducted in Python. Permutation importance and logistic regression coefficients were obtained using *scikit-learn* [21], and SHAP values were estimated using the *shap* package [22].

### Ethics statement

This study involved secondary analysis of previously collected, de-identified data. The original data collection procedures were approved by the appropriate institutional review boards or ethics committees, and all data were obtained in accordance with relevant guidelines and regulations. The dataset used in this analysis contained no direct or indirect identifiers, and participants could not be re-identified by the study investigators.

### AI tools and technologies statement

ChatGPT (OpenAI, accessed June 2026) was used to assist with debugging portions of the analytical code and with proofreading the manuscript for grammar, spelling, and clarity. All AI-assisted code suggestions were independently reviewed and executed by the first author. All suggested language edits were reviewed and approved by all authors.

## Results

Our analysis included 5,175 individuals with RR-TB and confirmed FQ DST results from eight countries, of whom 1,772 (34.2%) had confirmed FQ resistance (Table 2). The prevalence of FQ resistance varied substantially across countries, ranging from 13.1% in Kyrgyzstan to 57.8% in Ukraine. In our final sample, most individuals were male (69.7%), newly diagnosed with TB (65.9%), and had pulmonary TB (92.8%). Compared with individuals with confirmed FQ susceptibility, those with FQ resistance were more often classified as treatment failure cases (21.8% versus 3.8%) and less often classified as newly diagnosed TB cases (36.8% versus 65.9%) (Table 2). Several demographic, clinical, and social risk factors were available for modeling, although multi-response variables, such as social risk factors and comorbidities, had substantial numbers of unknown or unspecified responses (Table 2 and Table C in S1 Appendix). Overall, the characteristics of individuals with RR-TB (*N* = 5,357) were broadly similar to those with RR-TB and DST results (*N* = 5,175) (Table C in S1 Appendix).

**Table 2:** Summary of clinical and demographic characteristics of patients with RR-TB who had a drug susceptibility testing (DST) result for FQ resistance in our dataset.

	Individuals with RR-TB and DST results for FQ resistance (*N* = 5,175)			
	Confirmed susceptibility to FQs (*N* = 3,403)		Confirmed resistance to FQs (*N* = 1,772)	
**Continuous Variable**	**Mean**	**SD**	**Mean**	**SD**
Age of onset	41.95	14.35	41.46	13.53
BMI	20.84	3.28	20.88	3.49
Number of children	0.76	1.25	0.48	0.98
Number of daily contacts	2.55	1.92	2.27	1.7
**Categorical Variable**	**Count**	**Percentage**	**Count**	**Percentage**
Sex				
Male	2,372	69.70%	1,277	72.07%
Female	1,031	30.30%	495	27.93%
Education				
Primary Education	798	23.45%	335	18.91%
Higher Education	1,020	29.97%	609	34.37%
Secondary Education	886	26.04%	502	28.33%
Unknown	690	20.28%	318	17.95%
No Education	9	0.26%	8	0.45%
Employment				
Unemployed	1836	53.95%	888	50.11%
Employed	791	23.24%	384	21.67%
Retired	256	7.52%	129	7.28%
Disabled	160	4.70%	239	13.49%
Unknown	97	2.85%	47	2.65%
Self-employed	162	4.76%	31	1.75%
Student	101	2.97%	54	3.05%
Case Definition				
New	2,244	65.94%	652	36.79%
Relapse	702	20.63%	448	25.28%
Unknown	159	4.67%	133	7.51%
Lost to follow-up	170	5.00%	152	8.58%
Treatment Failure	128	3.76%	387	21.84%
Lung Localization				
Pulmonary	3,157	92.77%	1,658	93.57%
Pulmonary and Extrapulmonary	246	7.23%	114	6.43%
Social Risk Factors*				
Unknown	1,333	39.17%	701	39.56%
Current smoker	1,133	33.29%	749	42.27%
Alcohol abuse	412	12.11%	303	17.10%
Drug abuse	70	2.06%	54	3.05%
Documented MDR contact	195	5.73%	127	7.17%
Ex prisoner	284	8.35%	204	11.51%
Homeless	71	2.09%	25	1.41%
Migrants	37	1.09%	8	0.45%
TB care worker	31	0.91%	11	0.62%
Worked abroad	261	7.67%	62	3.50%
Comorbidity*				
Unknown	1,787	52.51%	871	49.15%
Anemia	153	4.50%	79	4.46%
COVID-19	19	0.56%	10	0.56%
Post-COVID-19	15	0.44%	3	0.17%
Diabetes	317	9.32%	131	7.39%
HIV	238	6.99%	142	8.01%
Hepatic disease unspecified	22	0.65%	21	1.19%
Hepatitis B	25	0.73%	16	0.90%
Hepatitis C	253	7.43%	144	8.13%
Others	962	28.27%	594	33.52%
Psychiatric conditions	26	0.76%	14	0.79%
Renal disease	28	0.82%	6	0.34%
Immunosuppressive conditions	2	0.06%	1	0.06%

*Social risk factors and comorbidity were originally multi-categorical fields allowing multiple responses per patient; each category is encoded as a separate binary indicator, and each subcategory is summarized individually. Percentages indicate the proportion of patients with each risk factor and, as such, may not sum to 1.

**Fig 1:**
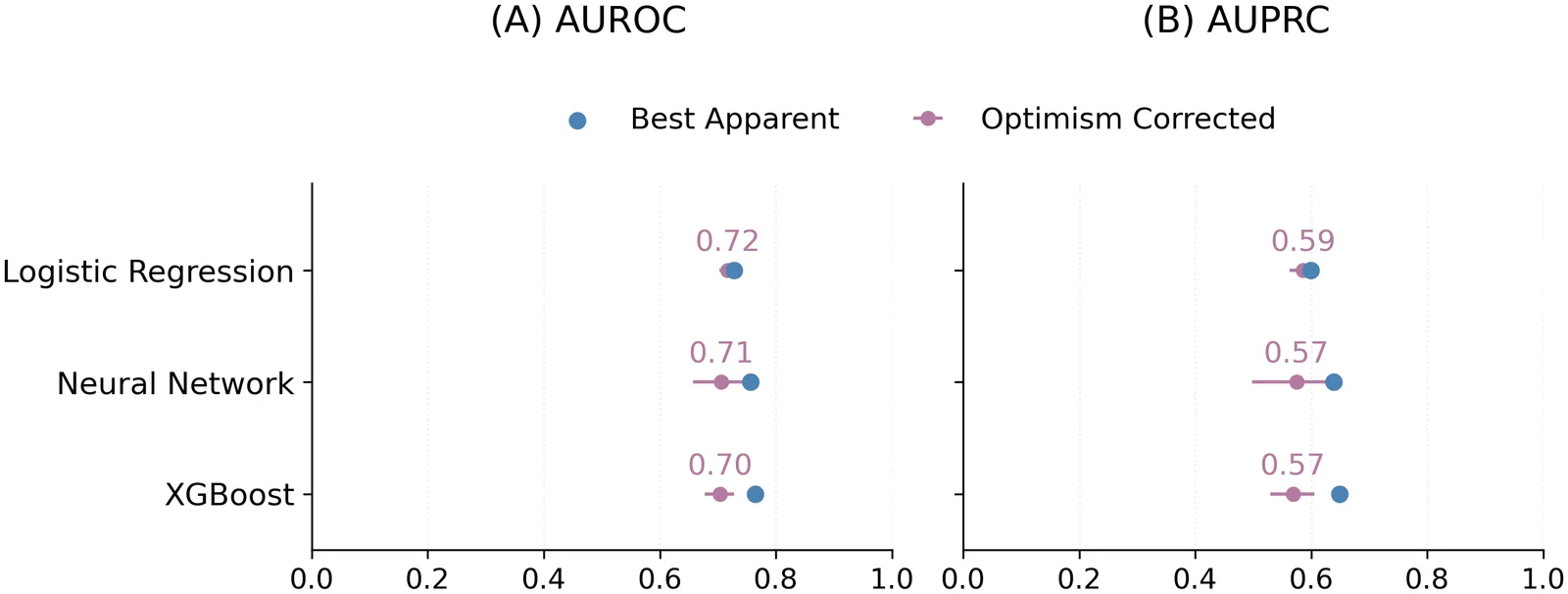
Apparent and optimism-corrected performance of pooled models trained on the entire dataset. Error bars represent 95% confidence intervals of the optimism-corrected estimates. AUROC: area under the receiver operating characteristic curve; AUPRC: area under the precision-recall curve. The performance of a model trained on 80% of the data and evaluated on the remaining 20% is presented in Fig C in S1 Appendix.

Pooled logistic regression, neural network, and XGBoost models, which are trained on the entire data from all included countries, showed moderate optimism-corrected discrimination (Fig 1). Optimism-corrected AUROC ranged from 0.70 to 0.72 across the three algorithms, while optimism-corrected AUPRC ranged from 0.57 to 0.59. Pooled logistic regression had the highest optimism-corrected AUROC and AUPRC, although differences across algorithms were small. The difference between the optimism-corrected and 80/20 split estimates was negligible (Fig C in S1 Appendix).

**Fig 2:**
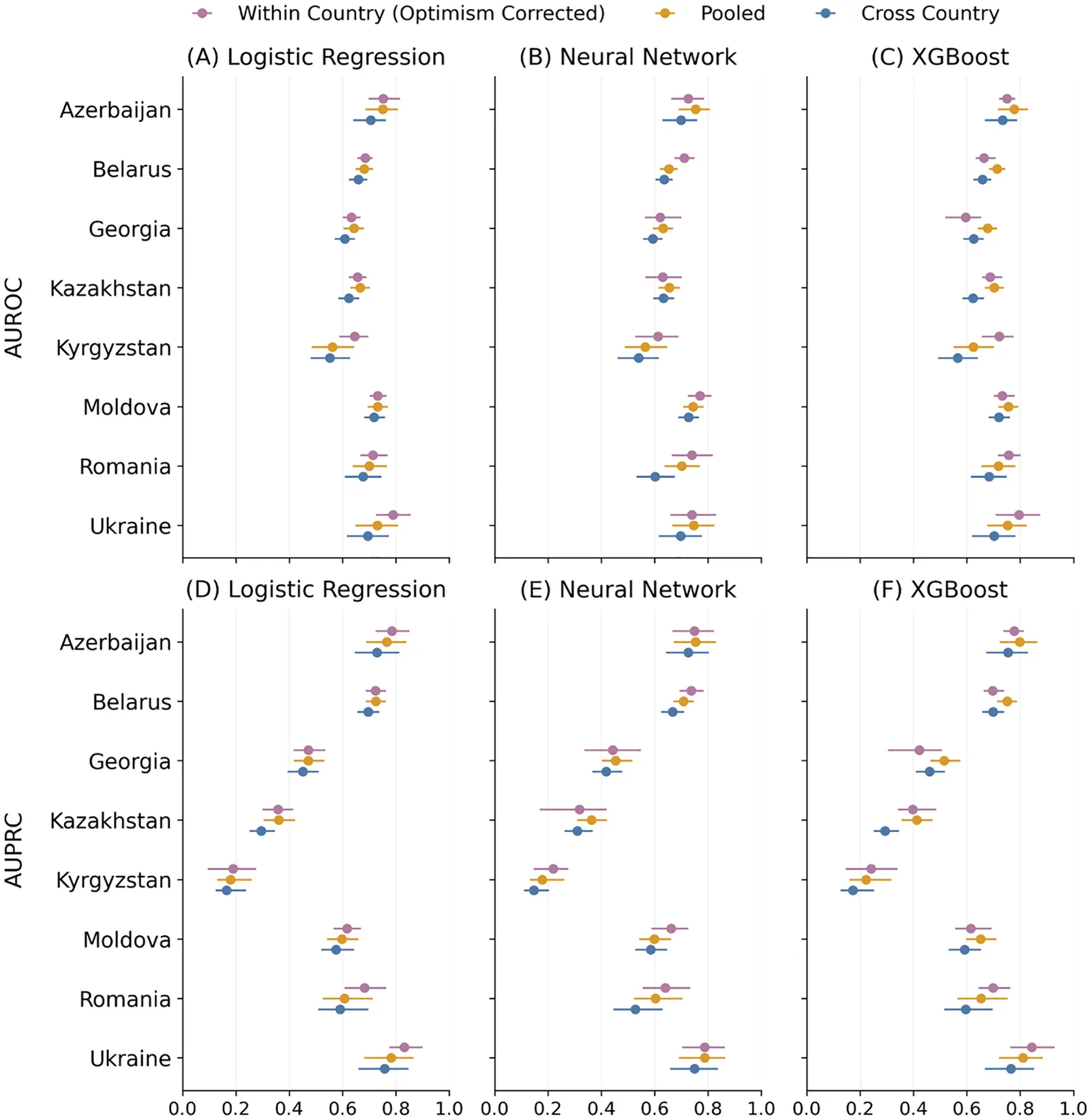
Comparing the performance of models that are trained on the entire dataset (the pooled model), trained and evaluated on the data from a single county (within-country models), and trained on data with one country excluded and assessed on the data from the excluded country (cross-country models). Error bars represent 95% confidence intervals. AUROC: area under the receiver operating characteristic curve; AUPRC, area under the precision-recall curve.

When the pooled model’s performance was examined by country, AUROC was relatively similar across countries (orange dots in Fig 2A–2C), whereas AUPRC varied more substantially between countries (orange dots in Fig 2D–2F). This difference was expected because AUPRC is more sensitive to the underlying prevalence of FQ resistance. Countries with lower FQ resistance prevalence, such as Kyrgyzstan and Kazakhstan, tended to have lower AUPRC values, even when AUROC was not markedly lower. In contrast, countries with higher FQ resistance prevalence, such as Ukraine and Azerbaijan, showed higher AUPRC values across several modeling strategies.

Across evaluation strategies, model performance varied by country, algorithm, and metric (Fig 2). Within-country models were often comparable to or slightly higher than the pooled models, particularly for logistic regression and XGBoost (comparing green dots with orange dots in Fig 2A and 2C). This pattern was less consistent for the neural network model, where the green, orange, and blue dots were closer together for several countries and confidence intervals were wider in some settings, such as Romania and Kazakhstan (Fig 2B). In the AUPRC panels, differences between strategies were more pronounced (Fig 2D–2F). Within-country models often had higher AUPRC than pooled or cross-country models, especially in countries with higher FQ resistance prevalence, such as Azerbaijan, Belarus, and Ukraine. However, this pattern was not uniform across all countries and algorithms; for example, in some lower-prevalence settings, including Kazakhstan, Kyrgyzstan, and Georgia, AUPRC remained relatively low across strategies. Cross-country models, evaluated on data from a country not included in the training set, performed slightly worse than the pooled models across all countries and modeling approaches (compare blue and orange dots in Fig 2).

**Fig 3:**
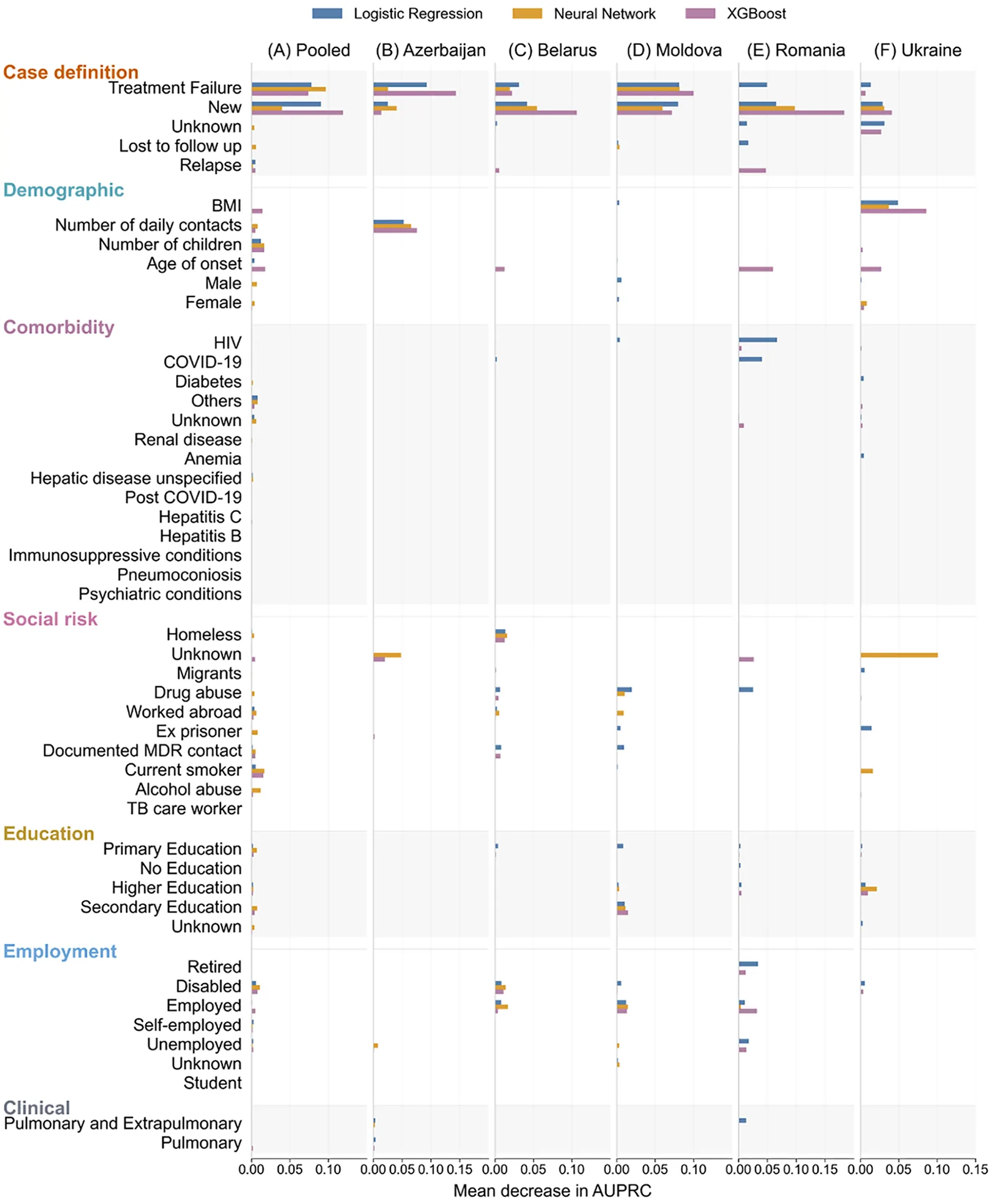
Permutation-based feature importance for pooled (Panel A) and within-country models (Panels B–F). For each potential predictor, bars indicate the mean decrease in area under the precision-recall curve (AUPRC) after exclusion. Larger decreases indicate a greater contribution of the feature to model discrimination. Georgia, Kazakhstan, and Kyrgyzstan were not included in the country-specific feature-importance panels because their country-specific model performance was less stable, making feature-importance interpretation less reliable.

Using a permutation-based feature importance method, we identified that for pooled models, case definition variables were the most consistently important predictors across algorithms, particularly new TB and treatment failure. This finding was supported by logistic regression odds ratios and SHAP analyses for the neural network and XGBoost models (Figs D–F in S1 Appendix). In the logistic regression models, treatment failure and other previously treated case definitions were associated with higher odds of FQ resistance, whereas new TB was generally associated with lower odds (Fig D in S1 Appendix), a conclusion also supported by SHAP values for neural network and XGBoost models (Figs E–F in S1 Appendix). Other variables, including BMI, number of daily contacts, number of children, comorbidity indicators, and selected social risk factors, showed smaller or less consistent contributions (Figs 3A and D–F in S1 Appendix). Country-specific models showed more heterogeneous patterns of feature importance. The case definition remained an important predictor in country-specific models, but the relative contributions of other predictors varied across countries (Figs 3B–3F and D–F in S1 Appendix). For example, the number of daily contacts contributed more strongly in Azerbaijan, whereas BMI and age of onset were more prominent in Ukraine (Fig 3, panels B and F). Some comorbidity, education, employment, and social risk variables also contributed in selected countries, although these patterns were not consistent across all algorithms or settings.

## Discussion

In this multi-county analysis, we evaluated the performance and generalizability of prediction models that use a patient’s basic demographic and clinical characteristics to identify resistance to FQs. We found that pooled models, trained on data from all countries, demonstrated moderate overall performance in detecting resistance to FQs (Fig 1). However, their performance varies across countries, particularly for AUPRC, which is sensitive to the underlying prevalence of FQ resistance (Fig 2). Models trained within individual countries achieved moderately better discrimination than models trained on pooled multi-country data or applied across countries. The performance loss from using a model trained on external data ranged from negligible to >0.1 AUPROC or AUROC, depending on the country and algorithm (Fig 2). These findings suggest limitations in the cross-country applicability of prediction models for identifying FQ resistance based on basic patient characteristics available at the point of care. Although these patient characteristics contain some information for identifying FQ resistance, their predictive power is shaped by the country context. Setting-specific factors, such as healthcare systems and epidemiological contexts, may influence the relationships between patient characteristics and FQ resistance. These setting-specific factors are typically not directly incorporated into individual-level risk prediction models, which may limit their transportability.

The countries included in our analysis are within the broader Eastern Europe and Central Asia region. Yet, our analysis indicated that FQ resistance prediction is context-dependent. Our analysis of permutation-based feature importance and SHAP values for pooled and within-country models showed that across all countries and algorithms, case definition (new or treatment failure) emerged as the only predictor that was consistently identified as highly informative (Figs 3, D–F in S1 Appendix). In contrast, the relative importance of other predictors, including BMI, number of daily contacts, comorbidity indicators, education, and employment status, varied between countries and algorithms (Figs 3, D–F in S1 Appendix). These differences suggest that the contribution of individual patient characteristics to resistance prediction may be influenced by local factors not directly incorporated into the risk prediction models. As such, individual-level risk prediction models may not provide universally stable predictors for MDR-TB.

Although these countries share several epidemiologic characteristics, including a high burden of RR/MDR-TB, the observed limitations in transportability may reflect multiple sources of heterogeneity. These include differences in the relationships between measured predictors and FQ resistance, variation in unmeasured factors such as prior fluoroquinolone exposure and treatment history, temporal changes in resistance patterns and treatment practices over the study period, and differences in resistance ascertainment methods. Because our analysis was not designed to distinguish among these mechanisms, the observed decline in cross-country performance should be interpreted as evidence that predictor–FQ resistance relationships are not fully stable across settings rather than as evidence for any single source of heterogeneity.

The limited advantage of neural network and XGBoost models over logistic regression is notable. One possible explanation is that the available predictors were relatively coarse clinical and demographic variables, rather than high-dimensional microbiological or genomic features. When the predictor set contains only a few strong signals, such as case definition or prior treatment history, flexible models may have little additional structure to learn. In addition, several predictors had substantial missingness or heterogeneous coding across countries, which may further reduce the benefit of complex algorithms. Therefore, our findings do not necessarily indicate that machine-learning methods are unsuitable for drug-resistance prediction, but rather that their added value may be limited when only routinely collected intake variables are available.

Previous studies on predicting drug-resistant tuberculosis have primarily focused on single-country settings and have relied on internal validation, limiting insights into model generalizability across populations [9,10]. In contrast, studies examining cross-country generalizability have primarily relied on genomic features rather than patient characteristics [23]. Our findings extend this literature by systematically comparing within-country, pooled multi-country, and cross-country prediction strategies using harmonized patient-level data from multiple countries. By evaluating transportability, our study demonstrates that while risk prediction models can perform well locally, their predictive relationships do not readily generalize across settings, highlighting an important limitation that has not been previously examined in a multi-country context.

Our study has several limitations. First, the incidence of RR-TB and FQ resistance was relatively stable in our analysis dataset, and hence, our findings may not generalize to scenarios with marked changes in MDR-TB dynamics. Second, although the analysis used a large multi-country dataset, the number of eligible RR-TB patients varied substantially across countries and years, and some country-specific estimates were based on smaller samples. This may have affected the stability of within-country performance estimates and feature-importance patterns. This concern is particularly relevant for neural networks and XGBoost, which may be more prone to overfitting smaller training sets. Third, we did not incorporate pathogen genomic data. While such data have been shown to improve resistance prediction, genomic sequencing remains unavailable or costly in many high-burden, resource-constrained settings. Our study intentionally focused on clinical and demographic predictors that are collected during patient intake, enabling treatment decisions at the point of care without requiring molecular testing infrastructure. Fourth, although the data were drawn from multiple countries, the final modeling workflow was restricted to a harmonized predictor set available across the selected countries. This restriction enabled pooled modeling and cross-country validation using a consistent feature space, but resulted in within-country models not utilizing all the variables that may have been available for individual countries. Fifth, among the 5,357 patients with RR-TB in our dataset, about 96.6% (*N* = 5,175) had DST results for FQ resistance. If DST is performed only for selected groups of patients (e.g., those with specific clinical or epidemiological risk factors), prediction models developed on this sample may not generalize to the entire population of patients with RR-TB. However, we observed a similar composition of patient characteristics among patients with RR-TB and patients with RR-TB and DST results (Table C in S1 Appendix), which could mitigate this issue. Finally, although FQ DST coverage was high in the selected analytical sample (Table 1), the diagnostic tests used for rifampicin and fluoroquinolone resistance determination differed across countries (Table A in S1 Appendix). Because testing practices varied by country, resistance ascertainment may not have been fully comparable across settings, potentially affecting generalizability.

Despite these limitations, the consistent patterns observed across countries strengthen the conclusion that predicting FQ resistance using patient characteristics alone can achieve reasonable accuracy but is inherently context-dependent. As such, prediction models trained and evaluated for a subset of countries may not be transportable to other countries. Future work can consider modeling approaches that explicitly account for geographic heterogeneity, such as hierarchical modeling frameworks that borrow strength across settings while preserving locally relevant risk relationships [24,25]. Future models may also benefit from combining routine demographic and clinical variables with microbiological, genomic, radiographic, treatment-history, and health-system-level predictors. These may include more granular prior TB and DR-TB treatment history, prior treatment outcomes, prior exposure to non-TB fluoroquinolones, hospitalization history, contact history with patients with pre-XDR or XDR-TB, and setting-level patterns of community- or over-the-counter-use of fluoroquinolones. Where rapid FQ DST is widely available, the incremental clinical utility of prediction models based only on demographic and clinical variables is likely limited. Their potential role may be greatest in settings where rapid second-line DST is unavailable, delayed, or inconsistently implemented, as interim triage tools while awaiting DST results, or for epidemiological risk stratification and program planning. More broadly, similar prediction approaches may also be relevant for newly introduced anti-TB drugs, particularly during early implementation when validated DST is not yet widely available, and the molecular basis of resistance remains incompletely understood [26].

## Supporting information

S1 Appendix Supplementary materials. (PDF)

S1 Checklist TRIPOD+AI Checklist for Prediction Model Development and Validation. Collins GS, Moons KGM, Dhiman P, et al. BMJ 2024;385:e078378. https://doi.org/10.1136/bmj-2023-078378.
(PDF)

S1 Data The study dataset. (CSV)

## Abbreviations

AUPRC: area under the precision-recall curve
AUROC: area under the ROC curve
DST: drug susceptibility testing
MDR/RR-TB: multidrug- or rifampicin-resistant TB
NIAID: National Institute of Allergy and Infectious Diseases
OCICB: Office of Cyber Infrastructure and Computational Biology

## References

[1] World Health Organization. Global Tuberculosis Report 2025. 2025.

[2] Goletti D, Meintjes G, Andrade BB, Zumla A, Lee S. Insights from the 2024 WHO Global Tuberculosis Report - more comprehensive action, innovation, and investments required for achieving WHO end TB goals. Int J Infect Dis. 2025;150:107325. doi: 10.1016/j.ijid.2024.107325 39631498

[3] Farhat M, Cox H, Ghanem M, Denkinger CM, Rodrigues C, Abd El Aziz MS, et al. Drug-resistant tuberculosis: a persistent global health concern. Nat Rev Microbiol. 2024;22(10):617–35. doi: 10.1038/s41579-024-01025-1 38519618

[4] Tan E-L, Qin Y, Yang J, Li X-J, Liu T-Q, Yang G-B, et al. Global burden of MDR-TB and XDR-TB: trends, inequities, and future implications for public health planning. BMC Infect Dis. 2025;25(1):1225. doi: 10.1186/s12879-025-11566-2 41039228 PMC12490063

[5] Dheda K, Barry CE 3rd, Maartens G. Tuberculosis. Lancet. 2016;387(10024):1211–26. doi: 10.1016/S0140-6736(15)00151-8 PMC11268880 26377143

[6] World Health Organization. WHO consolidated guidelines on tuberculosis. Module 4: treatment - drug-resistant tuberculosis treatment, 2022 update. World Health Organization; 2022. 36630546

[7] Nguyen TNA, Anton-Le Berre V, Bañuls A-L, Nguyen TVA. Molecular diagnosis of drug-resistant tuberculosis; a literature review. Front Microbiol. 2019;10:794. doi: 10.3389/fmicb.2019.00794 31057511 PMC6477542

[8] World Health Organization. Use of targeted next-generation sequencing to detect drug-resistant tuberculosis. 2023.

[9] You S, Chitwood MH, Gunasekera KS, Crudu V, Codreanu A, Ciobanu N, et al. Predicting resistance to fluoroquinolones among patients with rifampicin-resistant tuberculosis using machine learning methods. PLOS Digit Health. 2022;1(6):e0000059. doi: 10.1371/journal.pdig.0000059 36177394 PMC9518704

[10] Falcao IWS, Cardoso DL, Coutinho Dos Santos Santos AE, Paixao E, Costa FAR, Figueiredo K, et al. Model for predicting drug resistance based on the clinical profile of tuberculosis patients using machine learning techniques. PeerJ Comput Sci. 2024;10:e2246. doi: 10.7717/peerj-cs.2246 39650511 PMC11623081

[11] Wang S, Tu J. Nomogram to predict multidrug-resistant tuberculosis. Ann Clin Microbiol Antimicrob. 2020;19(1):27. doi: 10.1186/s12941-020-00369-9 32505203 PMC7276074

[12] Subbaswamy A, Saria S. From development to deployment: dataset shift, causality, and shift-stable models in health AI. Biostatistics. 2020;21(2):345–52. doi: 10.1093/biostatistics/kxz041 31742354

[13] Riley RD, Ensor J, Snell KIE, Debray TPA, Altman DG, Moons KGM, et al. External validation of clinical prediction models using big datasets from e-health records or IPD meta-analysis: opportunities and challenges. BMJ. 2016;353:i3140. doi: 10.1136/bmj.i3140 27334381 PMC4916924

[14] Rosenthal A, Gabrielian A, Engle E, Hurt DE, Alexandru S, Crudu V, et al. The TB portals: an open-access, web-based platform for global drug-resistant-tuberculosis data sharing and analysis. J Clin Microbiol. 2017;55(11):3267–82. doi: 10.1128/JCM.01013-17 28904183 PMC5654911

[15] Austin PC, White IR, Lee DS, van Buuren S. Missing data in clinical research: a tutorial on multiple imputation. Can J Cardiol. 2021;37(9):1322–31. doi: 10.1016/j.cjca.2020.11.010 33276049 PMC8499698

[16] TRIPOD+AI statement: updated guidance for reporting clinical prediction models that use regression or machine learning methods. BMJ. 2024;385:q902. doi: 10.1136/bmj.q902 38626948 PMC11019967

[17] Chen T, Guestrin C. XGBoost: a scalable tree boosting system. In: Proceedings of the 22nd ACM SIGKDD International Conference on Knowledge Discovery and Data Mining, San Francisco, California, USA, 2016. p. 785–94.

[18] Collins GS, Moons KGM, Dhiman P, Riley RD, Beam AL, Van Calster B, et al. TRIPOD+AI statement: updated guidance for reporting clinical prediction models that use regression or machine learning methods. BMJ. 2024;385:e078378. doi: 10.1136/bmj-2023-078378 38626948 PMC11019967

[19] Moons KGM, Altman DG, Reitsma JB, Ioannidis JPA, Macaskill P, Steyerberg EW, et al. Transparent Reporting of a multivariable prediction model for Individual Prognosis or Diagnosis (TRIPOD): explanation and elaboration. Ann Intern Med. 2015;162(1):W1-73. doi: 10.7326/M14-0698 25560730

[20] Ponce-Bobadilla AV, Schmitt V, Maier CS, Mensing S, Stodtmann S. Practical guide to SHAP analysis: explaining supervised machine learning model predictions in drug development. Clin Transl Sci. 2024;17(11):e70056. doi: 10.1111/cts.70056 39463176 PMC11513550

[21] Pedregosa F, Varoquaux G, Gramfort A, Michel V, Thirion B, Grisel O. Scikit-learn: machine learning in Python. J Mach Learn Res. 2011;12:2825–30.

[22] Lundberg SM, Lee SI. A unified approach to interpreting model predictions. 2017 [cited 2026 Dec 6]. Available from: https://arxiv.org/abs/1705.07874

[23] Babirye SR, Nsubuga M, Mboowa G, Batte C, Galiwango R, Kateete DP. Machine learning-based prediction of antibiotic resistance in *Mycobacterium tuberculosis* clinical isolates from Uganda. BMC Infect Dis. 2024;24(1):1391. doi: 10.1186/s12879-024-10282-7 39639222 PMC11622658

[24] Debray TP, Damen JA, Riley RD, Snell K, Reitsma JB, Hooft L, et al. A framework for meta-analysis of prediction model studies with binary and time-to-event outcomes. Stat Methods Med Res. 2019;28(9):2768–86. doi: 10.1177/0962280218785504 30032705 PMC6728752

[25] Van Calster B, McLernon DJ, van Smeden M, Wynants L, Steyerberg EW. Topic Group ‘Evaluating diagnostic tests and prediction models’ of the STRATOS initiative. Calibration: the Achilles heel of predictive analytics. BMC Med. 2019;17(1):230. doi: 10.1186/s12916-019-1466-7 31842878 PMC6912996

[26] Cross GB, Walker HN, Hasan T, Berry C. We are underprepared for bedaquiline resistance: a call for clinical and programmatic readiness. Clin Infect Dis. 2026. doi: 10.1093/cid/ciag008 41498255 PMC13341242

