# Supplementary material for: Predicting resistance to fluoroquinolones among patients with rifampicin-resistant tuberculosis: A cross-country validation study
Source: PLoS Med. 2026 Sep 15;23(9):e1004965. doi: 10.1371/journal.pmed.1004965 (PMC13588531; doi:10.1371/journal.pmed.1004965)
Supplement: Supplementary file 1 [file pmed.1004965.s001.pdf]

# Supplementary Materials

## S1. Data Cleaning and Preprocessing

### S1.1 Determination of Resistance to RIF and FQs

Resistance and sensitivity to rifampicin (RIF) and fluoroquinolones (FQs) were determined based on results from various diagnostic tests, including Bactec, Lowenstein-Jensen, Xpert MTB/RIF, Line-Probe Assay (LPA) (HAIN and related variants), and the Truenat test (Table ).

- Resistance classification: A case was classified as resistant to a drug if any available test indicated resistance.
- Susceptibility classification: If no test indicated resistance and at least one test reported susceptibility, the case was classified as susceptible.

### S1.2 Recategorization of Categorical Variables

For employment status, individuals who were self-employed or identified as homemakers were grouped and categorized as *Self-employed*. Multiple employment labels were harmonized into six categories: *Unemployed, Employed, Disabled, Retired, Student, Self-employed,* and *Unknown.*

Education levels were also simplified: *Basic school (including primary)* was recorded as *Primary Education*, *Complete school (A-level, gymnasium)* as *Secondary Education*, and both *College (bachelor)* and *Higher (university)* were combined into a single category labeled *Higher Education*.

Case definition types were grouped into five categories based on clinical relevance: *New, Relapse, Treatment Failure, Lost to follow-up,* and *Unknown.* Cases in lung localization with both *pulmonary and extrapulmonary* presentation were included in the *extrapulmonary* category.

### S1.3 Processing of Continuous Variables

Missing values in continuous variables were imputed based on their distributional characteristics. *Body Mass Index (BMI)* did not follow a normal distribution (Shapiro–Wilk test, $p < 0.001$)[1] and was therefore imputed using the median. For the *number of daily contacts* variable, non-numeric entries '>10' (3 cases from Moldova) were recoded as 10, and the variable was converted to numeric. Missing values were then imputed using the median.[2] Although both *the number of children* and *the number of daily contacts* are recorded as discrete count variables, they were modeled as continuous to retain their ordinal structure and maximize information use.

### S1.4 Multi-response Variables

Two variables, *social risk factors* and *comorbidities*, were initially recorded as comma-separated text fields. These variables were split into separate entries and then converted into binary indicator variables using one-hot encoding. Each subcategory, for example, *"Current smoker", "HIV"*, became a new column with values of 1 (present) or 0 (absent). Since patients could report multiple entries, the resulting subcategories are not mutually exclusive. This approach preserved the multi-response structure while enabling model compatibility.

## S2. Internal Validation via Bootstrap Optimism Correction

We followed a standard bootstrap-based approach to estimate and correct for model optimism in within-country and pooled models. Let AUC_app denote the apparent AUC computed on the full dataset. The bootstrap procedure involved:

1. Train the model on the full dataset and record AUC_app.
2. Repeat the following for N = 200 bootstrap iterations:
    1. Draw a bootstrap sample (same size as original, sampled with replacement).
    2. Train the model on the bootstrap sample.
    3. Compute AUC_boot on the bootstrap sample.
    4. Compute AUC_orig on the original dataset using the bootstrap-trained model.
    5. Compute optimism = AUC_boot − AUC_orig.
3. Average optimism across all N iterations to obtain mean_optimism.
4. Compute the optimism-corrected AUC (OC-AUC) as: OC-AUC = AUC_app - mean_optimism

This procedure follows TRIPOD recommendations and provides internally validated performance estimates.[3]

## S3. Cross-country Model Evaluation via Bootstrap Resampling

For cross-country evaluation, each target country was held out as test data, while the remaining *N*–1 countries formed the training set. The procedure is as follows:

1. Train the best model (selected via cross-validation) on pooled data from training countries.
2. Apply the trained model to the whole test country to obtain predicted probabilities.
3. Bootstrap evaluation (*N* = 1,000): For each iteration:
    1. Sample the test country with replacement.
    2. Compute AUROC or AUPRC on the bootstrap sample.
4. Estimate test performance as the mean of bootstrap AUROC values. Derive 95% confidence intervals from the 2.5th and 97.5th percentiles of the bootstrap distribution.

This approach yields robust estimates of external discrimination under realistic data variability.

## S4. Algorithm-specific Model Details

### S4.1 Common Modeling Pipeline

All algorithms were implemented using a unified machine-learning pipeline that included preprocessing, feature selection, and model fitting. Numeric predictors were median-imputed and standardized. Categorical predictors were imputed and one-hot encoded. Feature selection was performed within the modeling pipeline using univariate SelectKBest screening based on the ANOVA F statistic. Candidate values of k were 3, 5, 8, 10, 12, 15, 20, 25, 30, 35, 40, and all available transformed features. Candidate k values larger than the number of transformed features available in each training set were excluded. Selected features were re-estimated within each cross-validation fold and bootstrap resampling iteration, reducing the risk of information leakage.

### S4.2 Model Tuning and Evaluation

For each algorithm, candidate hyperparameter settings and candidate values of k were evaluated within the relevant training data. The primary model-selection metric was the AUPRC, with AUROC also reported. Hyperparameter tuning was performed using cross-validation within the training data. Permutation importance was computed using 50 permutations per feature.

### S4.3 Logistic Regression

Logistic regression models were fitted using L2-regularization with a maximum of 5,000 iterations. We considered inverse regularization strengths C = 0.01, 0.1, 1, and 10 and evaluated models with class-weighted loss. Logistic regression was included as an interpretable regularized linear benchmark for comparison with more flexible nonlinear models.

### S4.4 Neural Networks

Neural networks were implemented as feed-forward multilayer perceptrons. We considered hidden-layer sizes of 16, 32, 64, and 32 followed by 16 nodes; L2 regularization values $\alpha$ = 0.0001, 0.001, and 0.01; and initial learning rate of 0.001 and 0.0005. Early stopping was used where applicable, with a validation fraction of 0.15 to reduce overfitting during training. Neural networks were included to assess whether flexible nonlinear function approximation improved predictive performance relative to regularized logistic regression.

### S4.5 XGBoost

XGBoost models were trained using gradient-boosted decision trees. We considered 75, 150, and 300 trees; maximum tree depths of 2, 3, and 4; learning rates of 0.01, 0.03, 0.05, and 0.1; subsampling fractions of 0.8 and 1.0; column subsampling fractions of 0.8 and 1.0; minimum child weights of 1, 3, and 5; and L2 regularization values of 0.1, 1, 5, and 10. XGBoost was included to evaluate whether nonlinear tree-based interactions improved predictive performance relative to logistic regression and neural networks.

### S4.6 Model Selection

For pooled and within-country analyses, each candidate configuration, including algorithm-specific hyperparameters and the number of selected features, was evaluated using bootstrap optimism correction (Fig – Fig ). The final model was selected as the configuration with the highest optimism-corrected AUPRC. Pooled-by-country estimates were generated by applying the final pooled model within each country. These estimates were used as descriptive country-specific performance summaries.

For cross-country analyses, model selection was performed using only the training countries. The selected model was then refit on all training-country data and externally evaluated in the held-out country. Leave-one-country-out performance was summarized using 1,000 bootstrap resamples of the held-out country, with 95% confidence intervals based on the 2.5th and 97.5th percentiles of the bootstrap distribution.

## S5. Supplementary Tables

**Table A: DST methods to detect rifampicin and fluoroquinolone resistance in different countries. Methods are not mutually exclusive.**

| | RIF Diagnostic Tests (%) | | | | FQ Diagnostic Tests (%) | | | |
|---|---|---|---|---|---|---|---|---|
| **Country** | Bactec | Lowenstein-Jensen | HAIN | Xpert | Bactec | Lowenstein-Jensen | HAIN | Xpert |
| **Azerbaijan** | 54.3 | 81.8 | 8.7 | 65.3 | 37.3 | 72.8 | 1.2 | - |
| **Belarus** | 69.1 | 89.4 | 55.3 | 47.4 | 66.0 | 77.4 | 21.1 | 30.0 |
| **China** | 1.5 | 95.6 | 7.4 | - | - | 89.7 | 1.5 | - |
| **Georgia** | 51.1 | 51.8 | 71.0 | 81.7 | 20.7 | 9.1 | - | 18.4 |
| **India** | 81.3 | - | 50.0 | 75.0 | - | - | 12.5 | 18.8 |
| **Kazakhstan** | 69.7 | 2.8 | 22.9 | 76.1 | 93.7 | 2.5 | 2.7 | 27.0 |
| **Kyrgyzstan** | 92.6 | - | 93.2 | 85.9 | 92.1 | - | 0.8 | 84.5 |
| **Moldova** | 75.4 | 36.6 | 59.4 | 46.2 | 41.1 | 27.2 | 10.4 | 26.5 |
| **Nigeria** | - | 6.6 | 7.5 | 98.1 | - | 5.8 | - | 1.4 |
| **Romania** | 5.4 | 70.2 | 37.1 | 38.0 | 2.2 | 46.8 | 0.7 | 4.7 |
| **Senegal** | - | - | - | 93.4 | - | - | - | - |
| **South Africa** | 65.4 | - | 0.6 | 8.4 | 1.7 | - | 0.6 | - |
| **Ukraine** | 83.4 | 15.5 | 13.2 | 88.3 | 70.2 | 5.2 | 1.4 | 13.2 |

Note: Values represent the percentage of patients with each diagnostic test recorded for rifampicin or fluoroquinolone resistance determination. Because multiple diagnostic tests could be recorded for the same patient, percentages may sum to more than 100%.

**Table B: Prevalence of rifampicin (RIF) and fluoroquinolone (FQ) resistance among all 17,297 TB cases from TB Portals.**

| Country | Total Size | Number of Patients with RR-TB | Proportion of patients with RR-TB (%) | Number of patients with RR-TB and DST results for FQ resistance | Proportion with FQ DST Results among patients with RR-TB (%) | Number of patients with FQ-resistant TB | Proportion of patients with FQ-resistant among RR-TB patients with FQ DST results (%) |
|---|---|---|---|---|---|---|---|
| **Ukraine** | 6111 | 4835 | 79.1 | 4311 | 89.2 | 1548 | 35.9 |
| **Kazakhstan** | 1849 | 1244 | 67.3 | 1196 | 96.1 | 248 | 20.7 |
| **Belarus** | 1457 | 1118 | 76.7 | 1105 | 98.8 | 568 | 51.4 |
| **Georgia** | 3790 | 988 | 26.1 | 971 | 98.3 | 296 | 30.5 |
| **Moldova** | 1488 | 832 | 55.9 | 811 | 97.5 | 283 | 34.9 |
| **Kyrgyzstan** | 660 | 555 | 84.1 | 536 | 96.6 | 70 | 13.1 |
| **Azerbaijan** | 346 | 283 | 81.8 | 263 | 92.9 | 143 | 54.4 |
| **Romania** | 534 | 303 | 56.7 | 252 | 83.2 | 112 | 44.4 |
| **Nigeria** | 693 | 341 | 49.2 | 48 | 14.1 | 7 | 14.6 |
| **China** | 68 | 37 | 54.4 | 33 | 89.2 | 6 | 18.2 |
| **India** | 16 | 4 | 25.0 | 3 | 75 | 1 | 33.3 |
| **South Africa** | 179 | 1 | 0.6 | 1 | 100 | 0 | 0.0 |
| **Senegal** | 106 | 2 | 1.9 | 0 | 0 | 0 | - |

**Table C: Summary of clinical and demographic characteristics of patients with RR-TB.**

| | Individuals with RR-TB (N=5,357) | | Individuals with RR-TB and DST results for FQ resistance (N = 5,175) | |
|---|---|---|---|---|
| **Continuous Variable** | **Mean** | **SD** | **Mean** | **SD** |
| Age of onset | 41.73 | 14.1 | 41.78 | 14.07 |
| BMI | 20.86 | 3.35 | 20.85 | 3.35 |
| Number of children | 0.66 | 1.17 | 0.67 | 1.17 |
| Number of daily contacts | 2.45 | 1.85 | 2.45 | 1.85 |
| **Categorical Variable** | **Count** | **Percentage** | **Count** | **Percentage** |
| Sex | | | | |
| Male | 3779 | 70.54% | 3649 | 70.51% |
| Female | 1578 | 29.46% | 1526 | 29.49% |
| Education | | | | |
| Primary Education | 1175 | 21.93% | 1133 | 21.89% |
| Higher Education | 1697 | 31.68% | 1629 | 31.48% |
| Secondary Education | 1431 | 26.71% | 1388 | 26.82% |
| Unknown | 1034 | 19.30% | 1008 | 19.48% |
| No Education | 20 | 0.37% | 17 | 0.33% |
| Employment | | | | |
| Unemployed | 2818 | 52.60% | 2724 | 52.64% |
| Employed | 1214 | 22.66% | 1175 | 22.71% |
| Retired | 400 | 7.47% | 385 | 7.44% |
| Disabled | 414 | 7.73% | 399 | 7.71% |
| Unknown | 149 | 2.78% | 144 | 2.78% |
| Self-employed | 201 | 3.75% | 193 | 3.73% |
| Student | 161 | 3.01% | 155 | 3.00% |
| Case Definition | | | | |
| New | 3003 | 56.06% | 2896 | 55.96% |
| Relapse | 1190 | 22.21% | 1150 | 22.22% |
| Unknown | 310 | 5.79% | 292 | 5.64% |
| Lost to follow up | 326 | 6.09% | 322 | 6.22% |
| Treatment Failure | 528 | 9.86% | 515 | 9.95% |
| Lung Localization | | | | |
| Pulmonary | 4987 | 93.09% | 4815 | 93.04% |
| Pulmonary and Extrapulmonary | 370 | 6.91% | 360 | 6.96% |
| Social Risk Factors* | | | | |
| Unknown | 2092 | 39.05% | 2034 | 39.30% |
| Current smoker | 1945 | 36.31% | 1882 | 36.37% |
| Alcohol abuse | 730 | 13.63% | 715 | 13.82% |
| Drug abuse | 126 | 2.35% | 124 | 2.40% |
| Documented MDR contact | 334 | 6.23% | 322 | 6.22% |
| Ex prisoner | 499 | 9.31% | 488 | 9.43% |
| Homeless | 101 | 1.89% | 96 | 1.86% |
| Migrants | 45 | 0.84% | 45 | 0.87% |

| | | | | |
|---|---|---|---|---|
| TB care worker | 47 | 0.88% | 42 | 0.81% |
| Worked abroad | 339 | 6.33% | 323 | 6.24% |
| Comorbidity* | | | | |
| Unknown | 2761 | 51.54% | 2658 | 51.36% |
| Anemia | 238 | 4.44% | 232 | 4.48% |
| COVID-19 | 29 | 0.54% | 29 | 0.56% |
| Post-COVID-19 | 18 | 0.34% | 18 | 0.35% |
| Diabetes | 459 | 8.57% | 448 | 8.66% |
| HIV | 389 | 7.26% | 380 | 7.34% |
| Hepatic disease unspecified | 44 | 0.82% | 43 | 0.83% |
| Hepatitis B | 45 | 0.84% | 41 | 0.79% |
| Hepatitis C | 405 | 7.56% | 397 | 7.67% |
| Others | 1610 | 30.05% | 1556 | 30.07% |
| Psychiatric conditions | 40 | 0.75% | 40 | 0.77% |
| Renal disease | 34 | 0.63% | 34 | 0.66% |
| Immunosuppressive conditions | 3 | 0.06% | 3 | 0.06% |
| **FQ Result** | | | | |
| Resistant | 1772 | 33.08% | 1772 | 34.24% |
| Susceptible | 3403 | 63.52% | 3403 | 65.76% |
| Unknown | 182 | 3.40% | - | - |

* Social risk factors and comorbidity were originally multi-categorical fields allowing multiple responses per patient; each category is encoded as a separate binary indicator, and each subcategory is summarized individually. Percentages indicate the proportion of patients with each risk factor and, as such, may not sum to 1.

## S6. Supplementary Figures

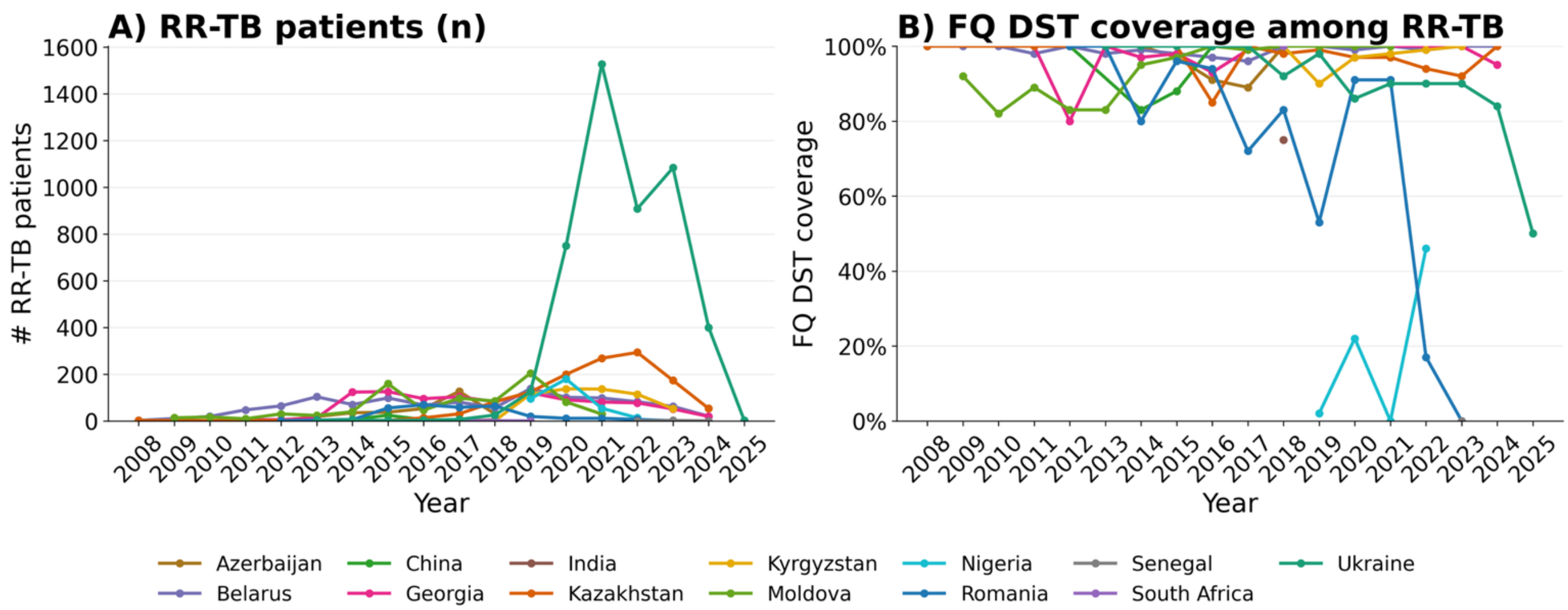


**Fig A. Annual RR-TB case counts and FQ DST coverage among patients with RR-TB in <u>the entire original dataset</u>, by country.** Note: Ukraine experienced a major increase in multidrug-resistant TB due to the war.[4]

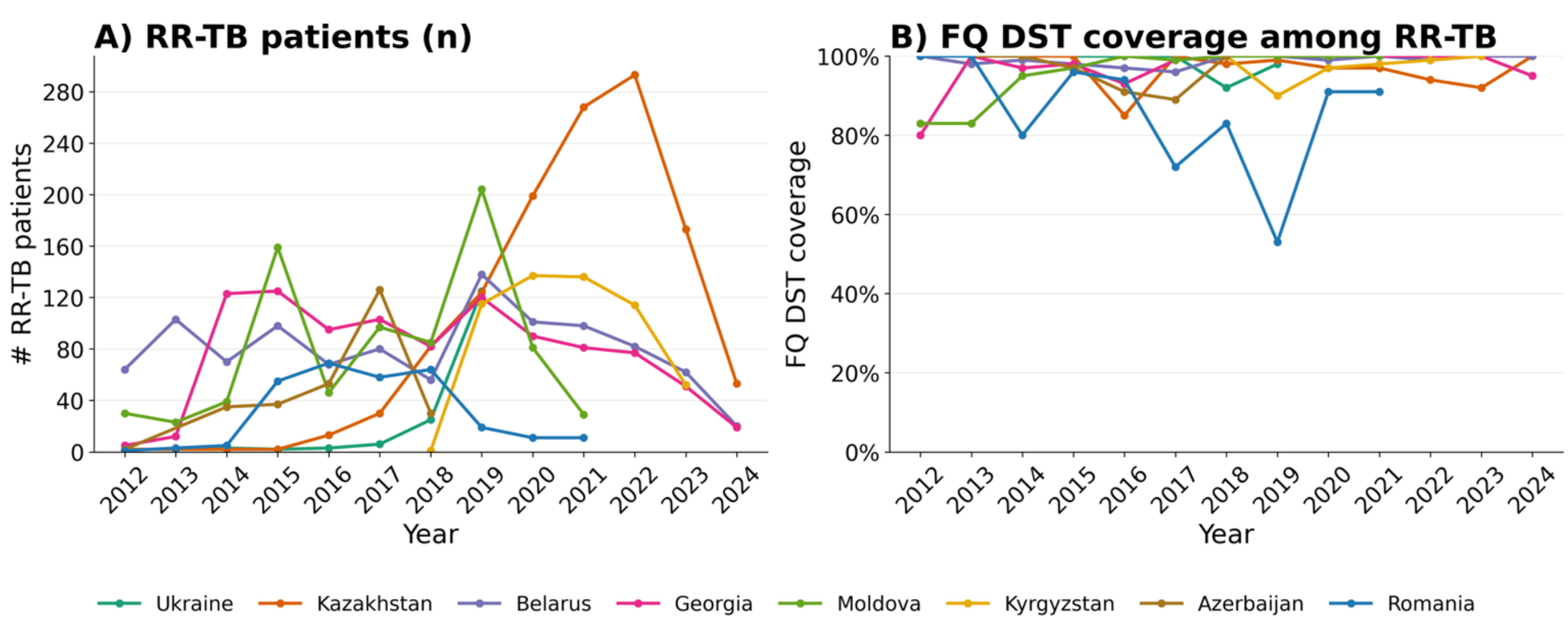


**Fig B. Annual RR-TB case counts and FQ DST coverage among patients with RR-TB in the <u>selected country-years included in the analysis</u>.** Data shown: Ukraine 2013-2019; Kazakhstan 2012, 2014-2024; Belarus 2012-2024; Georgia 2012-2024; Moldova 2012-2021; Kyrgyzstan 2018-2023; Azerbaijan 2012, 2014-2018; Romania 2012-2021.

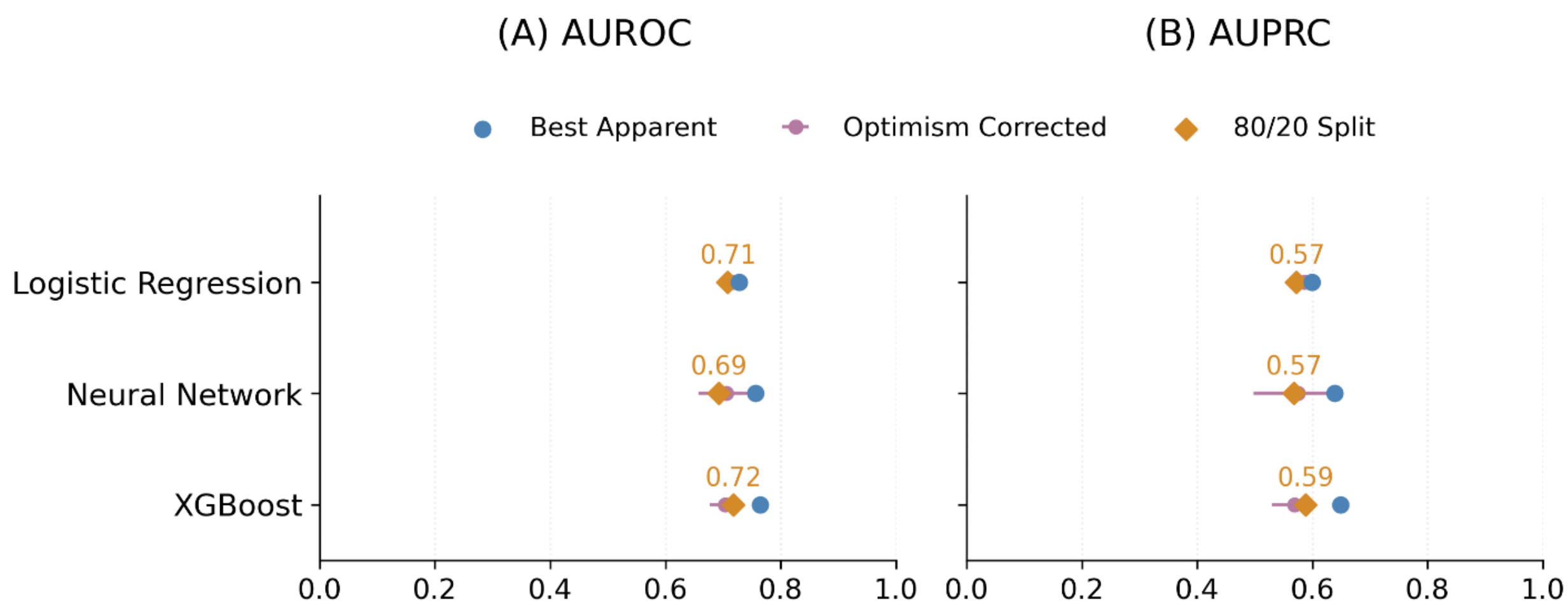


**Fig C. Apparent and optimism-corrected performance of pooled models trained on the entire dataset and the performance of a pooled model trained on 80% of the data and evaluated on the remaining 20% (80/20 split).** Error bars represent 95% confidence intervals of the optimism-corrected estimates. AUROC: area under the receiver operating characteristic curve; AUPRC: area under the precision-recall curve.

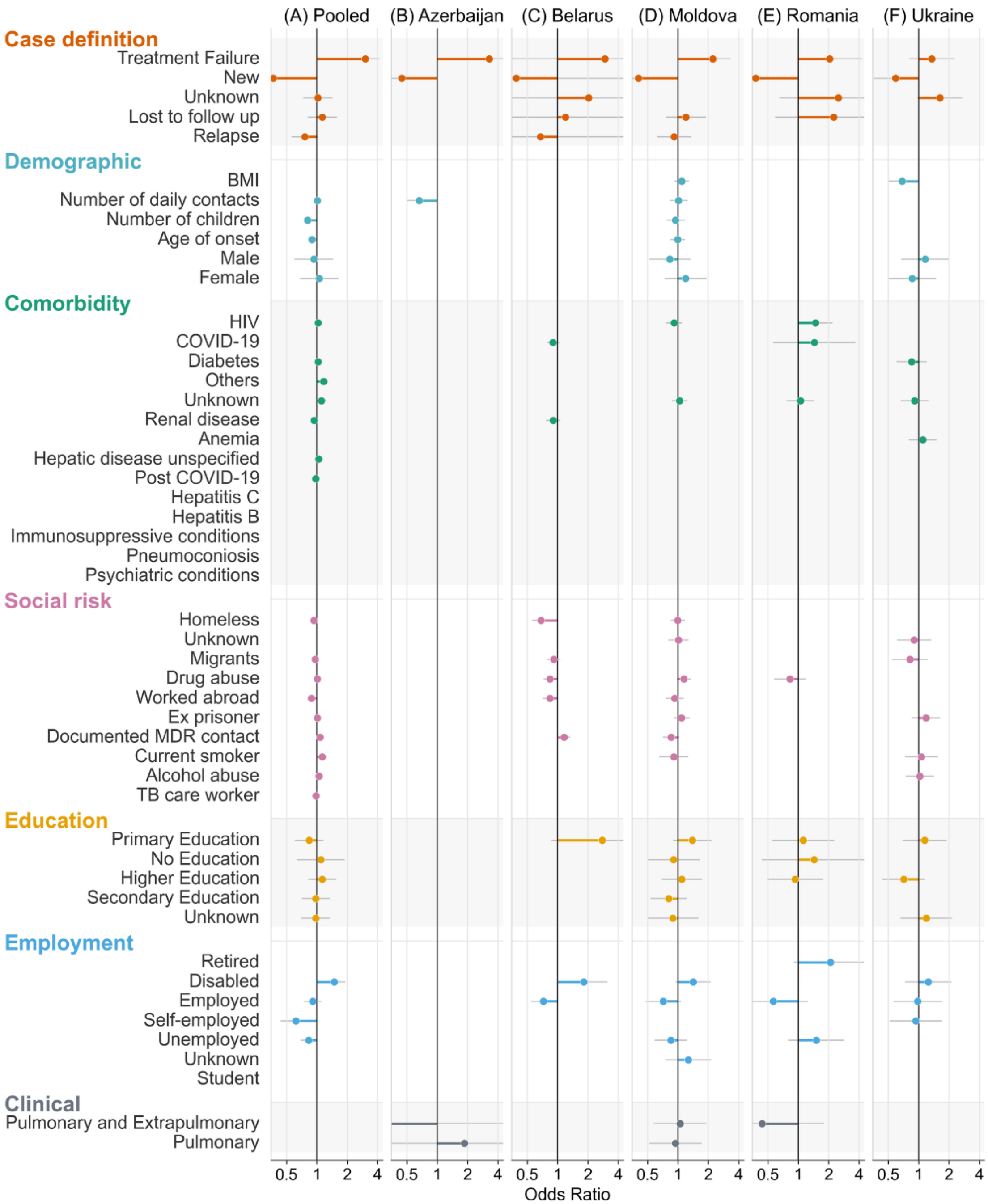


**Fig D. Logistic regression odds ratios for selected predictors of FQ resistance across pooled and country-specific models.**

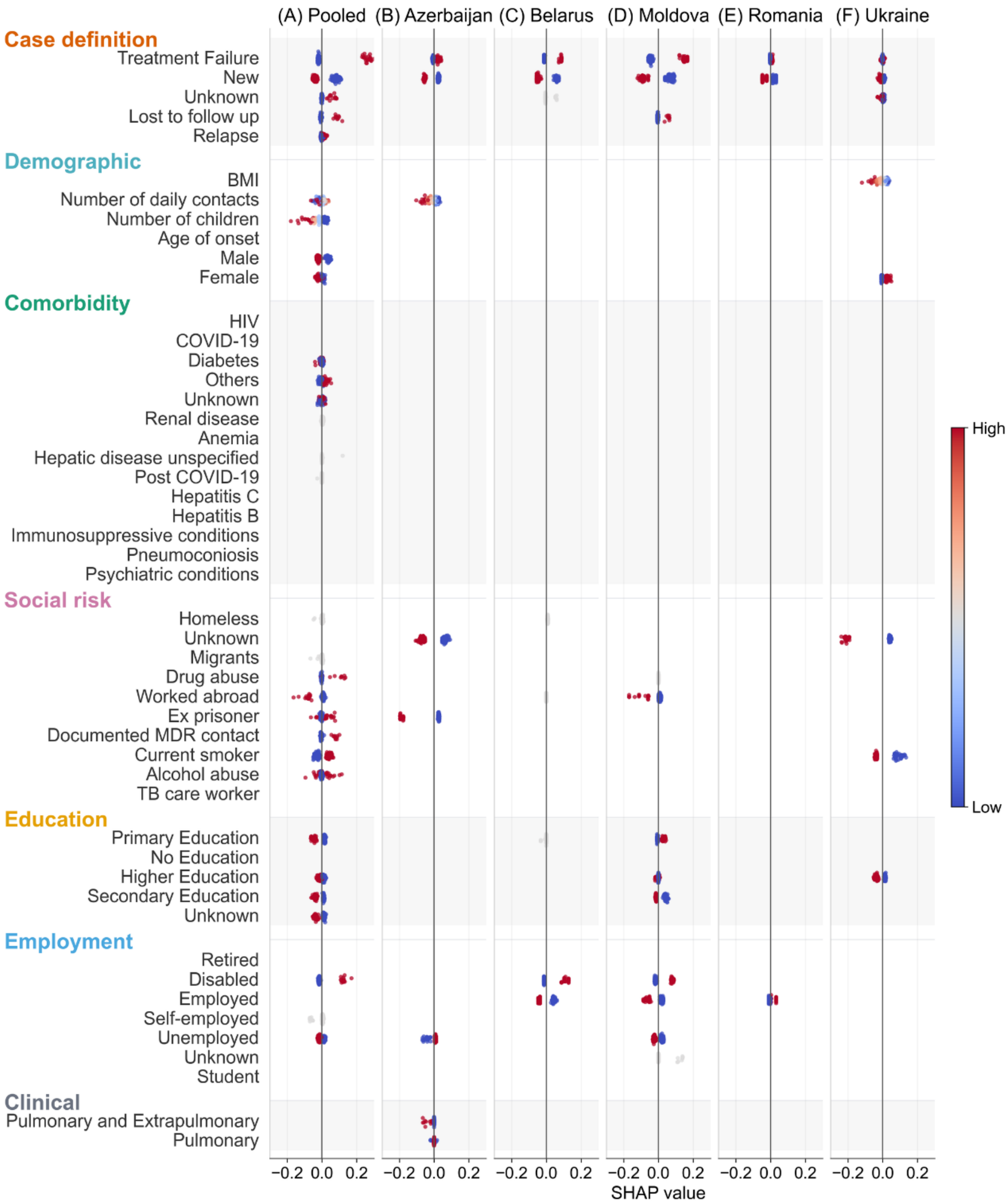


**Fig E. Neural network SHAP values for selected predictors of FQ resistance across pooled and country-specific models.**

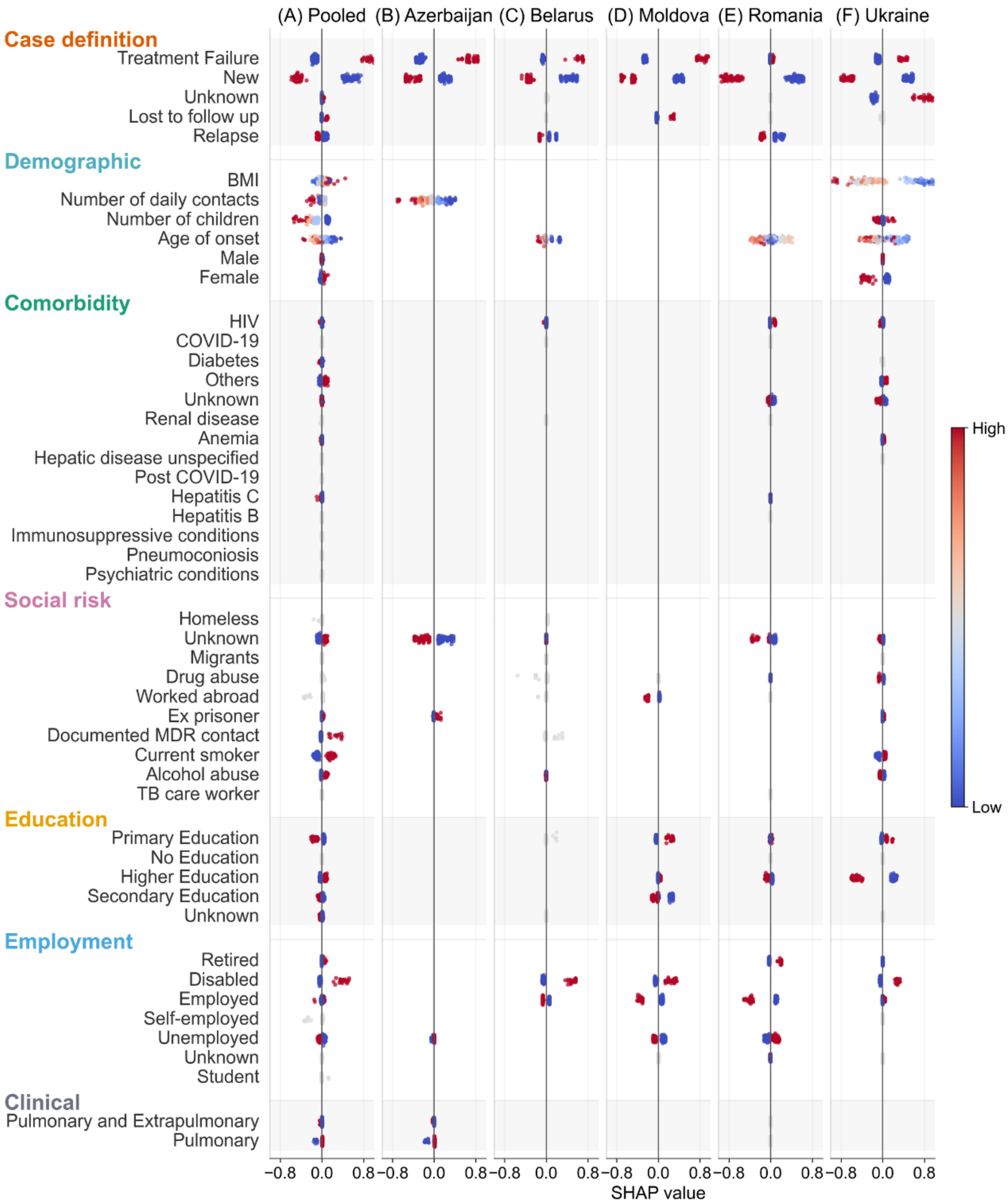


**Fig F. XGBoost SHAP values for selected predictors of FQ resistance across pooled and country-specific models.**

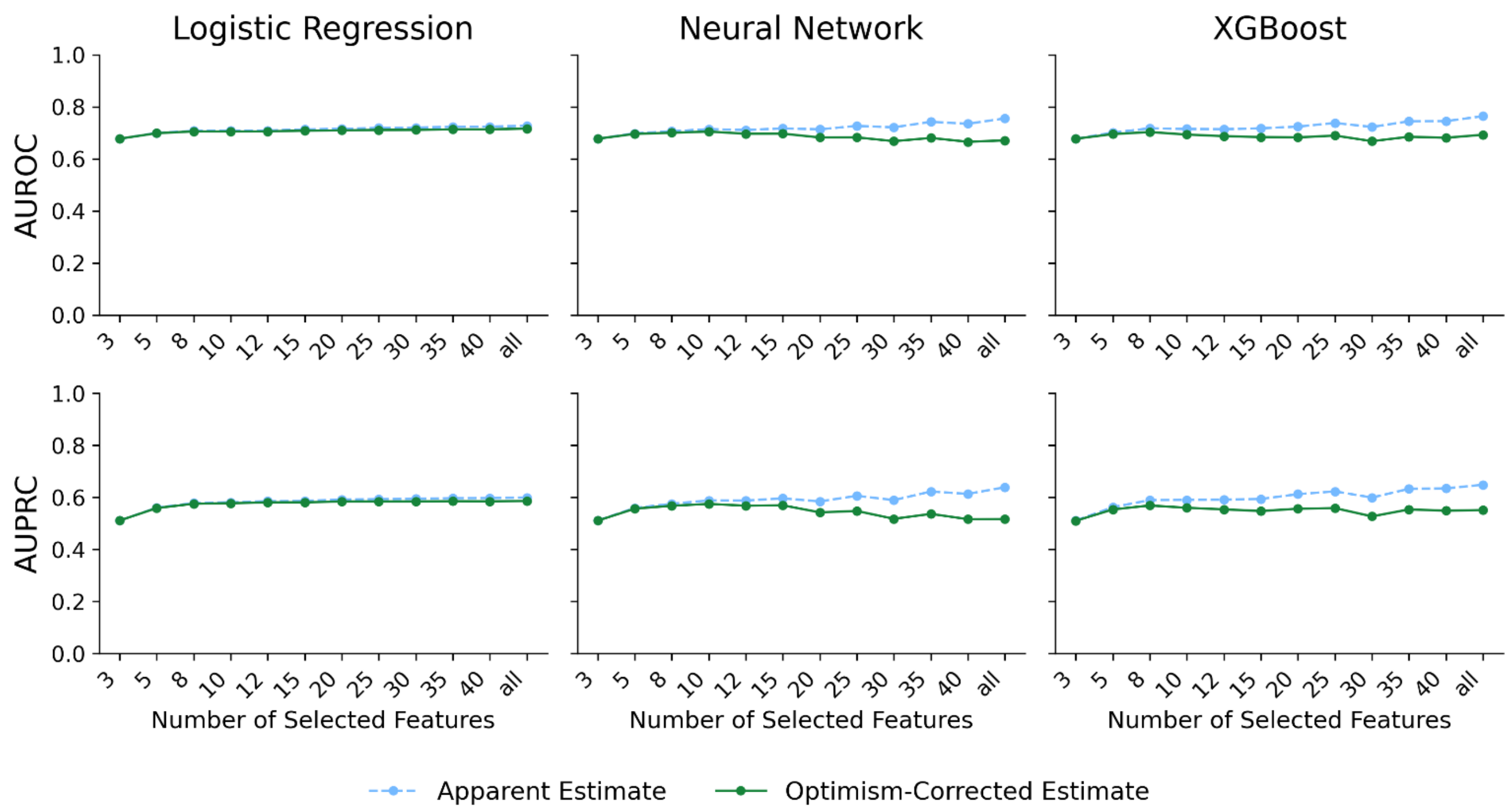


**Fig G. Performance of the pooled model (trained on the entire dataset) by the number of selected features across three algorithms.**

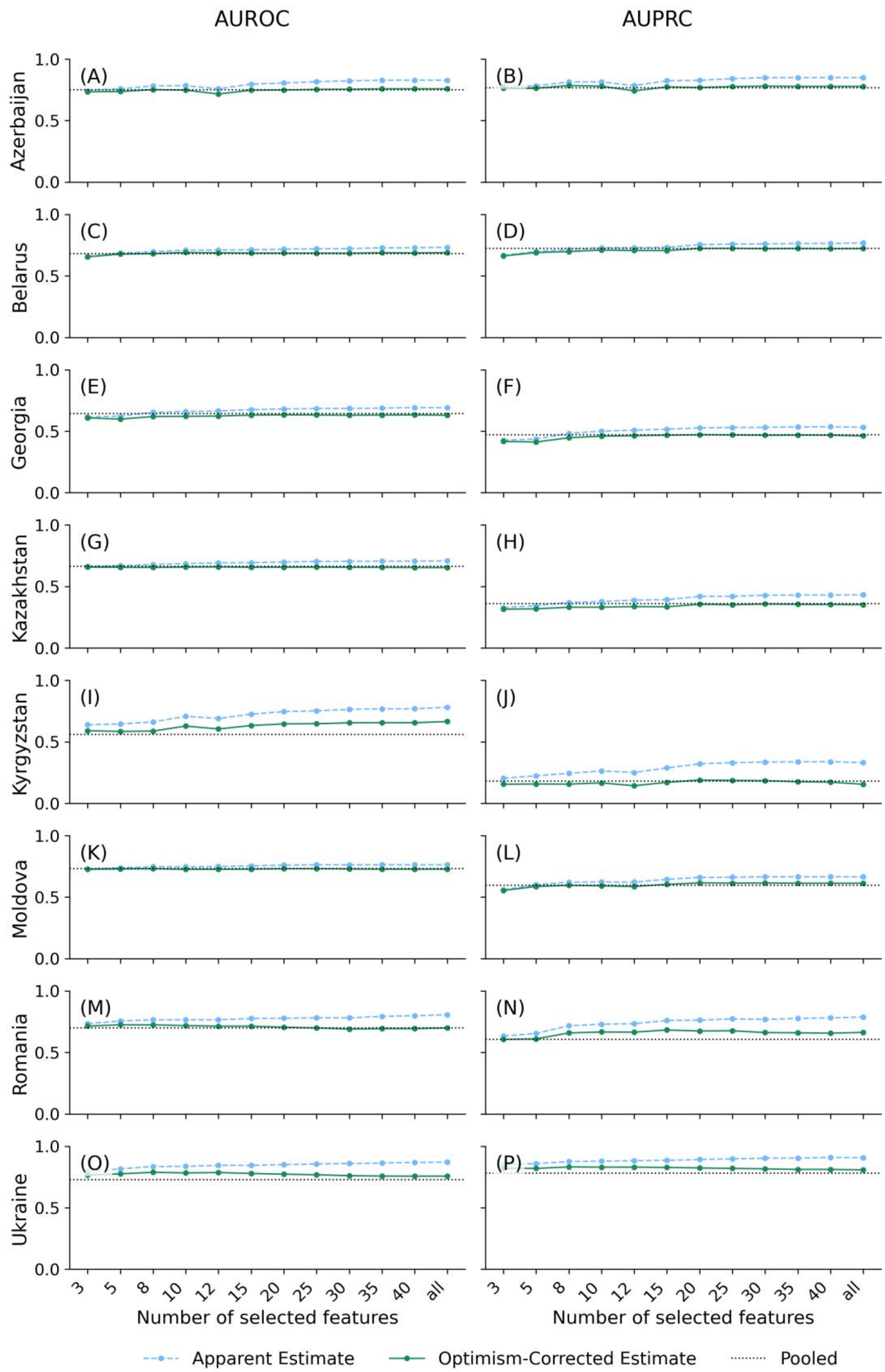


**Fig H. The performance of within-country logistic regression models by the number of selected features.** The dotted horizontal line indicates the performance of the pooled model applied to each country.

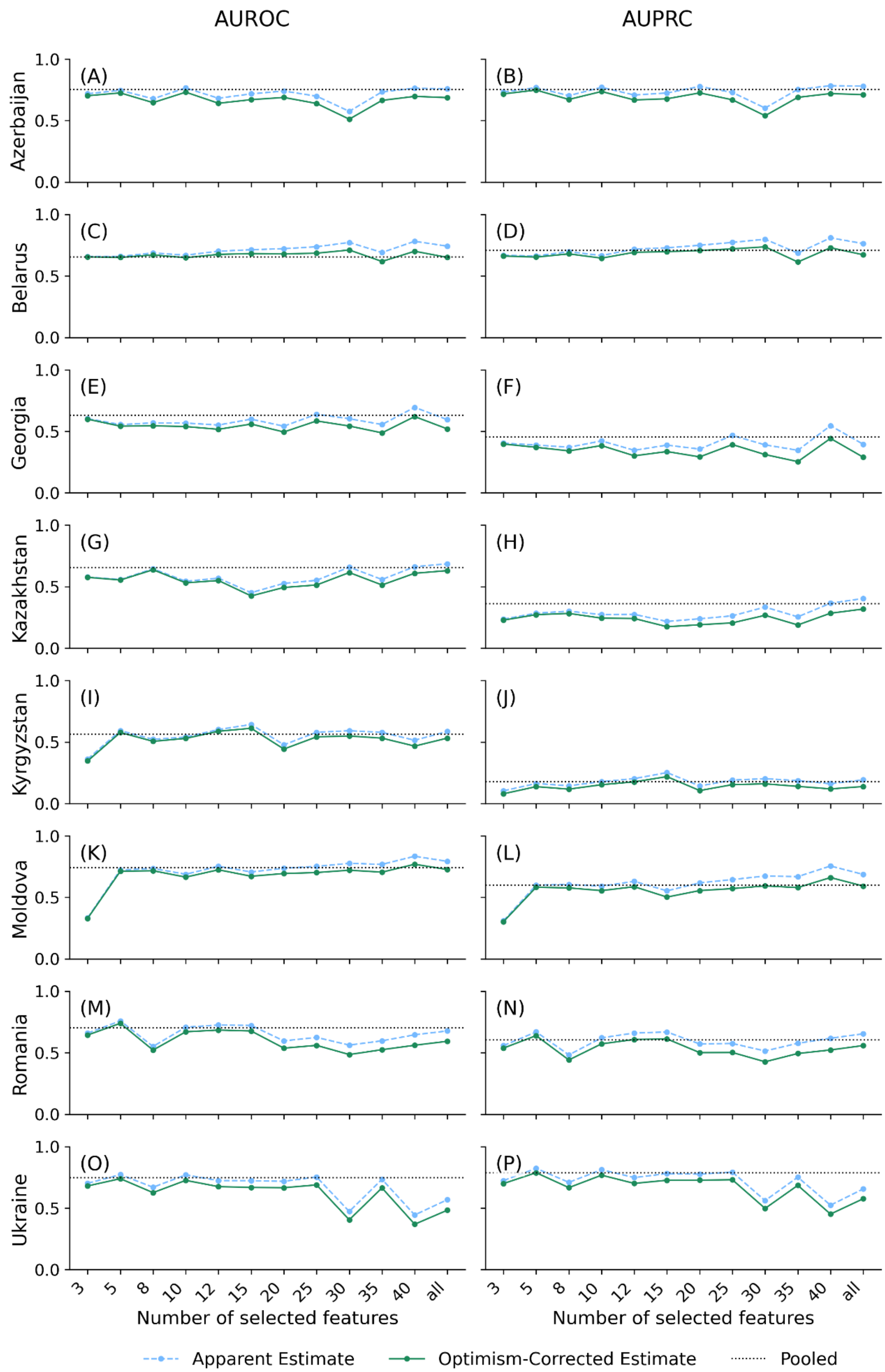


**Fig I. The performance of within-country neural network models by the number of selected features.** The dotted horizontal line shows the pooled model's performance for each country.

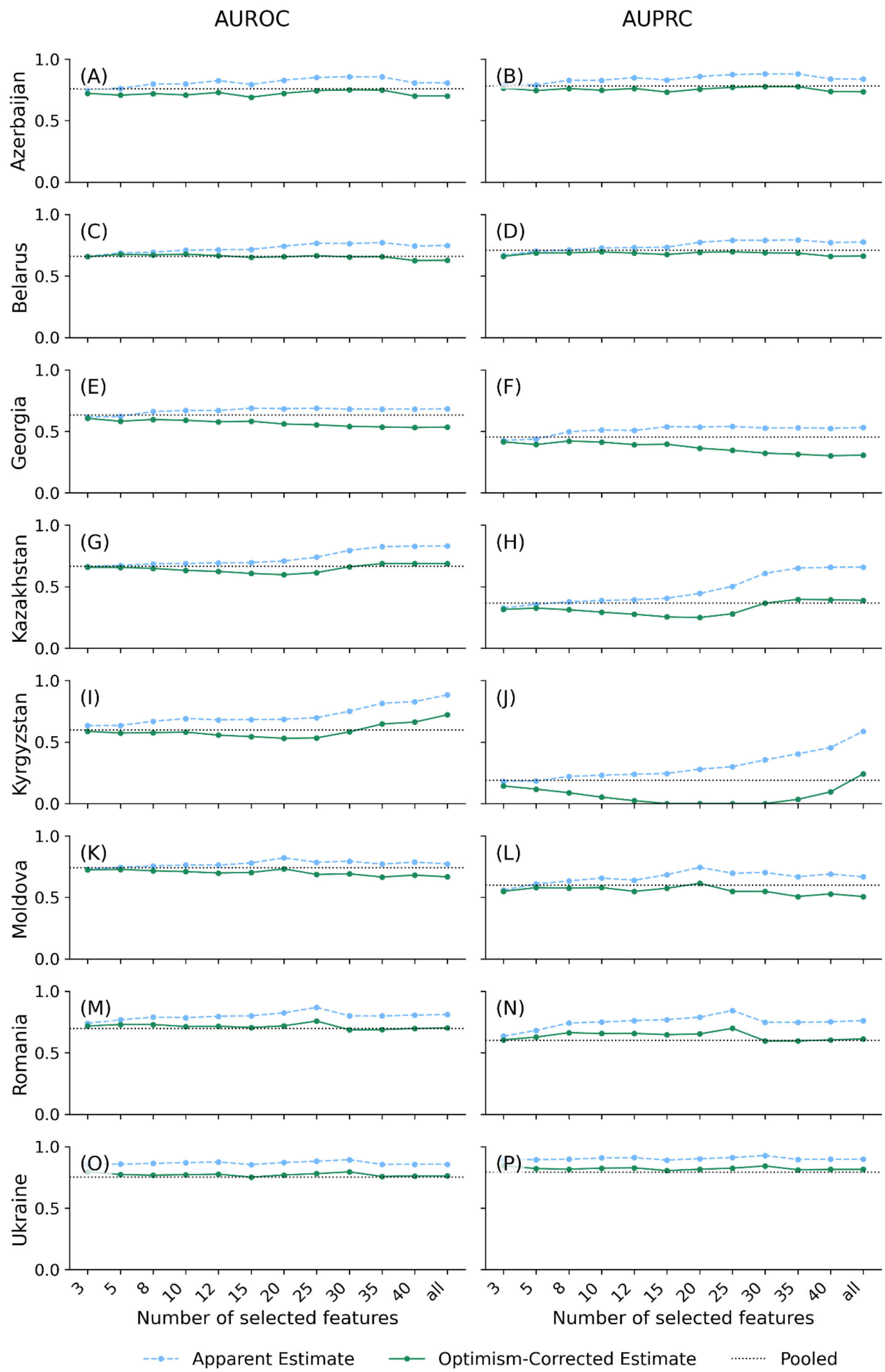


**Fig J. The performance of within-country XGBoost models by the number of selected features.** The dotted horizontal line indicates the performance of the pooled model applied to each country.

## References


1. Mohd Razali N, Yap B. Power Comparisons of Shapiro-Wilk, Kolmogorov-Smirnov, Lilliefors and Anderson-Darling Tests. J Stat Model Analytics. 2011;2.
2. Groenwold RH, Donders AR, Roes KC, Harrell FE, Jr., Moons KG. Dealing with missing outcome data in randomized trials and observational studies. Am J Epidemiol. 2012;175(3):210-7.
3. Collins GS, Moons KGM, Dhiman P, Riley RD, Beam AL, Van Calster B, et al. TRIPOD+AI statement: updated guidance for reporting clinical prediction models that use regression or machine learning methods. BMJ. 2024;385:e078378.
4. Krokva D, Mori H, Valenti S, Remez D, Hadano Y, Naito T. Analysis of the impact of crises tuberculosis incidence in Ukraine amid pandemics and war. Scientific Reports. 2025;15(1):17045.
