# Supplementary material for: Predicting resistance to fluoroquinolones among patients with rifampicin-resistant tuberculosis: A cross-country validation study
Source: PLoS Med. 2026 Sep 15;23(9):e1004965. doi: 10.1371/journal.pmed.1004965 (PMC13588531; doi:10.1371/journal.pmed.1004965)
Supplement: Supplementary file 4 [file pmed.1004965.s008.pdf]

Response to General Editorial Requests

* We ask every co-author listed on the manuscript to fill in a contributing author statement, making sure to declare all competing interests. If any of the co-authors have not filled in the statement, we will remind them to do so when the paper is revised. If all statements are not completed in a timely fashion this could hold up the re-review process. If new competing interests are declared later in the revision process, this may also hold up the submission. Should there be a problem getting one of your co-authors to fill in a statement we will be in contact. Please do not add or remove authors without first discussing this with the handling editor. You can see our competing interests policy here: https://journals.plos.org/plosmedicine/s/competing-interests.

Response: The authors have declared that no competing interests exist.

* Please upload any figures associated with your paper as individual TIF or EPS files with 300dpi resolution at resubmission; please read our figure guidelines for more information on our requirements: https://journals.plos.org/plosmedicine/s/figures. While revising your submission, we strongly recommend that you use PLOS's NAAS tool (https://ngplosjournals.pagemajik.ai/artanalysis) to test your figure files. NAAS can convert your figure files to the TIFF file type and meet basic requirements (such as print size, resolution), or provide you with a report on issues that do not meet our requirements and that NAAS cannot fix.

After uploading your figures to PLOS's NAAS tool https://ngplosjournals.pagemajik.ai/artanalysis, NAAS will process the files provided and display the results in the "Uploaded Files" section of the page as the processing is complete. If the uploaded figures meet our requirements (or NAAS is able to fix the files to meet our requirements), the figure will be marked as "fixed" above. If NAAS is unable to fix the files, a red "failed" label will appear above. When NAAS has confirmed that the figure files meet our requirements, please download the file via the download option, and include these NAAS processed figure files when submitting your revised manuscript.

Response: All figures are uploaded in TIF format.

* Please ensure that the paper adheres to the PLOS Data Availability Policy (see https://journals.plos.org/plosmedicine/s/data-availability), which requires that all data underlying the study's findings be provided in a repository or as Supporting Information. For data residing with a third party, authors are required to provide instructions with contact information (web or email address) for obtaining the data. Please note that a study author cannot be the contact person for the data. PLOS journals do not allow statements supported by "data not shown" or "unpublished results." For such statements, authors must provide supporting data or cite public sources that include it.

Response: We used data from a third party and have included the following statement in our Data statement: "Data were obtained from the TB Portals (https://tbportals.niaid.nih.gov), which is an open-access TB data resource supported by the National Institute of Allergy and Infectious Diseases (NIAID) Office of Cyber Infrastructure and Computational Biology (OCICB) in Bethesda, MD."

* We expect all researchers with submissions to PLOS in which author-generated code underpins the findings in the manuscript to make all author-generated code available without restrictions upon publication of the work. In cases where code is central to the manuscript, we require the code to be made available as a condition of publication. Authors are responsible for ensuring that the code is reusable and well documented. Please make any custom code available, either as part of your data deposition or as a supplementary file. Please add a sentence to your data availability statement regarding any code used in the study, e.g. "The code used in the analysis is available from Github [URL] and archived in Zenodo [DOI link]" Please review our guidelines

at https://journals.plos.org/plosmedicine/s/materials-software-and-code-sharing and ensure that your code is shared in a way that follows best practice and facilitates reproducibility and reuse. Because Github depositions can be readily changed or deleted, we encourage you to make a permanent DOI'd copy (e.g. in Zenodo) and provide the URL.

Response: We created a permanent DOI'd copy in Zenodo and added the following to the data statement:

> "The data used in this study are available through the TB Portals website (https://tbportals.niaid.nih.gov/access-data) following completion of a Data Use Agreement (DUA). TB Portals (https://tbportals.niaid.nih.gov), which is an open-access TB data resource supported by the National Institute of Allergy and Infectious Diseases (NIAID) Office of Cyber Infrastructure and Computational Biology (OCICB) in Bethesda, MD. These data were collected and submitted by members of the TB Portals Consortium (https://tbportals.niaid.nih.gov/Partners). Investigators and other data contributors who originally submitted the data to the TB Portals did not participate in the design or analysis of this study. The code used in the analysis can be accessed at [https://github.com/tfshcode/TB-FQ-R-Modeling]. A permanent archive is also available at Zenodo [doi.org/10.5281/zenodo.21294908]. Identifiers for the de-identified records included in this analysis are provided in the GitHub repository."

* Please ensure that the study is reported according to the TRIPOD guideline and include the completed checklist as Supporting Information. When completing the checklist, please use section and paragraph numbers, rather than page numbers. Please add the following statement, or similar, to the Methods: "This study is reported as per TRIPOD guideline (S1 Checklist)."

Response: We confirm that the study is reported according to the TRIPOD guideline and we have included the completed checklist as Supporting Information. The following is added to the Methods:

> "We developed and evaluated prediction models in accordance with the Transparent Reporting of a multivariable prediction model for Individual Prognosis Or Diagnosis (TRIPOD) recommendations (S2 TRIPOD Checklist)."

FORMATTING - GENERAL

* Abstract: Please structure your abstract using the PLOS Medicine headings (Background, Methods and Findings, Conclusions). Please combine the Methods and Findings sections into one section.

Response: The abstract is structured using the PLOS Medicine headings.

* At this stage, we ask that you include a short, non-technical Author Summary of your research to make findings accessible to a wide audience that includes both scientists and non-scientists. The Author Summary should immediately follow the Abstract in your revised manuscript. This text is subject to editorial change and should be distinct from the scientific abstract. Ideally each sub-heading should contain 2-3 single sentence, concise bullet points containing the most salient points from your study. In the final bullet point of 'What Do These Findings Mean?', please include the main limitations of the study in non-technical language. Please see our author guidelines for more information: https://journals.plos.org/plosmedicine/s/revising-your-manuscript#loc-author-summary.

Response: The Author Summary is added.

* Please express the main results with 95% CIs as well as p values. When reporting p values please report as p<0.001 and where higher as the exact p value p=0.002, for example. Throughout, suggest reporting statistical

information as follows to improve clarity for the reader "22% (95% CI [13%,28%]; p</=)". Please be sure to define all numerical values at first use.

Response: No applicable for this study.

* Please include page numbers and line numbers in the manuscript file. Use continuous line numbers (do not restart the numbering on each page).

Response: Page and line numbers have been added.

* Please cite the reference numbers in square brackets. Citations should precede punctuation.

Response: All citations are revised accordingly.

FIGURES AND TABLES
* Please provide titles and legends for all figures and tables (including those in Supporting Information files).

Response: All figure and table captions are provided. The captions for figures and tables in the Appendix are provided at the end of the document.

* Please define all abbreviations used in each figure/table (including those in Supporting Information files).

Response: All abbreviations are defined, and the list is provided below:

> **Abbreviations**: AUPRC, area under the precision-recall curve; AUROC, area under the receiver operating characteristic curve; DST, drug susceptibility testing; FQs, Fluoroquinolones; RR/MDR-TB, rifampicin-resistant or multidrug-resistant tuberculosis; TB, tuberculosis.

* Please consider avoiding the use of red and green in order to make your figure more accessible to those with color blindness.

Response: Thank you for this suggestion. We revised the main figures accordingly.

SUPPLEMENTARY MATERIAL
* Please note that supplementary material will be posted as supplied by the authors. Therefore, please amend it according to the relevant comments outlined here.

Response: We followed the comments below.

* Please cite your Supporting Information as outlined here: https://journals.plos.org/plosmedicine/s/supporting-information.

Response: We revised the manuscript in accordance with the Supporting Information guidelines.

REFERENCES
* PLOS uses the numbered citation (citation-sequence) method and first six authors, et al.

Response: We updated the reference and citation style to meet the PLOS Medicine requirements.

* Please ensure that journal name abbreviations match those found in the National Center for Biotechnology Information (NCBI) databases (https://urldefense.com/v3/__http://www.ncbi.nlm.nih.gov/nlmcatalog/journals__;!!LQC6Cpwp!oUuw1oCghdNo

0HS0D7m6jLkIFgw_uQhZZK5fqiwtrHH28vYb6QQBkTwSmwWhEbFNRjEoxcMu1uEp-NekZ5ahmg$), and are appropriately formatted and capitalised.

Response: We updated the reference and citation style to meet the PLOS Medicine requirements.

* Where website addresses are cited, please include the complete URL and specify the date of access (e.g. [accessed: 12/06/2023]).

Response: We revised the references accordingly.

* Please also see https://journals.plos.org/plosmedicine/s/submission-guidelines#loc-references for further details on reference formatting.

Response: We revised the references accordingly.

STUDY TYPE-SPECIFIC REQUESTS - not all might apply

MODELLING STUDIES

The following list is derived from Geoffrey P Garnett, Simon Cousens, Timothy B Hallett, Richard Steketee, Neff Walker. Mathematical models in the evaluation of health programmes. (2011) Lancet DOI:10.1016/S0140-6736(10)61505-X:

* If pertinent, please provide a diagram that shows the model structure, including how the natural history of the disease is represented, the process and determinants of disease acquisition, and how the putative intervention could affect the system.

Response: Not applicable.

* Please provide a complete list of model parameters, including clear and precise descriptions of the meaning of each parameter, together with the values or ranges for each, with justification or the primary source cited and important caveats about the use of these values noted.

Response: Not applicable.

* Please provide a clear statement about how the model was fitted to the data, including goodness-of-fit measure, the numerical algorithm used, which parameter varied, constraints imposed on parameter values, and starting conditions.

Response: Not applicable.

* For uncertainty analyses, please state the sources of uncertainties quantified and not quantified [can include parameter, data, and model structure].

Response: Not applicable.

* Please provide sensitivity analyses to identify which parameter values are most important in the model. Uncertainty estimates seek to derive a range of credible results on the basis of an exploration of the range of reasonable parameter values. The choice of method should be presented and justified.

Response: Not applicable.

* Please discuss the scientific rationale for the choice of model structure and identify points where this choice could influence conclusions drawn. Please also describe the strength of the scientific basis underlying the key model assumptions.

Response: Not applicable.

* For studies that develop a prediction model or evaluate its performance, please ensure that the study is reported according to the TRIPOD statement (https://urldefense.com/v3/__https://www.equator-network.org/reporting-guidelines/tripod-statement__;!!LQC6Cpwp!oUuw1oCghdNo0HS0D7m6jLkIFgw_uQhZZK5fqiwtrHH28vYb6QQBkTwSmwWhEbFNRjEoxcMu1uEp-Nd-66alpg$) and include the completed checklist as Supporting Information. Please add the following statement, or similar, to the Methods: "This study is reported as per the Transparent Reporting of a Multivariable Prediction Model for Individual Prognosis Or Diagnosis (TRIPOD) statement (S1 Checklist)." For studies using machine learning, please use the TRIPOD-AI checklist. When completing the checklist, please use section and paragraph numbers, rather than page numbers.

Response: The study is reported according to the TRIPOD statement and the TRIPOD Checklist is provided.

Responses to Reviewer 1

The manuscript addresses an important gap in the early diagnosis and appropriate treatment of preXDR-TB a serious form of TB disease which if not effectively treated can result in death. The use of machine learning modelling to determine the risk of fluoroquinolone resistance in patients with RR-TB. This will revolutionalise the approach to drug-resistant TB for national programmes especially in resource constrained countries.

The study builds on prediction models used in two countries so far to predict resistance from patient clinical and demographic data. It aims to evaluate the performance of the models in predicting resistance to fluoroquinolones and its generalisability across countries in a similar geographic area, high burden of rifampicin and fluoroquinolone resistance and low HIV prevalence.

The methodology is robust, the results and discussion are well thought through and clear however the following areas need further clarification.

I recommend that this manuscript be accepted after minor revision. The results will inform future work in this area.

Response: Thank you for your encouraging words and for reviewing our manuscript. We note that we were able to obtain more patient records from TB Portals, allowing us to extend our analysis to 8 countries: Kazakhstan, Belarus, Georgia, Moldova, Kyrgyzstan, Azerbaijan, Romania, and Ukraine.

1) On page 7, line 14 it is stated that the exclusion of the pathogen genomic data was a limitation, but no reason is provided for this even though it this has been to improve resistance prediction. Can you provide an explanation for exclusion in this context.

   Response: Thank you for pointing this out. We revised the limitation section to clarify why pathogen genomic data were not incorporated.

   > Second, we did not incorporate pathogen genomic data. While such data have been shown to improve resistance prediction, genomic sequencing remains unavailable or costly in many high-burden, resource-constrained settings. Our study intentionally focused on clinical and demographic predictors that are collected during patient intake, enabling treatment decisions at the point of care without requiring molecular testing infrastructure. Third, …

2) Considering the different diagnostic tests used for rifampicin and fluoroquinolones resistance (S1.1) and their variations in performance. Was this across countries or within countries as well? It would be good to categorise the tests used by country which would probably explain the differences in proportions of people RR-TB and FQ resistance. This could be a limitation of the study.

   Response: Thank you for this suggestion. We agree that differences in diagnostic methods for rifampicin and fluoroquinolone resistance may contribute to between-country differences in observed proportions of RR-TB and FQ resistance. We have added Table S1 to summarize the diagnostic tests used to determine rifampicin and fluoroquinolone resistance by country.

Table S1: DST methods to detect rifampicin and fluoroquinolone resistance in different countries. Methods are not mutually exclusive.

	RIF Diagnostic Tests (%)				FQ Diagnostic Tests (%)			
**Country**	Bactec	Lowenstein-Jensen	HAIN	Xpert	Bactec	Lowenstein-Jensen	HAIN	Xpert
**Azerbaijan**	54.3	81.8	8.7	65.3	37.3	72.8	1.2	-

**Belarus**	69.1	89.4	55.3	47.4	66.0	77.4	21.1	30.0
**China**	1.5	95.6	7.4	-	-	89.7	1.5	-
**Georgia**	51.1	51.8	71.0	81.7	20.7	9.1	-	18.4
**India**	81.3	-	50.0	75.0	-	-	12.5	18.8
**Kazakhstan**	69.7	2.8	22.9	76.1	93.7	2.5	2.7	27.0
**Kyrgyzstan**	92.6	-	93.2	85.9	92.1	-	0.8	84.5
**Moldova**	75.4	36.6	59.4	46.2	41.1	27.2	10.4	26.5
**Nigeria**	-	6.6	7.5	98.1	-	5.8	-	1.4
**Romania**	5.4	70.2	37.1	38.0	2.2	46.8	0.7	4.7
**Senegal**	-	-	-	93.4	-	-	-	-
**South Africa**	65.4	-	0.6	8.4	1.7	-	0.6	-
**Ukraine**	83.4	15.5	13.2	88.3	70.2	5.2	1.4	13.2

Note: Values represent the percentage of patients with each diagnostic test recorded for rifampicin or fluoroquinolone resistance determination. Because multiple diagnostic tests could be recorded for the same patient, percentages may sum to more than 100%.

We have also added the following text to the Discussion as a study limitation:

> Finally, although FQ DST coverage was high in the selected analytical sample (Table 1), the diagnostic tests used for rifampicin and fluoroquinolone resistance determination differed across countries (Table S1). Because testing practices varied by country, resistance ascertainment may not have been fully comparable across settings, potentially affecting generalizability.

3) On page 6, line 36-37 states that "Yet our analysis of permutation-based feature importance indicated……." to make this clearer I suggest rewording as follows "Yet our analysis of permutation-based feature importance for pooled and within country models (Figure 3) indicated……"

Response: Thank you for this suggestion. We revised the sentence for a clearer presentation.

4) On page 7 line 21-22: states that "If DST is not conducted randomly……" Should this not be "…conducted routinely"?

Response: Thank you for this question. What we intended to convey in this sentence is that if certain patients (e.g., patients with higher income) are systematically more likely to receive DST, prediction models that are trained on data from these patients may not be generalizable to patients who are systematically less likely to receive DST. We revised the sentence to add more clarity:

> Fourth, among the 5,357 patients with RR-TB in our dataset, about 96.6% (N=5175) had DST results for FQ resistance. If DST receipt is not random (e.g., patients with certain characteristics, such as older age or higher income, are more likely to receive DST), prediction models developed on this sample may not be generalizable to the entire population of patients with RR-TB. However, we observed a similar composition of patient characteristics among patients with RR-TB and patients with RR-TB and DST results (Table S3), which could mitigate this issue.

5) On page 4 under Data sources and study populations, paragraph 2 remove the numeric 4 between "focused and on".

<u>Response</u>: Thank you for catching this typo error. We removed the extraneous "4". The corrected text now reads "focused on".

Responses to Reviewer 2

Thank you for the opportunity to review this manuscript comparing logistic regression, neural networks, and decision trees to predict fluoroquinolone-resistant TB.

Below are some questions and concerns about the study design for the authors' consideration:

1) The authors did not appear to hold out a portion of the dataset for testing that was not used for model training, which is standard practice. Instead, a bootstrap procedure produced a dataset sampled with replacement from all available training data. Lack of a hold-out test sample suggests that final model performance metrics may not account for overfitting.

   The dramatic difference in accuracy between within-country and cross-country testing could be a reflection of this problem - since cross-country test data is, by design, withheld from model fitting.

   Response: We thank the reviewer for this important comment. We agree that accounting for overfitting when evaluating predictive performance is essential. For the pooled and within-country models, we used bootstrap optimism correction rather than a conventional train/test split. This approach follows TRIPOD recommendations for internal validation of prediction models and estimates the degree to which apparent model performance is inflated by overfitting. Specifically, for each bootstrap sample, the model was refit, evaluated both in the bootstrap sample and in the original dataset, and the average optimism was subtracted from the apparent performance to obtain optimism-corrected AUROC and AUPRC estimates. We have clarified this procedure in the Methods and Supplementary Materials. We note that the TRIPOD statement advises against using the train-test split approach and instead recommends using the bootstrap optimism correction method, which we followed in this study (https://doi.org/10.7326/M14-0698).

   We also note that our cross-country analyses constitute external validation. For these analyses, the target country was completely excluded from model training and hyperparameter selection, and model performance was evaluated only on the held-out country. Therefore, the cross-country estimates were obtained using data not involved in model development.

   Finally, we were able to obtain a larger number of patient records from TB Portals, allowing us to evaluate and compare models more accurately.

2) For models with so many parameters, the training dataset is small (n=781), and even smaller when separately fitting models for each of the 4 countries. The number of features is smaller still-- <20. Can the authors provide a statistical test or rule-of-thumb to see if their dataset is large enough for neural networks and decision trees to be appropriate tools in terms of balancing learning of generalizable patterns vs. overfitting?

   Response: Thank you for raising this important concern. We agree that model complexity relative to sample size is an important issue, particularly for neural networks and XGBoost. In the revised analysis, we have obtained a larger analytical sample of 5,175 RR-TB patients with FQ DST results across eight countries, including 1,772 patients with FQ-resistant TB. Unlike regression models, there is no single, widely accepted statistical test or rule of thumb to determine an adequate sample size for neural networks or XGBoost. We therefore addressed this concern by carefully following the recommendations of the TRIPOD statement on model development and validation procedures (https://doi.org/10.7326/M14-0698).

3) The initial data covered 13 countries, while the analysis only used data from 4-5 countries. Could inclusion of all available data for the pooled analysis have improved the model? Similarly to Kyrgyzstan,

which was was held out of the country-level modeling due to having only 7 cases of resistance, countries with few resistant cases could be used for pooled analysis only, or could be pooled regionally or by epidemic type (e.g., income group, HIV prevalence).

Response: Thank you for this comment. As mentioned above, we were able to obtain a larger number of patient records from TB Portal, allowing us to extend our analysis to eight countries (Kazakhstan, Belarus, Georgia, Moldova, Kyrgyzstan, Azerbaijan, Romania, and Ukraine) using an analytical sample of 5,175 RR-TB patients with FQ DST results.

We have also added the following paragraph, along with supplementary Figure S1 and Figure S2 (showing annual RR-TB case counts and FQ DST coverage among patients with RR-TB in the selected country-years), to clarify the criteria based on which countries are selected for our analysis:

> Our initial dataset included 17,297 TB cases from TB Portals. For our analysis, we focused on patients with confirmed RR-TB who had definitive DST results for FQs. Detailed resistance classification rules and preprocessing steps are provided in the Supplementary Section S1. The number of patients with RR-TB and FQ DST coverage among patients with RR-TB varied substantially across countries and over time (Table S2 and Figure S1). For this analysis, we included only country-level annual data with a relatively stable prevalence of RR-TB and FQ-DST coverage above 80%. The final sample included 5,175 RR-TB patients from eight countries: Kazakhstan, Belarus, Georgia, Moldova, Kyrgyzstan, Azerbaijan, Romania, and Ukraine, with registration dates between 2012 and 2024 (Table 1). Time series for the percentage of patients with RR-TB and FQ DST coverage among patients with RR-TB included in our analysis are displayed in Figure S2.

Given the larger sample, we chose to exclude the following countries that contributed a small number of patients with RR-TB and additional resistance to FLQs: Nigeria, China, India, South Africa, and Senegal. Applying models (pooled or cross-country) to these countries would result in unstable performance estimates that could not be meaningfully compared with the performance of other models in Figure 2.

4) In describing prior research on prediction of TB drug resistance, could the authors clarify if those studies only used general sociodemographic characteristics as this study did, or if they also used clinical characteristics such as symptoms and treatment adherence patterns? One would think clinical and treatment-related variables would have a better chance of predicting resistance.

Response: Thank you for the comment. Prior studies of TB drug-resistance prediction have used different types of predictors. Some studies used sociodemographic and routine clinical characteristics, while others included more detailed clinical, radiographic, laboratory, or genomic data. We agree that these additional variables may improve prediction. However, our study intentionally focused on demographic and routinely collected clinical characteristics that are more consistently collected across countries and may be available at or near the time of patient intake. We also note that, at the time of diagnosis, the patient's treatment adherence pattern is unavailable, as treatment has not yet been initiated.

We revised the limitation section to clarify why pathogen genomic data were not incorporated.

> Second, we did not incorporate pathogen genomic data. While such data have been shown to improve resistance prediction, genomic sequencing remains unavailable or costly in many high-burden, resource-constrained settings. Our study intentionally focused on clinical and demographic predictors that are collected during patient intake, enabling treatment decisions at the point of care without requiring molecular testing infrastructure. Third, …

5) This reviewer could not discern any meaningful patterns in the variable importance, other than the high importance of whether or not a TB case was new.

Response: Thank you for raising this important observation. We revised the feature importance figure to group predictors by domain to more effectively convey patterns in variable importance (Figure 3). We have also added complementary direction-based interpretation figures, including odds ratios for logistic regression and SHAP plots for the neural network and XGBoost models (Figure S3–Figure S5). The revised results confirm that case definition and treatment history variables provided the most consistent predictive signal. New TB was generally associated with a lower predicted probability of FQ resistance, whereas treatment failure and other previously treated categories were generally associated with a higher predicted probability. The contributions of demographic, comorbidity, social-risk, education, employment, and other clinical variables varied across countries and algorithms. We also revised the Results to state this pattern more directly.

> Using a permutation-based feature importance method, we identified that for pooled models, case definition variables were the most consistently important predictors across algorithms, particularly new TB and treatment failure. This finding was supported by logistic regression odds ratios and SHAP analyses for the neural network and XGBoost models (Figure S3–Figure S5). In the logistic regression models, treatment failure and other previously treated case definitions were associated with higher odds of FQ resistance, whereas new TB was generally associated with lower odds (Figure S3), a conclusion also supported by SHAP values for neural network and XGBoos models (Figure S4–Figure S5). Other variables, including BMI, number of daily contacts, number of children, comorbidity indicators, and selected social risk factors, showed smaller or less consistent contributions (Figure 3A and Figure S3–Figure S5). Country-specific models showed more heterogeneous patterns of feature importance. Case definition remained an important predictor in country-specific models, but the relative contribution of other predictors differed across countries (Figure 3B–F and Figure S3-S5). For example, the number of daily contacts contributed more strongly in Azerbaijan, whereas BMI and age of onset were more prominent in Ukraine (Figure 3, panels B and F). Some comorbidity, education, employment, and social risk variables also contributed in selected countries, although these patterns were not consistent across all algorithms or settings.

6) This reviewer's interpretation is that re-treatment cases have greater risk of drug resistance because of their prior TB drug exposure, and because prior treatment failure could be an indirect indicator of non-adherence. However, it is hard to tell without knowing the direction of the variable. Can the authors clarify this?

Response: Thank you for this comment. We agree that the previous results showed which predictors contributed to model performance, but did not clearly show the direction of association. To address this, we added directional interpretation figures in the Supplement, including odds ratios from logistic regression and SHAP value plots for the neural network and XGBoost models (Figure S3–Figure S5). These additional figures clarify the direction of case-definition and treatment-history variables: prior treatment categories, including treatment failure and relapse, were generally associated with a higher predicted probability of FQ resistance. Conversely, new TB was generally associated with a lower predicted probability of FQ resistance. These patterns are consistent with the interpretation that previous treatment and treatment failure may capture prior exposure to anti-TB drugs and a greater opportunity for resistance to emerge or be selected. However, we cannot determine the temporal relationship from these data. Patients may have developed FQ resistance during their previous treatment and, therefore, been more likely to require retreatment. Alternatively, FQ resistance may already have been present at the start of their initial treatment, contributing to the need for a subsequent course.

7) Could the authors clarify what other unifying patterns there are in Figure 3? How can one be assured that these are not just noise due to small numbers?

   Response: Thank you for raising this concern. In the revised analysis, the larger sample allowed us to examine these patterns more clearly, and the country-specific feature importance results remained heterogeneous. The most consistent pattern was the contribution of case definition, particularly new TB and treatment failure. In contrast, the importance of demographic, comorbidity, social risk, education, employment, and other clinical variables differed across countries and algorithms.

   We also compared permutation-based feature importance with logistic regression odds ratios and SHAP analyses for the neural network and XGBoost models (newly added Figure S3–Figure S5). The agreement of these complementary approaches supported the direction and importance of the case-definition variables.

8) Could Figure 3 depict bars going in positive or negative directions, depending on whether each variable is positively or negatively associated with resistance?

   Response: Thank you for this suggestion. Permutation-based feature importance quantifies the decrease in model performance after a predictor is permuted, but it does not indicate whether the predictor increases or decreases the predicted probability of FQ resistance. To address this limitation, we have included three new figures in the revised manuscript: odds ratios for the logistic regression models and SHAP analyses for the neural network and XGBoost models (Figure S3–Figure S5). These figures show whether predictor values are associated with higher or lower predicted probability of FQ resistance.

9) Finally, the overall manuscript could be improved by stating a clearer, falsifiable hypothesis. As written, it is not clear what statistical tests or benchmarks were pre-specified to ascertain whether or not models would be generalizable across countries, or to select across model types. An information criterion might be a useful metric for model selection, given the wide range in the number of degrees of freedom in each model used.

   Response: Thank you or this suggestion. We revised the first paragraph of the Methods section to state clear, falsifiable hypotheses:

   > This design allows us to investigate three hypotheses: (1) models trained within individual countries would outperform pooled multi-country models when evaluated in the same country; (2) prediction performance would decline under cross-country external validation; and (3) the importance of individual predictors would vary across countries, reflecting geographic heterogeneity in the determinants of FQ resistance.

   We also note that our main objective in this study was to evaluate the performance and transportability of prediction models. As such, we present performance estimates along with appropriate confidence intervals and assess transportability by comparing model performance across within-country, pooled, and external cross-country validation frameworks (Figure 2). Since we use AUPRC as the primary performance metric, we also used it for feature selection (mode selection) (Figure S6–Figure S9).

Responses to Reviewer 3

The manuscript addresses an important operational and clinical question by evaluating the performance and generalizability of prediction models for fluoroquinolone (FQ) resistance among RR-TB patients using routinely available demographic and clinical variables. The comparison of logistic regression, neural network, and XGBoost approaches under pooled, within-country, and cross-country validation strategies is a notable strength. The finding that prediction performance is highly context-dependent and may not generalize across settings is also relevant for implementation of data-driven decision-support tools in RR/MDR-TB care. However, several methodological and interpretational concerns require clarification.

1) This study involved secondary analysis of previously collected, open-access, de-identified data from five countries, including two from Central Asia (Kazakhstan and Kyrgyzstan) and three from Eastern Europe (Georgia, Belarus, and Moldova), selected based on high prevalence of rifampicin and fluoroquinolone resistance and data availability. The data used for analysis were collected over a prolonged 10-year period (2011–2020). However, the FQ DST coverage and prevalence of FQ resistance in the study dataset differ substantially from estimates reported in the most recent World Health Organization Global TB Report (2025).

	**Kazakhstan**	**Kyrgyzstan**	**Georgia**	**Belarus**	**Moldova**
FQ DST coverage					
WHO GTB report (2025)	82%	82%	81%	99%	95%
Study sample	9.0%	16.2%	19.4%	21.8%	15.2%
FQ resistance					
WHO GTB report (2025)	16%	18.50%	32%	27%	21%
Study sample	31.6%	7.8%	20.1	59.3	50.7

During this period, substantial changes likely occurred in RR/MDR-TB epidemiology, diagnostic coverage, treatment practices, and fluoroquinolone resistance patterns across these countries and globally. Since 2015, MDR/RR-TB diagnosis, treatment outcomes, and FQ DST coverage have improved considerably in all included countries. Treatment success rates for the 2022 MDR/RR-TB cohort reportedly exceed 74%, rifampicin resistance testing coverage is now nearly universal, RR-TB case detection has improved, and fluoroquinolone DST coverage has expanded substantially. These developments suggest that the epidemiological and programmatic context represented in earlier years of the dataset may differ substantially from current RR-TB populations. The authors should therefore discuss how expanded DST access and evolving TB program performance over time may influence the current relevance, applicability, and external validity of the proposed prediction models.

Response: Thank you for this suggestion and for pointing out the discrepancies between the study sample and the 2025 WHO GTB report. We were able to obtain a larger number of patient records from TB Portals, resulting in an analytical sample population with FQ DST coverage and FQ-resistance prevalence more consistent with the 2025 WHO GTB report, as summarized in Table 1. We also examined the distribution of RR-TB records and FQ DST coverage separately by country and year, presented in the supplementary Figures S1 and S2. To reduce the influence of highly incomplete or unstable periods, the final analysis was restricted to country-years with relatively stable RR-TB reporting and FQ DST coverage.

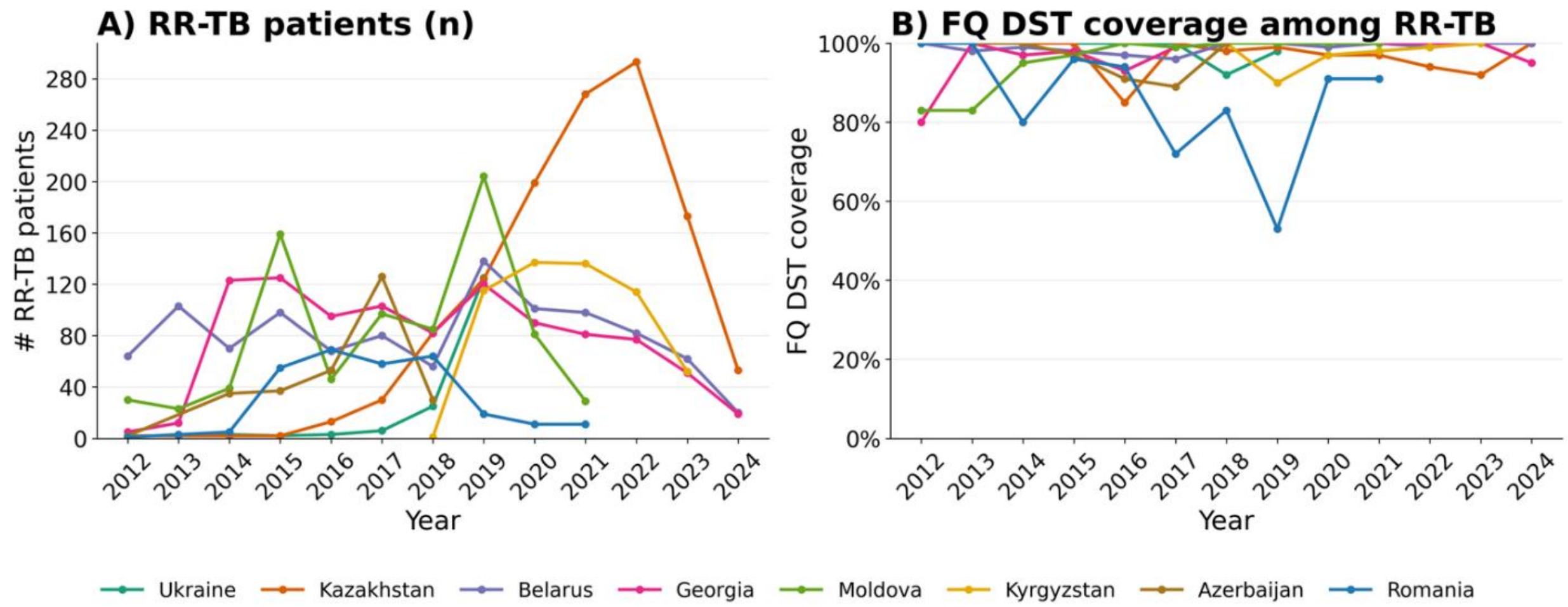

Figure S2: Annual RR-TB case counts and FQ DST coverage among patients with RR-TB in the selected country-years included in the analysis. Data shown: Ukraine 2013-2019; Kazakhstan 2012, 2014-2024; Belarus 2012-2024; Georgia 2012-2024; Moldova 2012-2021; Kyrgyzstan 2018-2023; Azerbaijan 2012, 2014-2018; Romania 2012-2021.

We have also added the following to the Discussion section as study limitations related to availability and types of DSTs.

> If DST receipt is not random (e.g., patients with certain characteristics, such as older age or higher income, are more likely to receive DST), prediction models developed on this sample may not be generalizable to the entire population of patients with RR-TB. However, we observed a similar composition of patient characteristics among patients with RR-TB and patients with RR-TB and DST results (Table S3), which could mitigate this issue. Finally, although FQ DST coverage was high in the selected analytical sample (Table 1), the diagnostic tests used for rifampicin and fluoroquinolone resistance determination differed across countries (Table S1). Because testing practices varied by country, resistance ascertainment may not have been fully comparable across settings, potentially affecting generalizability.

2) The countries included in the analysis are geographically proximate and share relatively similar epidemiological characteristics, including low HIV prevalence and high MDR/RR-TB burden. Nevertheless, the observed lack of model generalizability across these settings requires further explanation. The authors should discuss whether this reflects true biological or clinical heterogeneity in determinants of FQ resistance, or alternatively methodological limitations related to sample size, feature availability, data quality, measurement practices, or temporal changes in resistance patterns.

Response: Thank you for this insightful comment. We have expanded the Discussion to clarify that the observed limitations in model transportability likely reflect multiple, non-mutually exclusive factors.

> Although these countries share several epidemiologic characteristics, including a high burden of RR/MDR-TB, the observed limitations in transportability may reflect multiple sources of heterogeneity. These include differences in the relationships between measured predictors and FQ resistance, variation in unmeasured factors such as prior fluoroquinolone exposure and treatment history, temporal changes in resistance patterns and treatment practices over the study period, and differences in resistance ascertainment methods. Because our analysis was not designed to distinguish among these mechanisms, the observed decline in cross-country performance should be interpreted as evidence that predictor–FQ resistance relationships are not fully stable across settings rather than as evidence for any single source of heterogeneity.

3) The manuscript does not clearly describe the country-specific data collection periods or the temporal distribution of included cases. It remains unclear whether all countries contributed data uniformly across the study period or whether data originated predominantly from selected years. Given the relatively small final analytical sample (781 RR-TB cases, including 299 FQ-resistant cases), such temporal and geographic heterogeneity may substantially influence model training, validation, and interpretation of transportability.

Response: Thank you for this comment. We were able to obtain a larger number of patient records from TB Portals, increasing the analytical sample from 781 patients to 5,175 patients with RR-TB and definitive FQ DST results across eight countries. We have also added the following paragraph, along with supplementary Figure S1 and Figure S2 (showing annual RR-TB case counts and FQ DST coverage among patients with RR-TB in the selected country-years), to clarify the criteria based on which countries are selected for our analysis:

> Our initial dataset included 17,297 TB cases from TB Portals. For our analysis, we focused on patients with confirmed RR-TB who had definitive DST results for FQs. Detailed resistance classification rules and preprocessing steps are provided in the Supplementary Section S1. The number of patients with RR-TB and FQ DST coverage among patients with RR-TB varied substantially across countries and over time (Table S2 and Figure S1). For this analysis, we included only country-level annual data with a relatively stable prevalence of RR-TB and FQ-DST coverage above 80%. The final sample included 5,175 RR-TB patients from eight countries: Kazakhstan, Belarus, Georgia, Moldova, Kyrgyzstan, Azerbaijan, Romania, and Ukraine, with registration dates between 2012 and 2024 (Table 1). Time series for the percentage of patients with RR-TB and FQ DST coverage among patients with RR-TB included in our analysis are displayed in Figure S2.

Figure S2, which is also included above, displays annual RR-TB case counts and FQ DST coverage among patients with RR-TB in the selected country-years included in the analysis: Ukraine, 2013-2019; Kazakhstan, 2012 and 2014-2024; Belarus, 2012-2024; Georgia, 2012-2024; Moldova, 2012-2021; Kyrgyzstan, 2018-2023; Azerbaijan, 2012 and 2014-2018; and Romania, 2012-2021.

We acknowledge that the uneven temporal and geographic distribution of cases may still influence model development and the interpretation of cross-country transportability. We elaborate on this in the Discussion, as summarized in our responses to prior comments.

4) The initial data covered 13 countries, while the analysis only used data from 4-5 countries. Could inclusion of all available data for the pooled analysis have improved the model? Similarly to Kyrgyzstan, which was was held out of the country-level modeling due to having only 7 cases of resistance, countries with few resistant cases could be used for pooled analysis only, or could be pooled regionally or by epidemic type (e.g., income group, HIV prevalence).

Response: Thank you for this comment. As mentioned above, we were able to obtain a larger number of patient records from TB Portal, allowing us to extend our analysis to eight countries (Kazakhstan, Belarus, Georgia, Moldova, Kyrgyzstan, Azerbaijan, Romania, and Ukraine) using an analytical sample of 5,175 RR-TB patients with FQ DST results.

We have also added the following paragraph, along with supplementary Figure S1 and Figure S2 (showing annual RR-TB case counts and FQ DST coverage among patients with RR-TB in the selected country-years), to clarify the criteria based on which countries are selected for our analysis:

> Our initial dataset included 17,297 TB cases from TB Portals. For our analysis, we focused on patients with confirmed RR-TB who had definitive DST results for FQs. Detailed resistance classification rules and preprocessing steps are provided in the Supplementary Section S1. The number of patients with RR-TB and FQ DST coverage among patients with RR-TB varied substantially across countries and over time (Table S2 and Figure S1). For this analysis, we included only country-level annual data with a relatively stable prevalence of RR-TB and FQ-DST coverage above 80%. The final sample included 5,175 RR-TB patients from eight countries: Kazakhstan, Belarus, Georgia, Moldova, Kyrgyzstan, Azerbaijan, Romania, and Ukraine, with registration dates between 2012 and 2024 (Table 1). Time series for the percentage of patients with RR-TB and FQ DST coverage among patients with RR-TB included in our analysis are displayed in Figure S2.

Given the larger sample, we chose to exclude the following countries that contributed a small number of patients with RR-TB and additional resistance to FLQs: Nigeria, China, India, South Africa, and Senegal. Applying models (pooled or cross-country) to these countries would result in unstable performance estimates that could not be meaningfully compared with the performance of other models in Figure 2.

5) Although the authors acknowledge that the consistently superior performance of within-country models relative to cross-country models suggests that setting-specific factors may influence relationships between patient characteristics and FQ resistance, the manuscript would benefit from a more explicit discussion of whether the observed lack of generalizability reflects true underlying heterogeneity or limitations inherent to the dataset and modeling approach.

   Response: Thank you for this comment. In the revised analysis, performance varied across countries, algorithms, and evaluation strategies, but within-country models were not consistently superior to pooled or cross-country models (Figure 2). We have added the following text to the Discussion to discuss the factors that could impact the transportability of prediction models to detect FQ resistance.

   > Although these countries share several epidemiologic characteristics, including a high burden of RR/MDR-TB, the observed limitations in transportability may reflect multiple sources of heterogeneity. These include differences in the relationships between measured predictors and FQ resistance, variation in unmeasured factors such as prior fluoroquinolone exposure and treatment history, temporal changes in resistance patterns and treatment practices over the study period, and differences in resistance ascertainment methods. Because our analysis was not designed to distinguish among these mechanisms, the observed decline in cross-country performance should be interpreted as evidence that predictor–FQ resistance relationships are not fully stable across settings rather than as evidence for any single source of heterogeneity.

6) The manuscript would also benefit from discussion of which additional variables may improve the robustness, interpretability, and clinical relevance of future prediction models. Several potentially important predictors appear to be unavailable or inconsistently captured across countries, including prior TB treatment history, previous DR-TB treatment exposure and outcomes, prior non-TB fluoroquinolone exposure, hospitalization history, and contact history with pre-XDR/XDR-TB patients. In addition, patterns of community and over-the-counter fluoroquinolone use—which vary considerably across settings and are recognized contributors to FQ resistance—were not assessed. Inclusion of such

epidemiologically and programmatically relevant variables may improve future model performance and help explain differences in transportability across countries.

Response: Thank you for your suggestion. The current analysis was restricted to variables that were available and sufficiently harmonized across countries in TB Portals. We have added the following paragraph to the Discussion to specify additional predictors that may improve future models:

> Future prediction models may benefit from combining routine demographic and clinical variables with microbiological, genomic, radiographic, treatment history, and health system-level predictors. These may include more granular prior TB and DR-TB treatment history, prior treatment outcomes, prior exposure to non-TB fluoroquinolones, hospitalization history, contact history with patients with pre-XDR or XDR-TB, and setting-level patterns of community- or over-the-counter-use of fluoroquinolones. Such approaches may improve both predictive performance and transportability, while better clarifying the contexts in which prediction models can support FQ-resistance detection.

7) The rationale for development of prediction models for FQ resistance should be discussed in the context of rapidly expanding access to molecular DST. The authors correctly note that limited access to rapid and comprehensive DST remains a challenge in many high-burden settings globally. However, according to recent World Health Organization estimates, approximately 50% of MDR/RR-TB patients globally were tested for fluoroquinolone resistance in 2024, indicating substantial ongoing scale-up of rapid second-line DST.

   The manuscript would benefit from a balanced discussion of how expanding access to rapid molecular FQ DST may affect the future applicability, relevance, and implementation potential of such prediction models. In settings where rapid FQ DST is already widely available, the clinical utility and incremental value of prediction models based solely on demographic and clinical variables become less clear. The authors should therefore more explicitly discuss the intended role of these models in contemporary RR/MDR-TB care—for example, whether they are intended primarily for low-resource settings with limited DST access, as interim triage tools while awaiting DST results, or for epidemiological risk stratification rather than direct treatment decision-making.

   Response: Thank you for your suggestion. These models are not intended to replace molecular FQ DST where rapid testing is available. Rather, their potential role is most relevant in settings where rapid second-line DST remains unavailable, delayed, or inconsistently implemented. We have added the following text to the Discussion section to further elaborate:

   > Where rapid FQ DST is widely available, the incremental clinical utility of prediction models based only on demographic and clinical variables is likely limited. Their potential role may be greatest in settings where rapid second-line DST is unavailable, delayed, or inconsistently implemented, as interim triage tools while awaiting DST results, or for epidemiologic risk stratification and program planning. More broadly, similar prediction approaches may also be relevant for newly introduced anti-TB drugs, particularly during early implementation when validated DST is not yet widely available, and the molecular basis of resistance remains incompletely understood.
   >
   > Such approaches may improve both predictive performance and transportability, while better clarifying the contexts in which prediction models can support, but not replace, rapid FQ DST.

8) Figure 3: would benefit from improved organization and labeling of variables. Currently, predictor categories appear to be listed in a non-systematic order, making interpretation difficult. For example,

categories related to case definition (e.g., new, loss to follow-up, other) are not presented in a consistent or sequential manner across panels. Reordering variables by category or magnitude of importance, and maintaining a uniform order across pooled and country-specific panels, would substantially improve readability and facilitate comparison.

Response: Thank you for this suggestion. We revised Figure 3 to improve the organization and labeling of predictors. Predictors are now grouped into categories. Within each category, predictors are ordered by the maximum permutation-based importance.
