# Supplementary material for: Predicting resistance to fluoroquinolones among patients with rifampicin-resistant tuberculosis: A cross-country validation study
Source: PLoS Med. 2026 Sep 15;23(9):e1004965. doi: 10.1371/journal.pmed.1004965 (PMC13588531; doi:10.1371/journal.pmed.1004965)
Supplement: Supplementary file 5 [file pmed.1004965.s009.pdf]

Response to General Editorial Requests

* Please confirm that your title complies with PLOS Medicine's style. Your title must be nondeclarative and not a question. It should begin with main concept if possible. "Effect of" should be used only if causality can be inferred, i.e., for an RCT. Please place the study design ("A randomized controlled trial," "A retrospective study," "A modelling study," etc.) in the subtitle (ie, after a colon).

Response: We confirm that the tile complies with PLOS Medicine's style.

* Please confirm that your abstract complies with our requirements, including format (three sections: Background, Methods and Findings, and Conclusions) and providing all the information relevant to this study type https://journals.plos.org/plosmedicine/s/submission-guidelines#loc-abstract

Response: We confirm that the tile complies with PLOS Medicine's style.

* Please ensure that the Introduction ends with a clear description of the study question or hypothesis.

Response: The requirement is met.

* Please ensure that all abbreviations are defined at first use throughout the text.

Response: The requirement is met.

* Please confirm that all numbers presented in the abstract are present and identical to numbers presented in the main manuscript text.

Response: We confirm.

GENERAL

* Please include a statement in your manuscript regarding use or non-use of AI tools and technologies at the end of the Methods. Please specify the name(s) and version(s) of any tool(s) used and a description of their use, including how the AI-assisted or generated content was evaluated by authors, and a clear statement of which aspects of the study, article contents, data, or supporting files were affected or generated by AI tool usage. Please also include a statement specifying who retains responsibility for the final manuscript. See our guidelines for more information: https://journals.plos.org/plosmedicine/s/ethical-publishing-practice#loc-artificial-intelligence-tools-and-technologies.

Response: We added a new subsection to the end of the Methods section, as instructed:

> **AI Tools and Technologies Statement**
>
> ChatGPT (OpenAI, accessed June 2026) was used to assist with debugging portions of the analytical code and with proofreading the manuscript for grammar, spelling, and clarity. All AI-assisted code suggestions were independently reviewed and executed by the first author. All suggested language edits were reviewed and approved by all authors.

* Please review your text for claims of novelty or primacy (e.g. 'for the first time') and remove this language. In addition, please check that any use of statistical terms (such as trend or significant) are supported by the data, and if not please remove them.

Response: The requirement is met.

* Statistical reporting: Please revise throughout the manuscript, including tables and figures.
- Please report statistical information as follows to improve clarity for the reader ""22% (95% CI [13,28]; p</=)"".
- Please separate upper and lower bounds with commas instead of hyphens as the latter can be confused with reporting of negative values.
- Please repeat statistical definitions (HR, CI etc.) for each set of parentheses."

Response: We confirm that our presentation conforms to this guideline.

* In the last sentence of the Abstract Methods and Findings section, please describe the main limitation(s) of the study's methodology.

Response: We added the following sentence: “A limitation of our study is that the incidence of RR-TB and FQ resistance was relatively stable in our analysis dataset. Hence, the results may not generalize to scenarios with marked changes in MDR-TB dynamics.”

* In the author summary, in the final bullet point of 'What Do These Findings Mean?', please include the main limitations of the study in non-technical language.

Response: We added the following bullet points: “The incidence of RR-TB and FQ resistance was relatively stable in our analysis dataset, and our findings may not generalize to settings with changing MDR-TB dynamics.”

FUNDING STATEMENT

* The funding statement should include: specific grant numbers, initials of authors who received each award, URLs to sponsors’ websites. Also, please state whether any sponsors or funders (other than the named authors) played any role in study design, data collection and analysis, the decision to publish, or preparation of the manuscript. If they had no role in the research, include this sentence: “The funders had no role in study design, data collection and analysis, decision to publish, or preparation of the manuscript.”

Response: We confirm that our funding statement conforms to this guideline.

* Please move the section “Role of the funding source” after Acknowledgments section.

Response: The manuscript is updated accordingly.

COMPETING INTERESTS STATEMENT

* All authors must declare their relevant competing interests per the PLOS policy, which can be seen here: https://journals.plos.org/plosmedicine/s/competing-interests. For authors with ties to industry, please indicate whether any of the interests has a financial stake in the results of the current study.

Response: Authors declare no competing interests.

*Please amend your competing interests statement. Funding information should go to the relevant section and include URLs.

Response: The competing interests statement now only states: “The authors have declared that no competing interests exist.”

DATA AVAILABILITY
* PLOS defines the “minimal data set” to consist of the data set used to reach the conclusions drawn in the manuscript with related metadata and methods, and any additional data required to replicate the reported study findings in their entirety. Authors do not need to submit their entire data set, or the raw data collected during an investigation. Please submit the following data:
The values behind the means, standard deviations and other measures reported;
The values used to build graphs;
The points extracted from images for analysis."

Response: The data used in this study are available through the TB Portals website (https://tbportals.niaid.nih.gov/access-data) following completion of a Data Use Agreement (DUA). However, we have submitted the cleaned version of the dataset we used for our analysis as supplementary material.

FIGURES
* Please ensure that where relevant figures include 95% CIs.

Response: The requirement is met.

* Please show graph axes beginning at zero. If this is not possible, please show a break in the axis.

Response: The requirement is met.

* When a p value is given, please specify the statistical test used to determine it in the legend.

Response: Not applicable.

* Please consider avoiding the use of red and green in order to make your figure more accessible

Response: The requirement is met.

* Please provide the unadjusted comparisons as well as the adjusted comparisons in all relevant Tables

Response: Not applicable.

* Please specify the variables controlled for in all relevant Tables

Response: Not applicable.

* Please move supplementary figures and tables legends before references. Please define all acronyms used in each figure or table in its corresponding legend.

Response: The requirement is met.

ACKNOWLEDGMENTS
* Please include an acknowledgment of study participants.

Response: The acknowledgment of the study participant is now included.

* Please include in the Acknowledgments recognition of individuals who played a role in data collection or participant care or involvement

Response: The acknowledgment is revised accordingly.

CHECKLIST

* Thank you for providing TRIPOD Checklist. When completing the checklist, we require to use section and paragraph numbers, rather than page numbers (Table/Figure numbers are ok). Please revise.

Response: The TRIPOD Checklist is revised accordingly.

Responses to Reviewer 2

Thank you for responding to the comments provided by Reviewer 2. I especially appreciate the expansion of the sample size from n-781 to n=5,175. Depiction of variable importance is also much improved and easier to interpret.

This reviewer's only remaining concern is regarding the first comment from the earlier review. Notwithstanding TRIPOD, I recommend that the authors assess their model using a test/train split. If this approach is unsuccessful, it would indicate a problem with the bootstrap procedure. This reviewer suspects the bootstrap may be overly optimistic.

Response: Thank you for this suggestion. We reproduced Figure 1 using an 80/20 split design (80% of the data are used to train a model, which is then evaluated on the remaining 20%). We note that the performance of the pooled models developed and evaluated using the 80/20 design is similar to the optimism-corrected performance estimates.

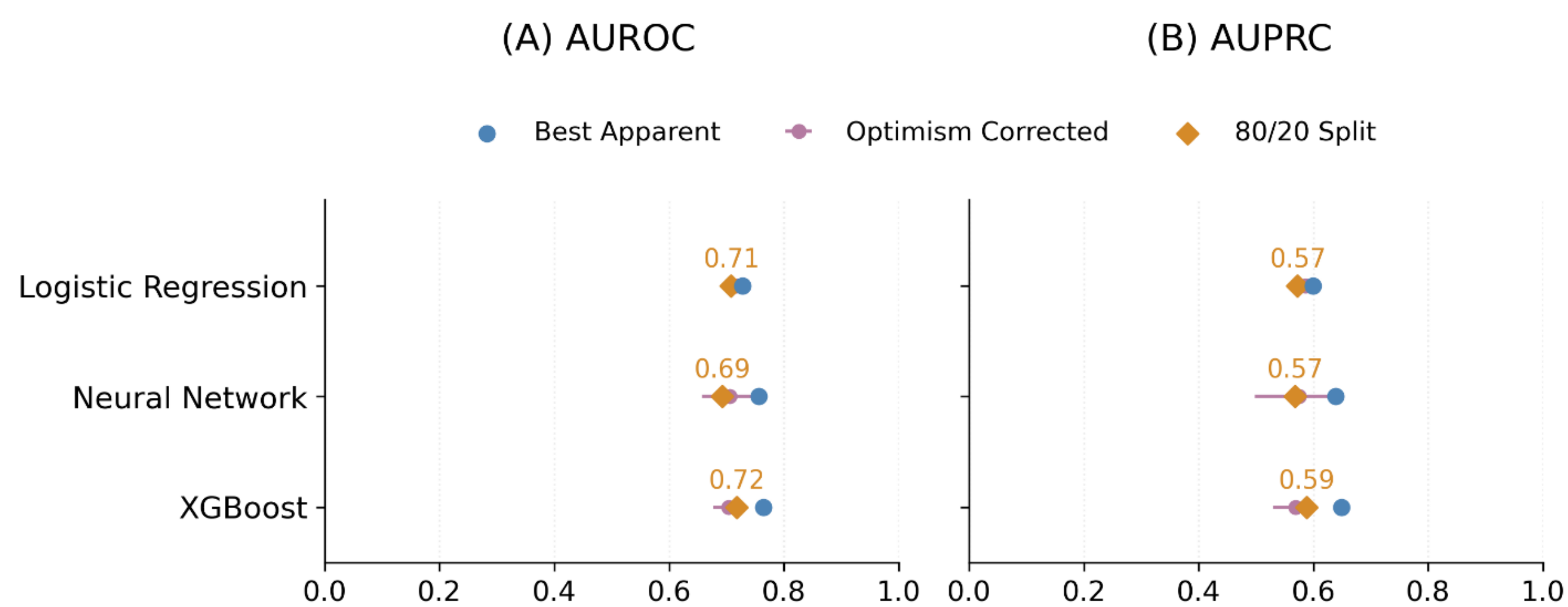

Fig S10. Apparent and optimism-corrected performance of pooled models trained on the entire dataset and the performance of a pooled model trained on 80% of the data and evaluated on the remaining 20% (80/20 split). Error bars represent 95% confidence intervals of the optimism-corrected estimates. AUROC: area under the receiver operating characteristic curve; AUPRC: area under the precision-recall curve.

Responses to Reviewer 3

The authors have responded comprehensively to the reviewers' comments and have substantially strengthened the manuscript. The expanded analysis, based on a larger dataset (>5000 RRTB patient), has improved the representativeness of the analytical cohort, with FQ DST coverage and FQ resistance prevalence now more closely reflecting the 2025 WHO Global TB Report.

I have only one minor comment regarding the wording in line 315.

Minor comment (Line 315): The statement "If DST receipt is not random" may not accurately reflect how DST is implemented in TB programmes. In routine practice, DST is generally performed either systematically for all eligible patients or selectively for predefined high-risk groups according to national guidelines and/or resource availability, rather than being "random." The authors are advised to rephrase this statement to better reflect programmatic implementation. For example:

"If DST is not performed systematically (e.g., if testing is performed selectively for patients with specific characteristics or predefined risk factors) prediction models developed on this sample may not be generalizable to the entire population of patients with RRTB.

Alternatively:

"If DST is performed only for selected groups of patients (e.g., those with specific clinical or epidemiological risk factors), prediction models developed on this sample may not be generalizable to the entire population of patients with RRTB.

Response: Thank you for raising this important point. We revised the sentence based on the Reviewer's suggestion:

> Fifth, among the 5,357 patients with RR-TB in our dataset, about 96.6% (N=5175) had DST results for FQ resistance. If DST is performed only for selected groups of patients (e.g., those with specific clinical or epidemiological risk factors), prediction models developed on this sample may not generalize to the entire population of patients with RR-TB.
