# Supplementary material for: Predicting resistance to fluoroquinolones among patients with rifampicin-resistant tuberculosis: A cross-country validation study
Source: PLoS Med. 2026 Sep 15;23(9):e1004965. doi: 10.1371/journal.pmed.1004965 (PMC13588531; doi:10.1371/journal.pmed.1004965)
Supplement: Supplementary file 6 [file pmed.1004965.s005.docx]

**Reviewer Comments for authors**

The manuscript addresses an important operational and clinical question by evaluating the performance and generalizability of prediction models for fluoroquinolone (FQ) resistance among RR-TB patients using routinely available demographic and clinical variables. The comparison of logistic regression, neural network, and XGBoost approaches under pooled, within-country, and cross-country validation strategies is a notable strength. The finding that prediction performance is highly context-dependent and may not generalize across settings is also relevant for implementation of data-driven decision-support tools in RR/MDR-TB care. However, several methodological and interpretational concerns require clarification.

This study involved secondary analysis of previously collected, open-access, de-identified data from five countries, including two from Central Asia (Kazakhstan and Kyrgyzstan) and three from Eastern Europe (Georgia, Belarus, and Moldova), selected based on high prevalence of rifampicin and fluoroquinolone resistance and data availability. The data used for analysis were collected over a prolonged 10-year period (2011–2020). However, the FQ DST coverage and prevalence of FQ resistance in the study dataset differ substantially from estimates reported in the most recent World Health Organization Global TB Report (2025).

|  | **Kazakhstan** | **Kyrgyzstan** | **Georgia** | **Belarus** | **Moldova** |
| --- | --- | --- | --- | --- | --- |
| FQ DST coverage |  |  |  |  |  |
| WHO GTB report (2025) | 82% | 82% | 81% | 99% | 95% |
| Study sample | 9.0% | 16.2% | 19.4% | 21.8% | 15.2% |
| FQ resistance |  |  |  |  |  |
| WHO GTB report (2025) | 16% | 18.50% | 32% | 27% | 21% |
| Study sample | 31.6% | 7.8% | 20.1 | 59.3 | 50.7 |

During this period, substantial changes likely occurred in RR/MDR-TB epidemiology, diagnostic coverage, treatment practices, and fluoroquinolone resistance patterns across these countries and globally. Since 2015, MDR/RR-TB diagnosis, treatment outcomes, and FQ DST coverage have improved considerably in all included countries. Treatment success rates for the 2022 MDR/RR-TB cohort reportedly exceed 74%, rifampicin resistance testing coverage is now nearly universal, RR-TB case detection has improved, and fluoroquinolone DST coverage has expanded substantially. These developments suggest that the epidemiological and programmatic context represented in earlier years of the dataset may differ substantially from current RR-TB populations. The authors should therefore discuss how expanded DST access and evolving TB program performance over time may influence the current relevance, applicability, and external validity of the proposed prediction models.

The countries included in the analysis are geographically proximate and share relatively similar epidemiological characteristics, including low HIV prevalence and high MDR/RR-TB burden. Nevertheless, the observed lack of model generalizability across these settings requires further explanation. The authors should discuss whether this reflects true biological or clinical heterogeneity in determinants of FQ resistance, or alternatively methodological limitations related to sample size, feature availability, data quality, measurement practices, or temporal changes in resistance patterns.

The manuscript does not clearly describe the country-specific data collection periods or the temporal distribution of included cases. It remains unclear whether all countries contributed data uniformly across the study period or whether data originated predominantly from selected years. Given the relatively small final analytical sample (781 RR-TB cases, including 299 FQ-resistant cases), such temporal and geographic heterogeneity may substantially influence model training, validation, and interpretation of transportability.

Although the authors acknowledge that the consistently superior performance of within-country models relative to cross-country models suggests that setting-specific factors may influence relationships between patient characteristics and FQ resistance, the manuscript would benefit from a more explicit discussion of whether the observed lack of generalizability reflects true underlying heterogeneity or limitations inherent to the dataset and modeling approach.

The manuscript would also benefit from discussion of which additional variables may improve the robustness, interpretability, and clinical relevance of future prediction models. Several potentially important predictors appear to be unavailable or inconsistently captured across countries, including prior TB treatment history, previous DR-TB treatment exposure and outcomes, prior non-TB fluoroquinolone exposure, hospitalization history, and contact history with pre-XDR/XDR-TB patients. In addition, patterns of community and over-the-counter fluoroquinolone use—which vary considerably across settings and are recognized contributors to FQ resistance—were not assessed. Inclusion of such epidemiologically and programmatically relevant variables may improve future model performance and help explain differences in transportability across countries.

**Minor comments**

1.The rationale for development of prediction models for FQ resistance should be discussed in the context of rapidly expanding access to molecular DST. The authors correctly note that limited access to rapid and comprehensive DST remains a challenge in many high-burden settings globally. However, according to recent World Health Organization estimates, approximately 50% of MDR/RR-TB patients globally were tested for fluoroquinolone resistance in 2024, indicating substantial ongoing scale-up of rapid second-line DST.

The manuscript would benefit from a balanced discussion of how expanding access to rapid molecular FQ DST may affect the future applicability, relevance, and implementation potential of such prediction models. In settings where rapid FQ DST is already widely available, the clinical utility and incremental value of prediction models based solely on demographic and clinical variables become less clear. The authors should therefore more explicitly discuss the intended role of these models in contemporary RR/MDR-TB care—for example, whether they are intended primarily for low-resource settings with limited DST access, as interim triage tools while awaiting DST results, or for epidemiological risk stratification rather than direct treatment decision-making.

2. Figure 3: would benefit from improved organization and labeling of variables. Currently, predictor categories appear to be listed in a non-systematic order, making interpretation difficult. For example, categories related to case definition (e.g., new, loss to follow-up, other) are not presented in a consistent or sequential manner across panels. Reordering variables by category or magnitude of importance, and maintaining a uniform order across pooled and country-specific panels, would substantially improve readability and facilitate comparison.

**Table: Summary of TB epidemiology, diagnostic coverage, and treatment outcomes in the five study countries based on the 2025 World Health Organization Global TB Report**

|  | **Kazakhstan** | **Kyrgyzstan** | **Georgia** | **Belarus** | **Moldova** |
| --- | --- | --- | --- | --- | --- |
|  | Central Asia | Central Asia | X-roads of Eastern Europe and Western Asia | Eastern Europe | Eastern  Europe |
| Population (M) | 20.6M | 7.2M | 3.8M | 9.1M | 3M |
| TB incidence # | 9200 | 9200 | 1700 | 1500 | 2000 |
| TB incidence per 100K | 45/100k | 128/100K | 45/100K | 17/100k | 64/100k |
|  |  |  |  |  |  |
| TB Notification | 9100 | 4000 | 1200 | 1200 | 1900 |
| CDR (treatment coverage) | 99% | 43% | 70% | 82% | 99% |
| % PTB | 90% | 81% | 87% | 94% | 90% |
| % B+ in PTB | 86% | 75% | 94% | 95% | 80% |
| **MDR/RRTB** |  |  |  |  |  |
| MDR/RRTB incidence | 3600 | 2600 | 210 | 710 | 670 |
|  | @ 18/100K | 37/100k | 5.6/100K | 7.8/100k | 22/100K |
|  | Decreased from 27 in 2015 to 18/100k | Decreased from 69 in 2015 to 37/100k | Decreased from 18 in 2015 to 5/100k | Decreased from 24 in 2015 to 7.8/100k | Decreased from 38 in 2015 to 22/100k |
| % of New TB with RRTB | 33% | 22% | 9.7% | 39% | 28% |
| % of Previously treated with RRTB | 55% | 49% | 21% | 63% | 55% |
| % tested for RR -New | 100% | 96% | 98% | 100% | 100% |
| % tested for RR -pt | 100% | 95% | 92% | 100% | 100% |
| Lab confirmed MDR/RRTB cases | 3400 | 707 | 139 | 532 | 430 |
| MDR/RR tested for FQ | 2800 | 577 | 112 | 525 | 410 |
|  | 82% | 82% | 81% | 99% | 95% |
| MDR/RRTB cases started on treatment | 4600 | 725 | 145 | 520 | 460 |
| Lab confirmed pre-XDR and XDR TB | 451 | 131 | 36 | 144 | 87 |
| % FQ resistance | 16% | 18.50% | 32% | 27% | 21% |
| TSR MDR/RRTB | 84% | 75% | 81% | 85% | 74% |
|  | Improved from 78% in 2015 to 84% in 2022 | Improved from 54% in 2015 to 75% in 2022 | Improved from 56% in 2015 to 81% in 2022 | Improved from 64% in 2015 to 85% in 2022 | Improved from 49% in 2015 to 74% in 2022 |

Data source: WHO Global TB report 2025
