# Supplementary material for: Predicting resistance to fluoroquinolones among patients with rifampicin-resistant tuberculosis: A cross-country validation study
Source: PLoS Med. 2026 Sep 15;23(9):e1004965. doi: 10.1371/journal.pmed.1004965 (PMC13588531; doi:10.1371/journal.pmed.1004965)
Supplement: Peer review history

# Editor report: Decision Letter 0

**DOI:** 10.1371/journal.pmed.1004965.r001

**Authors:** Katarzyna Starowicz

13 Feb 2026

Dear Dr Yaesoubi,

Thank you for submitting your manuscript entitled "Predicting resistance to fluoroquinolones among patients with rifampicin-resistant tuberculosis: a cross-country validation study" for consideration by PLOS Medicine.

Your manuscript has now been evaluated by the PLOS Medicine editorial staff, and I am writing to let you know that we would like to send your submission out for external peer review.

However, before we can send your manuscript to reviewers, we need you to complete your submission by providing the metadata that is required for full assessment. To this end, please login to Editorial Manager where you will find the paper in the 'Submissions Needing Revisions' folder on your homepage. Please click 'Revise Submission' from the Action Links and complete all additional questions in the submission questionnaire.

Please include completed TRIPOD checklist as supplementary document as this will help the reviewers to assess your manuscript.

For clinical studies, please upload a copy of your trial study protocol as a supporting information file. The study protocol should be the version submitted for approval to the institutional review board or ethics committee, should include any amendments to the study protocol, as well as the date of their approval by the institutional review or ethics committee. Please also detail any deviations from the study protocol in the Methods section of your manuscript. The editors will consider the protocol and study conduct prior to a final decision for external review.

Please re-submit your manuscript within two working days, i.e. by Feb 17 2026 11:59PM.

Login to Editorial Manager here: https://www.editorialmanager.com/pmedicine

Once your full submission is complete, your paper will undergo a series of checks in preparation for peer review. Once your manuscript has passed all checks it will be sent out for review.

Feel free to email us at plosmedicine@plos.org if you have any queries relating to your submission.

Kind regards,

Katarzyna Starowicz, PhD

Associate Editor

PLOS Medicine

kstarowicz@plos.org

# Peer review: Decision Letter 1

**DOI:** 10.1371/journal.pmed.1004965.r002

**Authors:** Katarzyna Starowicz

18 May 2026

Dear Dr Yaesoubi,

Many thanks for submitting your manuscript "Predicting resistance to fluoroquinolones among patients with rifampicin-resistant tuberculosis: a cross-country validation study" (PMEDICINE-D-26-00538R1) to PLOS Medicine. The paper has been reviewed by subject experts and a statistician; their comments are included below and can also be accessed here: [LINK]

After discussing the paper with the editorial team and an academic editor with relevant expertise, I'm pleased to invite you to revise the paper in response to the reviewers' comments. As you will see, the reviewers find your study of interest for the field and clinically important. All reviewers raised some concerns about methodology and analysis. We plan to send the revised paper to some or all of the original reviewers, and we cannot provide any guarantees at this stage regarding publication.

In addition to these revisions, you may need to complete some formatting changes, which you will receive in a follow up email. A member of our team will be in touch with a set of requests shortly. If you do not receive a separate email within a few days, please assume that checks have been completed, and no additional changes are required.

Please note, after reviewers comments there are listed editorial general requests and study-type specific requests at the end of this letter. You must provide point-by-point response to each of them. Some of them might not be applicable to your study, in that case please respond "not applicable". Not providing this in form of rebuttal will significantly delay your manuscript.

When you upload your revision, please include a point-by-point response that addresses all of the reviewer and editorial points (as mentioned above), indicating the changes made in the manuscript and either an excerpt of the revised text or the location (eg: page and line number) where each change can be found. Please also be sure to check the general editorial comments at the end of this letter and include these in your point-by-point response. When you resubmit your paper, please include a clean version of the paper as the main article file and a version with changes tracked as a marked-up manuscript. It may also be helpful to check the guidelines for revised papers at http://journals.plos.org/plosmedicine/s/revising-your-manuscript for any that apply to your paper.

We ask that you submit your revision by Jun 08 2026 11:59PM. However, if this deadline is not feasible, please contact me by email, and we can discuss a suitable alternative.

Don't hesitate to contact me directly with any questions (kstarowicz@plos.org).

Best regards,

Katarzyna

Katarzyna Starowicz, PhD

Associate Editor

PLOS Medicine

kstarowicz@plos.org

-----------------------------------------------------------

Comments from the reviewers:

Reviewer #2: Thank you for the opportunity to review this manuscript comparing logistic regression, neural networks, and decision trees to predict fluoroquinolone-resistant TB.

Below are some questions and concerns about the study design for the authors' consideration:

The authors did not appear to hold out a portion of the dataset for testing that was not used for model training, which is standard practice. Instead, a bootstrap procedure produced a dataset sampled with replacement from all available training data. Lack of a hold-out test sample suggests that final model performance metrics may not account for overfitting.

The dramatic difference in accuracy between within-country and cross-country testing could be a reflection of this problem - since cross-country test data is, by design, withheld from model fitting.

For models with so many parameters, the training dataset is small (n=781), and even smaller when separately fitting models for each of the 4 countries. The number of features is smaller still-- <20. Can the authors provide a statistical test or rule-of-thumb to see if their dataset is large enough for neural networks and decision trees to be appropriate tools in terms of balancing learning of generalizable patterns vs. overfitting?

The initial data covered 13 countries, while the analysis only used data from 4-5 countries. Could inclusion of all available data for the pooled analysis have improved the model? Similarly to Kyrgyzstan, which was was held out of the country-level modeling due to having only 7 cases of resistance, countries with few resistant cases could be used for pooled analysis only, or could be pooled regionally or by epidemic type (e.g., income group, HIV prevalence).

In describing prior research on prediction of TB drug resistance, could the authors clarify if those studies only used general sociodemographic characteristics as this study did, or if they also used clinical characteristics such as symptoms and treatment adherence patterns? One would think clinical and treatment-related variables would have a better chance of predicting resistance.

This reviewer could not discern any meaningful patterns in the variable importance, other than the high importance of whether or not a TB case was new.

This reviewer's interpretation is that re-treatment cases have greater risk of drug resistance because of their prior TB drug exposure, and because prior treatment failure could be an indirect indicator of non-adherence. However, it is hard to tell without knowing the direction of the variable. Can the authors clarify this?

Could the authors clarify what other unifying patterns there are in Figure 3? How can one be assured that these are not just noise due to small numbers?

Could Figure 3 depict bars going in positive or negative directions, depending on whether each variable is positively or negatively associated with resistance?

Finally, the overall manuscript could be improved by stating a clearer, falsifiable hypothesis. As written, it is not clear what statistical tests or benchmarks were pre-specified to ascertain whether or not models would be generalizable across countries, or to select across model types.

An information criterion might be a useful metric for model selection, given the wide range in the number of degrees of freedom in each model used.

Reviewer #3: The manuscript addresses an important operational and clinical question by evaluating the performance and generalizability of prediction models for fluoroquinolone (FQ) resistance among RR-TB patients using routinely available demographic and clinical variables. The comparison of logistic regression, neural network, and XGBoost approaches under pooled, within-country, and cross-country validation strategies is a notable strength. The finding that prediction performance is highly context-dependent and may not generalize across settings is also relevant for implementation of data-driven decision-support tools in RR/MDR-TB care. However, several methodological and interpretational concerns require clarification.

This study involved secondary analysis of previously collected, open-access, de-identified data from five countries, including two from Central Asia (Kazakhstan and Kyrgyzstan) and three from Eastern Europe (Georgia, Belarus, and Moldova), selected based on high prevalence of rifampicin and fluoroquinolone resistance and data availability. The data used for analysis were collected over a prolonged 10-year period (2011-2020). However, the FQ DST coverage and prevalence of FQ resistance in the study dataset differ substantially from estimates reported in the most recent World Health Organization Global TB Report (2025).

KazakhstanKyrgyzstanGeorgiaBelarusMoldova

FQ DST coverage

WHO GTB report (2025)82%82%81%99%95%

Study sample 9.0%16.2%19.4%21.8%15.2%

FQ resistance

WHO GTB report (2025)16%18.50%32%27%21%

Study sample 31.6%7.8%20.159.350.7

During this period, substantial changes likely occurred in RR/MDR-TB epidemiology, diagnostic coverage, treatment practices, and fluoroquinolone resistance patterns across these countries and globally. Since 2015, MDR/RR-TB diagnosis, treatment outcomes, and FQ DST coverage have improved considerably in all included countries. Treatment success rates for the 2022 MDR/RR-TB cohort reportedly exceed 74%, rifampicin resistance testing coverage is now nearly universal, RR-TB case detection has improved, and fluoroquinolone DST coverage has expanded substantially. These developments suggest that the epidemiological and programmatic context represented in earlier years of the dataset may differ substantially from current RR-TB populations. The authors should therefore discuss how expanded DST access and evolving TB program performance over time may influence the current relevance, applicability, and external validity of the proposed prediction models.

The countries included in the analysis are geographically proximate and share relatively similar epidemiological characteristics, including low HIV prevalence and high MDR/RR-TB burden. Nevertheless, the observed lack of model generalizability across these settings requires further explanation. The authors should discuss whether this reflects true biological or clinical heterogeneity in determinants of FQ resistance, or alternatively methodological limitations related to sample size, feature availability, data quality, measurement practices, or temporal changes in resistance patterns.

The manuscript does not clearly describe the country-specific data collection periods or the temporal distribution of included cases. It remains unclear whether all countries contributed data uniformly across the study period or whether data originated predominantly from selected years. Given the relatively small final analytical sample (781 RR-TB cases, including 299 FQ-resistant cases), such temporal and geographic heterogeneity may substantially influence model training, validation, and interpretation of transportability.

Although the authors acknowledge that the consistently superior performance of within-country models relative to cross-country models suggests that setting-specific factors may influence relationships between patient characteristics and FQ resistance, the manuscript would benefit from a more explicit discussion of whether the observed lack of generalizability reflects true underlying heterogeneity or limitations inherent to the dataset and modeling approach.

The manuscript would also benefit from discussion of which additional variables may improve the robustness, interpretability, and clinical relevance of future prediction models. Several potentially important predictors appear to be unavailable or inconsistently captured across countries, including prior TB treatment history, previous DR-TB treatment exposure and outcomes, prior non-TB fluoroquinolone exposure, hospitalization history, and contact history with pre-XDR/XDR-TB patients. In addition, patterns of community and over-the-counter fluoroquinolone use—which vary considerably across settings and are recognized contributors to FQ resistance—were not assessed. Inclusion of such epidemiologically and programmatically relevant variables may improve future model performance and help explain differences in transportability across countries.

Minor comments

1.The rationale for development of prediction models for FQ resistance should be discussed in the context of rapidly expanding access to molecular DST. The authors correctly note that limited access to rapid and comprehensive DST remains a challenge in many high-burden settings globally. However, according to recent World Health Organization estimates, approximately 50% of MDR/RR-TB patients globally were tested for fluoroquinolone resistance in 2024, indicating substantial ongoing scale-up of rapid second-line DST.

The manuscript would benefit from a balanced discussion of how expanding access to rapid molecular FQ DST may affect the future applicability, relevance, and implementation potential of such prediction models. In settings where rapid FQ DST is already widely available, the clinical utility and incremental value of prediction models based solely on demographic and clinical variables become less clear. The authors should therefore more explicitly discuss the intended role of these models in contemporary RR/MDR-TB care—for example, whether they are intended primarily for low-resource settings with limited DST access, as interim triage tools while awaiting DST results, or for epidemiological risk stratification rather than direct treatment decision-making.

2. Figure 3: would benefit from improved organization and labeling of variables. Currently, predictor categories appear to be listed in a non-systematic order, making interpretation difficult. For example, categories related to case definition (e.g., new, loss to follow-up, other) are not presented in a consistent or sequential manner across panels. Reordering variables by category or magnitude of importance, and maintaining a uniform order would substantially improve readability and facilitate comparison.

Any attachments provided with reviews can be seen via the following link: [LINK]

--------------------------------------------------------- ---

General editorial requests:

(Note: not all will apply to your paper, but please check each item carefully)

*We ask every co-author listed on the manuscript to fill in a contributing author statement, making sure to declare all competing interests. If any of the co-authors have not filled in the statement, we will remind them to do so when the paper is revised. If all statements are not completed in a timely fashion this could hold up the re-review process. If new competing interests are declared later in the revision process, this may also hold up the submission. Should there be a problem getting one of your co-authors to fill in a statement we will be in contact. Please do not add or remove authors without first discussing this with the handling editor. You can see our competing interests policy here: http://journals.plos.org/plosmedicine/s/competing-interests.

*Please upload any figures associated with your paper as individual TIF or EPS files with 300dpi resolution at resubmission; please read our figure guidelines for more information on our requirements: http://journals.plos.org/plosmedicine/s/figures. While revising your submission, we strongly recommend that you use PLOS's NAAS tool (https://ngplosjournals.pagemajik.ai/artanalysis) to test your figure files. NAAS can convert your figure files to the TIFF file type and meet basic requirements (such as print size, resolution), or provide you with a report on issues that do not meet our requirements and that NAAS cannot fix.

After uploading your figures to PLOS's NAAS tool - https://ngplosjournals.pagemajik.ai/artanalysis, NAAS will process the files provided and display the results in the "Uploaded Files" section of the page as the processing is complete.

If the uploaded figures meet our requirements (or NAAS is able to fix the files to meet our requirements), the figure will be marked as "fixed" above. If NAAS is unable to fix the files, a red "failed" label will appear above.

When NAAS has confirmed that the figure files meet our requirements, please download the file via the download option, and include these NAAS processed figure files when submitting your revised manuscript.

*Please ensure that the paper adheres to the PLOS Data Availability Policy (see http://journals.plos.org/plosmedicine/s/data-availability), which requires that all data underlying the study's findings be provided in a repository or as Supporting Information. For data residing with a third party, authors are required to provide instructions with contact information (web or email address) for obtaining the data. Please note that a study author cannot be the contact person for the data. PLOS journals do not allow statements supported by "data not shown" or "unpublished results." For such statements, authors must provide supporting data or cite public sources that include it.

*We expect all researchers with submissions to PLOS in which author-generated code underpins the findings in the manuscript to make all author-generated code available without restrictions upon publication of the work. In cases where code is central to the manuscript, we require the code to be made available as a condition of publication. Authors are responsible for ensuring that the code is reusable and well documented. Please make any custom code available, either as part of your data deposition or as a supplementary file. Please add a sentence to your data availability statement regarding any code used in the study, e.g. "The code used in the analysis is available from Github [URL] and archived in Zenodo [DOI link]" Please review our guidelines at https://journals.plos.org/plosmedicine/s/materials-software-and-code-sharing and ensure that your code is shared in a way that follows best practice and facilitates reproducibility and reuse. Because Github depositions can be readily changed or deleted, we encourage you to make a permanent DOI'd copy (e.g. in Zenodo) and provide the URL.

*Please ensure that the study is reported according to the TRIPOD guideline and include the completed checklist as Supporting Information. When completing the checklist, please use section and paragraph numbers, rather than page numbers. Please add the following statement, or similar, to the Methods: "This study is reported as per TRIPOD guideline (S1 Checklist)."

FORMATTING - GENERAL

*Abstract: Please structure your abstract using the PLOS Medicine headings (Background, Methods and Findings, Conclusions). Please combine the Methods and Findings sections into one section.

*At this stage, we ask that you include a short, non-technical Author Summary of your research to make findings accessible to a wide audience that includes both scientists and non-scientists. The Author Summary should immediately follow the Abstract in your revised manuscript. This text is subject to editorial change and should be distinct from the scientific abstract. Ideally each sub-heading should contain 2-3 single sentence, concise bullet points containing the most salient points from your study. In the final bullet point of 'What Do These Findings Mean?', please include the main limitations of the study in non-technical language. Please see our author guidelines for more information: https://journals.plos.org/plosmedicine/s/revising-your-manuscript#loc-author-summary.

*Please express the main results with 95% CIs as well as p values. When reporting p values please report as p<0.001 and where higher as the exact p value p=0.002, for example. Throughout, suggest reporting statistical information as follows to improve clarity for the reader "22% (95% CI [13%,28%]; p</=)". Please be sure to define all numerical values at first use.

*Please include page numbers and line numbers in the manuscript file. Use continuous line numbers (do not restart the numbering on each page).

*Please cite the reference numbers in square brackets. Citations should precede punctuation.

FIGURES AND TABLES

*Please provide titles and legends for all figures and tables (including those in Supporting Information files).

*Please define all abbreviations used in each figure/table (including those in Supporting Information files).

*Please consider avoiding the use of red and green in order to make your figure more accessible to those with color blindness.

SUPPLEMENTARY MATERIAL

*Please note that supplementary material will be posted as supplied by the authors. Therefore, please amend it according to the relevant comments outlined here.

*Please cite your Supporting Information as outlined here: https://journals.plos.org/plosmedicine/s/supporting-information

REFERENCES

*PLOS uses the numbered citation (citation-sequence) method and first six authors, et al.

*Please ensure that journal name abbreviations match those found in the National Center for Biotechnology Information (NCBI) databases (http://www.ncbi.nlm.nih.gov/nlmcatalog/journals), and are appropriately formatted and capitalised.

*Where website addresses are cited, please include the complete URL and specify the date of access (e.g. [accessed: 12/06/2023]).

*Please also see https://journals.plos.org/plosmedicine/s/submission-guidelines#loc-references for further details on reference formatting.

STUDY TYPE-SPECIFIC REQUESTS - not all might apply

MODELLING STUDIES

The following list is derived from Geoffrey P Garnett, Simon Cousens, Timothy B Hallett, Richard Steketee, Neff Walker. Mathematical models in the evaluation of health programmes. (2011) Lancet DOI:10.1016/S0140-6736(10)61505-X:

*If pertinent, please provide a diagram that shows the model structure, including how the natural history of the disease is represented, the process and determinants of disease acquisition, and how the putative intervention could affect the system.

*Please provide a complete list of model parameters, including clear and precise descriptions of the meaning of each parameter, together with the values or ranges for each, with justification or the primary source cited and important caveats about the use of these values noted.

*Please provide a clear statement about how the model was fitted to the data, including goodness-of-fit measure, the numerical algorithm used, which parameter varied, constraints imposed on parameter values, and starting conditions.

*For uncertainty analyses, please state the sources of uncertainties quantified and not quantified [can include parameter, data, and model structure].

*Please provide sensitivity analyses to identify which parameter values are most important in the model. Uncertainty estimates seek to derive a range of credible results on the basis of an exploration of the range of reasonable parameter values. The choice of method should be presented and justified.

*Please discuss the scientific rationale for the choice of model structure and identify points where this choice could influence conclusions drawn. Please also describe the strength of the scientific basis underlying the key model assumptions.

*For studies that develop a prediction model or evaluate its performance, please ensure that the study is reported according to the TRIPOD statement (https://www.equator-network.org/reporting-guidelines/tripod-statement) and include the completed checklist as Supporting Information. Please add the following statement, or similar, to the Methods: "This study is reported as per the Transparent Reporting of a Multivariable Prediction Model for Individual Prognosis Or Diagnosis (TRIPOD) statement (S1 Checklist)." For studies using machine learning, please use the TRIPOD-AI checklist. When completing the checklist, please use section and paragraph numbers, rather than page numbers.

Attachment Submitted filename: PMedicine-D-26-00538 Reviewer comments_for authors.docx

# Author comment: Author response to Decision Letter 2

**DOI:** 10.1371/journal.pmed.1004965.r003

26 Jun 2026

Attachment Submitted filename: PLOS Medicine Response Letter.pdf

# Editor report: Decision Letter 2

**DOI:** 10.1371/journal.pmed.1004965.r004

**Authors:** Katarzyna Starowicz

2 Jul 2026

Dear Dr Yaesoubi,

Many thanks for submitting your manuscript "Predicting resistance to fluoroquinolones among patients with rifampicin-resistant tuberculosis: a cross-country validation study" (PMEDICINE-D-26-00538R2) to PLOS Medicine.

In order to assess your resubmission prior to re-review, we require a response to all of the editors' comments in your rebuttal letter. You must provide point-by-point response to each of them. Some of them might not be applicable to your study, in that case please respond "not applicable".

These comments were listed under "General editorial requests and Study-specific request" in the previous decision letter dated 18-May-2026. For example, we note that a short, non-technical Author Summary has not been included in your resubmission. I have included all of the request again at the end of this letter after my signature.

When you upload your revision, please include a point-by-point response that addresses all of the reviewer and editorial points, indicating the changes made in the manuscript and either an excerpt of the revised text or the location (eg: page and line number) where each change can be found. Please also be sure to check the general editorial comments at the end of this letter and include these in your point-by-point response. When you resubmit your paper, please include a clean version of the paper as the main article file and a version with changes tracked as a marked-up manuscript. It may also be helpful to check the guidelines for revised papers at http://journals.plos.org/plosmedicine/s/revising-your-manuscript for any that apply to your paper.

We ask that you submit your revision by Jul 08 2026 11:59PM. However, if this deadline is not feasible, please contact me by email, and we can discuss a suitable alternative.

Don't hesitate to contact me directly with any questions (kstarowicz@plos.org).

Best regards,

Katarzyna

Katarzyna Starowicz, PhD

Associate Editor

PLOS Medicine

kstarowicz@plos.org

Any attachments provided with reviews can be seen via the following link: [LINK]

--------------------------------------------------------- ---

General editorial requests:

(Note: not all will apply to your paper, but please check each item carefully)

* We ask every co-author listed on the manuscript to fill in a contributing author statement, making sure to declare all competing interests. If any of the co-authors have not filled in the statement, we will remind them to do so when the paper is revised. If all statements are not completed in a timely fashion this could hold up the re-review process. If new competing interests are declared later in the revision process, this may also hold up the submission. Should there be a problem getting one of your co-authors to fill in a statement we will be in contact. Please do not add or remove authors without first discussing this with the handling editor. You can see our competing interests policy here: http://journals.plos.org/plosmedicine/s/competing-interests.

* Please upload any figures associated with your paper as individual TIF or EPS files with 300dpi resolution at resubmission; please read our figure guidelines for more information on our requirements: http://journals.plos.org/plosmedicine/s/figures. While revising your submission, we strongly recommend that you use PLOS's NAAS tool (https://ngplosjournals.pagemajik.ai/artanalysis) to test your figure files. NAAS can convert your figure files to the TIFF file type and meet basic requirements (such as print size, resolution), or provide you with a report on issues that do not meet our requirements and that NAAS cannot fix.

After uploading your figures to PLOS's NAAS tool - https://ngplosjournals.pagemajik.ai/artanalysis, NAAS will process the files provided and display the results in the "Uploaded Files" section of the page as the processing is complete.

If the uploaded figures meet our requirements (or NAAS is able to fix the files to meet our requirements), the figure will be marked as "fixed" above. If NAAS is unable to fix the files, a red "failed" label will appear above.

When NAAS has confirmed that the figure files meet our requirements, please download the file via the download option, and include these NAAS processed figure files when submitting your revised manuscript.

* Please ensure that the paper adheres to the PLOS Data Availability Policy (see http://journals.plos.org/plosmedicine/s/data-availability), which requires that all data underlying the study's findings be provided in a repository or as Supporting Information. For data residing with a third party, authors are required to provide instructions with contact information (web or email address) for obtaining the data. Please note that a study author cannot be the contact person for the data. PLOS journals do not allow statements supported by "data not shown" or "unpublished results." For such statements, authors must provide supporting data or cite public sources that include it.

* We expect all researchers with submissions to PLOS in which author-generated code underpins the findings in the manuscript to make all author-generated code available without restrictions upon publication of the work. In cases where code is central to the manuscript, we require the code to be made available as a condition of publication. Authors are responsible for ensuring that the code is reusable and well documented. Please make any custom code available, either as part of your data deposition or as a supplementary file. Please add a sentence to your data availability statement regarding any code used in the study, e.g. "The code used in the analysis is available from Github [URL] and archived in Zenodo [DOI link]" Please review our guidelines at https://journals.plos.org/plosmedicine/s/materials-software-and-code-sharing and ensure that your code is shared in a way that follows best practice and facilitates reproducibility and reuse. Because Github depositions can be readily changed or deleted, we encourage you to make a permanent DOI'd copy (e.g. in Zenodo) and provide the URL.

* Please ensure that the study is reported according to the TRIPOD guideline and include the completed checklist as Supporting Information. When completing the checklist, please use section and paragraph numbers, rather than page numbers. Please add the following statement, or similar, to the Methods: "This study is reported as per TRIPOD guideline (S1 Checklist)."

FORMATTING - GENERAL

* Abstract: Please structure your abstract using the PLOS Medicine headings (Background, Methods and Findings, Conclusions). Please combine the Methods and Findings sections into one section.

* At this stage, we ask that you include a short, non-technical Author Summary of your research to make findings accessible to a wide audience that includes both scientists and non-scientists. The Author Summary should immediately follow the Abstract in your revised manuscript. This text is subject to editorial change and should be distinct from the scientific abstract. Ideally each sub-heading should contain 2-3 single sentence, concise bullet points containing the most salient points from your study. In the final bullet point of 'What Do These Findings Mean?', please include the main limitations of the study in non-technical language. Please see our author guidelines for more information: https://journals.plos.org/plosmedicine/s/revising-your-manuscript#loc-author-summary.

* Please express the main results with 95% CIs as well as p values. When reporting p values please report as p<0.001 and where higher as the exact p value p=0.002, for example. Throughout, suggest reporting statistical information as follows to improve clarity for the reader "22% (95% CI [13%,28%]; p</=)". Please be sure to define all numerical values at first use.

* Please include page numbers and line numbers in the manuscript file. Use continuous line numbers (do not restart the numbering on each page).

* Please cite the reference numbers in square brackets. Citations should precede punctuation.

FIGURES AND TABLES

* Please provide titles and legends for all figures and tables (including those in Supporting Information files).

* Please define all abbreviations used in each figure/table (including those in Supporting Information files).

* Please consider avoiding the use of red and green in order to make your figure more accessible to those with color blindness.

SUPPLEMENTARY MATERIAL

* Please note that supplementary material will be posted as supplied by the authors. Therefore, please amend it according to the relevant comments outlined here.

* Please cite your Supporting Information as outlined here: https://journals.plos.org/plosmedicine/s/supporting-information

REFERENCES

* PLOS uses the numbered citation (citation-sequence) method and first six authors, et al.

* Please ensure that journal name abbreviations match those found in the National Center for Biotechnology Information (NCBI) databases (http://www.ncbi.nlm.nih.gov/nlmcatalog/journals), and are appropriately formatted and capitalised.

* Where website addresses are cited, please include the complete URL and specify the date of access (e.g. [accessed: 12/06/2023]).

* Please also see https://journals.plos.org/plosmedicine/s/submission-guidelines#loc-references for further details on reference formatting.

STUDY TYPE-SPECIFIC REQUESTS - not all might apply

MODELLING STUDIES

The following list is derived from Geoffrey P Garnett, Simon Cousens, Timothy B Hallett, Richard Steketee, Neff Walker. Mathematical models in the evaluation of health programmes. (2011) Lancet DOI:10.1016/S0140-6736(10)61505-X:

* If pertinent, please provide a diagram that shows the model structure, including how the natural history of the disease is represented, the process and determinants of disease acquisition, and how the putative intervention could affect the system.

* Please provide a complete list of model parameters, including clear and precise descriptions of the meaning of each parameter, together with the values or ranges for each, with justification or the primary source cited and important caveats about the use of these values noted.

* Please provide a clear statement about how the model was fitted to the data, including goodness-of-fit measure, the numerical algorithm used, which parameter varied, constraints imposed on parameter values, and starting conditions.

* For uncertainty analyses, please state the sources of uncertainties quantified and not quantified [can include parameter, data, and model structure].

* Please provide sensitivity analyses to identify which parameter values are most important in the model. Uncertainty estimates seek to derive a range of credible results on the basis of an exploration of the range of reasonable parameter values. The choice of method should be presented and justified.

* Please discuss the scientific rationale for the choice of model structure and identify points where this choice could influence conclusions drawn. Please also describe the strength of the scientific basis underlying the key model assumptions.

* For studies that develop a prediction model or evaluate its performance, please ensure that the study is reported according to the TRIPOD statement (https://www.equator-network.org/reporting-guidelines/tripod-statement) and include the completed checklist as Supporting Information. Please add the following statement, or similar, to the Methods: "This study is reported as per the Transparent Reporting of a Multivariable Prediction Model for Individual Prognosis Or Diagnosis (TRIPOD) statement (S1 Checklist)." For studies using machine learning, please use the TRIPOD-AI checklist. When completing the checklist, please use section and paragraph numbers, rather than page numbers.

# Author comment: Author response to Decision Letter 3

**DOI:** 10.1371/journal.pmed.1004965.r005

10 Jul 2026

Attachment Submitted filename: PLOS_Medicine_Response_Letter_auresp_3.pdf

# Peer review: Decision Letter 3

**DOI:** 10.1371/journal.pmed.1004965.r006

**Authors:** Katarzyna Starowicz

20 Aug 2026

Dear Dr. Yaesoubi,

Thank you very much for re-submitting your manuscript "Predicting resistance to fluoroquinolones among patients with rifampicin-resistant tuberculosis: a cross-country validation study" (PMEDICINE-D-26-00538R3) for review by PLOS Medicine.

I have discussed the paper with my colleagues and the academic editor and it was also seen again by two reviewers. Specifically, we have discussed remaining statistical reviewer (R2) comment. We are suggesting that you address it as supplementary analysis and in the discussion section if possible even though guidelines do not require it.

I am also pleased to say that provided the remaining editorial and production issues are dealt with we are planning to accept the paper for publication in the journal.

Please note, after reviewers comments there are listed editorial requests at the end of this letter. You must provide point-by-point response to each of them like last time. Some of them might not be applicable or you have already revised them in previous revision. Not providing this in form of rebuttal will significantly delay your manuscript.

The remaining issues that need to be addressed are listed at the end of this email. Any accompanying reviewer attachments can be seen via the link below. Please take these into account before resubmitting your manuscript:

[LINK]

***Please note while forming your response, if your article is accepted, you may have the opportunity to make the peer review history publicly available. The record will include editor decision letters (with reviews) and your responses to reviewer comments. If eligible, we will contact you to opt in or out.***

In revising the manuscript for further consideration here, please ensure you address the specific points made by each reviewer and the editors. In your rebuttal letter you should indicate your response to the reviewers' and editors' comments and the changes you have made in the manuscript. Please submit a clean version of the paper as the main article file. A version with changes marked must also be uploaded as a marked up manuscript file.

Please also check the guidelines for revised papers at http://journals.plos.org/plosmedicine/s/revising-your-manuscript for any that apply to your paper. If you haven't already, we ask that you provide a short, non-technical Author Summary of your research to make findings accessible to a wide audience that includes both scientists and non-scientists. The Author Summary should immediately follow the Abstract in your revised manuscript. This text is subject to editorial change and should be distinct from the scientific abstract.

In addition to these revisions, you may need to complete some formatting changes, which you will receive in a follow up email. A member of our team will be in touch with a set of requests shortly. If you do not receive a separate email within a few days, please assume that checks have been completed, and no additional changes are required.

We expect to receive your revised manuscript within 1 week. Please email us (plosmedicine@plos.org) if you have any questions or concerns.

We ask every co-author listed on the manuscript to fill in a contributing author statement. If any of the co-authors have not filled in the statement, we will remind them to do so when the paper is revised. If all statements are not completed in a timely fashion this could hold up the re-review process. Should there be a problem getting one of your co-authors to fill in a statement we will be in contact. YOU MUST NOT ADD OR REMOVE AUTHORS UNLESS YOU HAVE ALERTED THE EDITOR HANDLING THE MANUSCRIPT TO THE CHANGE AND THEY SPECIFICALLY HAVE AGREED TO IT.

Please ensure that the paper adheres to the PLOS Data Availability Policy (see http://journals.plos.org/plosmedicine/s/data-availability), which requires that all data underlying the study's findings be provided in a repository or as Supporting Information. For data residing with a third party, authors are required to provide instructions with contact information for obtaining the data. PLOS journals do not allow statements supported by "data not shown" or "unpublished results." For such statements, authors must provide supporting data or cite public sources that include it.

To enhance the reproducibility of your results, we recommend that you deposit your laboratory protocols in protocols.io, where a protocol can be assigned its own identifier (DOI) such that it can be cited independently in the future. Additionally, PLOS ONE offers an option to publish peer-reviewed clinical study protocols. Read more information on sharing protocols at https://plos.org/protocols?utm_medium=editorial-email&utm_source=authorletters&utm_campaign=protocols

Please review your reference list to ensure that it is complete and correct. If you have cited papers that have been retracted, please include the rationale for doing so in the manuscript text, or remove these references and replace them with relevant current references. Any changes to the reference list should be mentioned in the rebuttal letter that accompanies your revised manuscript.

Please note, when your manuscript is accepted, an uncorrected proof of your manuscript will be published online ahead of the final version, unless you've already opted out via the online submission form. If, for any reason, you do not want an earlier version of your manuscript published online or are unsure if you have already indicated as such, please let the journal staff know immediately at plosmedicine@plos.org.

If you have any questions in the meantime, please contact me or the journal staff on plosmedicine@plos.org.

We look forward to receiving the revised manuscript by Aug 27 2026 11:59PM.

Sincerely,

Katarzyna Starowicz, PhD

Associate Editor

PLOS Medicine

kstarowicz@plos.org

------------------------------------------------------------

Requests from Editors:

GENERAL EDITORIAL REQUESTS

* Please confirm that your title complies with PLOS Medicine's style. Your title must be nondeclarative and not a question. It should begin with main concept if possible. "Effect of" should be used only if causality can be inferred, i.e., for an RCT. Please place the study design ("A randomized controlled trial," "A retrospective study," "A modelling study," etc.) in the subtitle (ie, after a colon).

* Please confirm that your abstract complies with our requirements, including format (three sections: Background, Methods and Findings, and Conclusions) and providing all the information relevant to this study type https://journals.plos.org/plosmedicine/s/submission-guidelines#loc-abstract

* Please ensure that the Introduction ends with a clear description of the study question or hypothesis.

* Please ensure that all abbreviations are defined at first use throughout the text.

* Please confirm that all numbers presented in the abstract are present and identical to numbers presented in the main manuscript text.

GENERAL

* Please include a statement in your manuscript regarding use or non-use of AI tools and technologies at the end of the Methods. Please specify the name(s) and version(s) of any tool(s) used and a description of their use, including how the AI-assisted or generated content was evaluated by authors, and a clear statement of which aspects of the study, article contents, data, or supporting files were affected or generated by AI tool usage. Please also include a statement specifying who retains responsibility for the final manuscript. See our guidelines for more information: https://journals.plos.org/plosmedicine/s/ethical-publishing-practice#loc-artificial-intelligence-tools-and-technologies.

* Please review your text for claims of novelty or primacy (e.g. 'for the first time') and remove this language. In addition, please check that any use of statistical terms (such as trend or significant) are supported by the data, and if not please remove them.

* Statistical reporting: Please revise throughout the manuscript, including tables and figures.

- Please report statistical information as follows to improve clarity for the reader " "22% (95% CI [13,28]; p</=)" ".

- Please separate upper and lower bounds with commas instead of hyphens as the latter can be confused with reporting of negative values.

- Please repeat statistical definitions (HR, CI etc.) for each set of parentheses."

* In the last sentence of the Abstract Methods and Findings section, please describe the main limitation(s) of the study's methodology.

* In the author summary, in the final bullet point of 'What Do These Findings Mean?', please include the main limitations of the study in non-technical language.

FUNDING STATEMENT

* The funding statement should include: specific grant numbers, initials of authors who received each award, URLs to sponsors’ websites. Also, please state whether any sponsors or funders (other than the named authors) played any role in study design, data collection and analysis, the decision to publish, or preparation of the manuscript. If they had no role in the research, include this sentence: “The funders had no role in study design, data collection and analysis, decision to publish, or preparation of the manuscript.”

* Please move the section “Role of the funding source” after Acknowledgments section.

COMPETING INTERESTS STATEMENT

* All authors must declare their relevant competing interests per the PLOS policy, which can be seen here: https://journals.plos.org/plosmedicine/s/competing-interests For authors with ties to industry, please indicate whether any of the interests has a financial stake in the results of the current study.

*Please amend your competing interests statement. Funding information should go to the relevant section and include URLs.

DATA AVAILABILITY

* PLOS defines the “minimal data set” to consist of the data set used to reach the conclusions drawn in the manuscript with related metadata and methods, and any additional data required to replicate the reported study findings in their entirety. Authors do not need to submit their entire data set, or the raw data collected during an investigation. Please submit the following data:

The values behind the means, standard deviations and other measures reported;

The values used to build graphs;

The points extracted from images for analysis."

FIGURES

* Please ensure that where relevant figures include 95% CIs.

* Please show graph axes beginning at zero. If this is not possible, please show a break in the axis.

* When a p value is given, please specify the statistical test used to determine it in the legend.

* Please consider avoiding the use of red and green in order to make your figure more accessible

* Please provide the unadjusted comparisons as well as the adjusted comparisons in all relevant Tables

* Please specify the variables controlled for in all relevant Tables

* Please move supplementary figures and tables legends before references. Please define all acronyms used in each figure or table in its corresponding legend.

ACKNOWLEDGMENTS

* Please include an acknowledgment of study participants.

* Please include in the Acknowledgments recognition of individuals who played a role in data collection or participant care or involvement

CHECKLIST

* Thank you for providing TRIPOD Checklist. When completing the checklist, we require to use section and paragraph numbers, rather than page numbers (Table/Figure numbers are ok). Please revise.

-------------------------------------------

Comments from Reviewers:

Reviewer #2: Thank you for responding to the comments provided by Reviewer 2. I especially appreciate the expansion of the sample size from n-781 to n=5,175. Depiction of variable importance is also much improved and easier to interpret.

This reviewer's only remaining concern is regarding the first comment from the earlier review. Notwithstanding TRIPOD, I recommend that the authors assess their model using a test/train split. If this approach is unsuccessful, it would indicate a problem with the bootstrap procedure. This reviewer suspects the bootstrap may be overly optimistic.

Reviewer #3: The authors have responded comprehensively to the reviewers' comments and have substantially strengthened the manuscript. The expanded analysis, based on a larger dataset (>5000 RRTB patient), has improved the representativeness of the analytical cohort, with FQ DST coverage and FQ resistance prevalence now more closely reflecting the 2025 WHO Global TB Report.

I have only one minor comment regarding the wording in line 315.

Minor comment (Line 315): The statement "If DST receipt is not random" may not accurately reflect how DST is implemented in TB programmes. In routine practice, DST is generally performed either systematically for all eligible patients or selectively for predefined high-risk groups according to national guidelines and/or resource availability, rather than being "random." The authors are advised to rephrase this statement to better reflect programmatic implementation. For example:

"If DST is not performed systematically (e.g., if testing is performed selectively for patients with specific characteristics or predefined risk factors) prediction models developed on this sample may not be generalizable to the entire population of patients with RRTB.

Alternatively:

"If DST is performed only for selected groups of patients (e.g., those with specific clinical or epidemiological risk factors), prediction models developed on this sample may not be generalizable to the entire population of patients with RRTB.

Any attachments provided with reviews can be seen via the following link:

[LINK]

# Author comment: Author response to Decision Letter 4

**DOI:** 10.1371/journal.pmed.1004965.r007

30 Aug 2026

Attachment Submitted filename: Response Letter R2.pdf

# Editor report: Decision Letter 4

**DOI:** 10.1371/journal.pmed.1004965.r008

**Authors:** Katarzyna Starowicz

2 Sep 2026

Dear Dr Yaesoubi,

On behalf of my colleagues and the Academic Editor, Amitabh Bipin Suthar, I am pleased to inform you that we have agreed to publish your manuscript "Predicting resistance to fluoroquinolones among patients with rifampicin-resistant tuberculosis: a cross-country validation study" (PMEDICINE-D-26-00538R4) in PLOS Medicine.

Before your manuscript can be formally accepted you will need to complete some formatting changes, which you will receive in a follow up email. Please be aware that it may take several days for you to receive this email; during this time no action is required by you. Once you have received these formatting requests, please note that your manuscript will not be scheduled for publication until you have made the required changes.

In the meantime, please log into Editorial Manager at http://www.editorialmanager.com/pmedicine/, click the "Update My Information" link at the top of the page, and update your user information to ensure an efficient production process.

PRESS

We frequently collaborate with press offices. If your institution or institutions have a press office, please notify them about your upcoming paper at this point, to enable them to help maximise its impact. If the press office is planning to promote your findings, we would be grateful if they could coordinate with medicinepress@plos.org. If you have not yet opted out of the early version process, we ask that you notify us immediately of any press plans so that we may do so on your behalf.

We also ask that you take this opportunity to read our Embargo Policy regarding the discussion, promotion and media coverage of work that is yet to be published by PLOS. As your manuscript is not yet published, it is bound by the conditions of our Embargo Policy. Please be aware that this policy is in place both to ensure that any press coverage of your article is fully substantiated and to provide a direct link between such coverage and the published work. For full details of our Embargo Policy, please visit http://www.plos.org/about/media-inquiries/embargo-policy/.

To enhance the reproducibility of your results, we recommend that you deposit your laboratory protocols in protocols.io, where a protocol can be assigned its own identifier (DOI) such that it can be cited independently in the future. Additionally, PLOS ONE offers an option to publish peer-reviewed clinical study protocols. Read more information on sharing protocols at https://plos.org/protocols?utm_medium=editorial-email&utm_source=authorletters&utm_campaign=protocols

Thank you again for submitting to PLOS Medicine. We look forward to publishing your paper.

Sincerely,

Katarzyna Starowicz, PhD

Senior Editor

PLOS Medicine
